# Alcohol consumption and microvascular dysfunction: a J-shaped association: The Maastricht Study

**DOI:** 10.1186/s12933-023-01783-x

**Published:** 2023-03-24

**Authors:** Frank C. T. van der Heide, Simone J. P. M. Eussen, Alfons J. H. M. Houben, Ronald M. A. Henry, Abraham A. Kroon, Carla J. H. van der Kallen, Pieter C. Dagnelie, Martien C. J. M. van Dongen, Tos T. J. M. Berendschot, Jan S. A. G. Schouten, Carroll A. B. Webers, Marleen M. J. van Greevenbroek, Anke Wesselius, Casper G. Schalkwijk, Annemarie Koster, Jacobus F. A. Jansen, Walter H. Backes, Joline W. J. Beulens, Coen D. A. Stehouwer

**Affiliations:** 1 CARIM School for Cardiovascular Diseases, Maastricht University (UM), Maastricht, The Netherlands; 2 Department of Internal Medicine, Maastricht University Medical Center+, P. Debyelaan 25, P.O. Box 5800, 6202AZ Maastricht, The Netherlands; 3 Department of Epidemiology, UM, Maastricht, The Netherlands; 4 Heart and Vascular Center, MUMC+ Maastricht, Maastricht, The Netherlands; 5 CAPHRI Care and Public Health Research Institute, UM, Maastricht, The Netherlands; 6 University Eye Clinic Maastricht, MUMC+, Maastricht, The Netherlands; 7 Department of Ophthalmology, Canisius Wilhelmina Hospital, Nijmegen, The Netherlands; 8 Department of Epidemiology, NUTRIM School for Nutrition and Translational Research in Metabolism, UM, Maastricht, The Netherlands; 9 Department of Social Medicine, Maastricht University, Maastricht, The Netherlands; 10 School of Mental Health and Neuroscience, Maastricht University, Maastricht, The Netherlands; 11 Dept. of Radiology and Nuclear Medicine, Maastricht University Medical Centre+, Maastricht, The Netherlands; 12 Department of Epidemiology and Data Science, Amsterdam University Medical Centres – location VUmc, Amsterdam Public Health Institute, Amsterdam, The Netherlands

**Keywords:** Alcohol, Ethanol, Wine, Beer, Spirits, Microvascular dysfunction, Cerebral small vessel disease, Retinal microvascular diameters, Microvasculature, Endothelial cell dysfunction, Albuminuria, Heat-induced skin hyperemia, Flicker light-induced increase in retinal microvascular diameter, Plasma biomarkers, History of cardiovascular disease, Type 2 diabetes, Cardiovascular risk factor, Hypertension, Dyslipidemia, Smoking

## Abstract

**Background:**

Microvascular dysfunction (MVD) is an important contributor to major clinical disease such as stroke, dementia, depression, retinopathy, and chronic kidney disease. Alcohol consumption may be a determinant of MVD.

**Objective:**

Main objectives were (1) to study whether alcohol consumption was associated with MVD as assessed in the brain, retina, skin, kidney and in the blood; and (2) to investigate whether associations differed by history of cardiovascular disease or sex.

**Design:**

We used cross-sectional data from The Maastricht Study (N = 3,120 participants, 50.9% men, mean age 60 years, and 27.5% with type 2 diabetes [the latter oversampled by design]). We used regression analyses to study the association between total alcohol (per unit and in the categories, i.e. none, light, moderate, high) and MVD, where all measures of MVD were combined into a total MVD composite score (expressed in SD). We adjusted all associations for potential confounders; and tested for interaction by sex, and history of cardiovascular disease. Additionally we tested for interaction with glucose metabolism status.

**Results:**

The association between total alcohol consumption and MVD was non-linear, i.e. J-shaped. Moderate versus light total alcohol consumption was significantly associated with less MVD, after full adjustment (beta [95% confidence interval], -0.10 [-0.19; -0.01]). The shape of the curve differed with sex (P_interaction_ = 0.03), history of cardiovascular disease (P_interaction_ < 0.001), and glucose metabolism status (P_interaction_ = 0.02).

**Conclusions:**

The present cross-sectional, population-based study found evidence that alcohol consumption may have an effect on MVD. Hence, although increasing alcohol consumption cannot be recommended as a policy, this study suggests that prevention of MVD may be possible through dietary interventions.

**Supplementary Information:**

The online version contains supplementary material available at 10.1186/s12933-023-01783-x.

## Introduction

Major clinical diseases such as stroke [1], dementia [1], depression [1], retinopathy [2], and chronic kidney disease [3] are thought to be (in part) caused by microvascular dysfunction (MVD). Mechanistically, MVD is thought to hamper hemodynamic autoregulation, which can predispose capillaries to a detrimentally high pressure, leading to capillary dilation, leakage, rupture, nonperfusion (i.e. ischemia), and, ultimately, clinical symptoms of stroke [1], dementia [1], depression [1], retinopathy [2], and chronic kidney disease [3]. Biologically, MVD is thought to be to an important extent caused by an impaired endothelial cell nitric oxide (NO) bioavailability, a hall mark feature of endothelial cell dysfunction [2].

Subtle functional and structural changes of the microvasculature, which reflect (more) MVD, can be non-invasively assessed in various organs [2]. First, presence of features of cerebral small vessel disease (CSVD; i.e. greater white matter hyperintensity volume, more cerebral microbleeds, and more lacunar infarcts) can be assessed in the brain [2]. These features are thought to reflect structural deterioration of the brain and are thought to be (in part) caused by MVD [2].Second, MVD in the retina can be inferred from wider or narrower retinal arteriolar diameters, wider retinal venular diameters, or as lower flicker light-induced increase in retinal microvascular diameters [2]. The interpretation of the retinal arteriolar diameter is thought to depend on the stage of MVD, with widening as an early-stage and narrowing as a later-stage feature of MVD [2, 4]. Third, MVD in skin, kidney, and blood can respectively be assessed as lower heat-induced skin hyperemia, higher urinary albumin excretion (UAE), and higher levels of plasma biomarkers of MVD (i.e. higher levels of soluble intercellular adhesion molecule-1 [sICAM-1], soluble vascular adhesion molecule-1 [sVCAM-1], soluble E-selectin [sE-selectin] and Von Willebrand Factor [vWF]) [2].

Alcohol consumption may be a potentially modifiable determinant of MVD and many studies suggest that the association between alcohol consumption and MVD may be J-shaped [2, 5, 6]. Mechanistically, at certain lower levels of alcohol consumption, ethanol and polyphenols, the main bioactive constituents in alcoholic beverages, may be able to reduce MVD via increasing endothelial cell NO bioavailability. First, ethanol can increase NO bioavailability via stimulating NO synthesis by the endothelial cell NO synthase enzyme (eNOS) [7, 8]. Second, polyphenols are thought to increase NO bioavailability via reducing oxidative stress (oxidative stress is a potent reductor of NO bioavailability) [2, 9, 10]. Additionally, as wine and beer contain more polyphenols than spirits, wine and beer may be stronger stimulators of NO bioavailability than spirits [5]. In contrast, at certain higher levels of alcohol consumption, ethanol can induce oxidative stress [5]. Therefore, there may be a threshold where NO bioavailability is more impaired by ethanol than increased by polyphenols and ethanol, resulting in more instead of less MVD [5]. In addition, at which levels of alcohol consumption this threshold occurs and how strong the effects of alcohol consumption on MVD are may differ by background levels of oxidative stress (which are presumably higher in e.g. individuals with, versus without, a history of cardiovascular disease) [10–12] and by sex [13, 14].

Indeed, there is some evidence that alcohol consumption may be a determinant of MVD, and that the association of alcohol consumption with MVD may be J-shaped, however, this evidence has important limitations [15–53]. First, many population-based studies did not quantify the amount of alcohol consumption [15, 18, 21, 27, 28, 32, 33, 37, 40, 44, 47, 50, 52]; did not take potential cardiovascular [16–20, 22, 25, 26, 29, 34, 35, 38, 39, 41, 45, 46, 48, 49, 51, 53] and/or lifestyle [23, 24, 31, 32, 42, 43] confounders in to account; and/or did not account for sick quitters [19, 22, 30, 34, 36, 42, 43] (i.e. individuals who quit drinking and are thought to have an increased cardiovascular risk) [54, 55]. Second, only few studies investigated the associations of wine, beer, and spirits consumption with MVD [23, 26, 27, 42, 43, 45]. Third, no population-based studies have yet reported the association between alcohol consumption and MVD in individuals with and without a history of cardiovascular disease.

In view of the above, we investigated, using a large, well-characterized population-based cohort study, whether total alcohol, wine, beer, and spirits consumption were associated with MVD, estimated from features of CSVD, retinal microvascular diameters, flicker light-induced increase in retinal microvascular diameters, heat-induced skin hyperemia, UAE, and plasma biomarkers of MVD. In addition, we tested whether associations were modified by history of cardiovascular disease or sex.

## Methods

### Study population and design

The present study used data from The Maastricht Study, an observational population-based cohort study. The rationale and methodology have been described previously [56]. In brief, the study focuses on the etiology, pathophysiology, complications and comorbidities of diabetes mellitus type 2 and is characterized by an extensive phenotyping approach. Eligible for participation were all individuals aged between 40 and 75 years and living in the southern part of the Netherlands. Participants were recruited through mass media campaigns, the municipal registries and the regional Diabetes Patient Registry via mailings. Recruitment was stratified according to known type 2 diabetes status, with an oversampling of individuals with type 2 diabetes for reasons of efficiency. The present report includes cross-sectional data from 3,451 participants who completed the baseline survey between November 2010 and September 2013.

Magnetic resonance imaging (MRI) measurements were implemented from December 2013 onwards until February 2017 and were available in 2,318 out of 3,451 participants [57]. The examinations of each participant were performed within a time window of three months. The study has been approved by the institutional medical ethical committee (NL31329.068.10) and the Minister of Health, Welfare, and Sports of the Netherlands (Permit 131088–105234-PG). All participants gave written informed consent.

### Alcohol consumption

Habitual alcohol consumption over the past 12 months was assessed via a self-administered validated food frequency questionnaire (FFQ) [58]. Total alcohol consumption was calculated from the questionnaire-assessed average consumption of individual types of wine (i.e. red wine, white wine, strong wine [such as sherry]), individual types of beer (i.e. pilsner, light alcoholic beer, high alcoholic beer) and spirits [58]. The intraclass correlation coefficient for alcohol consumption assessed by FFQ versus (up to 5) 24-h recalls was 0.78 (95% confidence interval, 0.70–0.83; n = 135) [58]. We categorized alcohol consumption into none (< 1 unit per week [for both men and women]), light (≥ 1 unit/week to 1 unit/day for men, ≥ 1 unit/week to 0.5 unit/day for women), moderate (> 1 to 2 units/day for men, > 0.5 to 1 unit/day for women), and high (> 2 units/day for men, > 1 units/day for women) where 1 unit was defined as 10 g/day (g/d) of total alcohol (i.e. ethanol) consumption, 100 g/d of red or white wine consumption, 50 g/day of strong wine consumption, 225 g/d of pilsner, 320 g/d light alcoholic beer consumption, 160 g/d of high alcoholic beer consumption, or 35 g/d of spirits consumption [59].

### Features of CSVD, microvascular retinal diameters, and measures of MVD

Here, we briefly describe the methods used; a detailed description is provided in the Extended Methods (Additional file 1).

#### Features of CSVD

We evaluated three CSVD features, i.e. white matter hyperintensity volume, cerebral microbleeds, and lacunar infarcts with a 3 T brain MRI scanner (Siemens Magnetom Prisma-fit Syngo MR D13D, Erlangen, Germany).

#### Retinal microvascular diameters

We measured retinal microvascular diameters with static retinal vessel analysis from an optic disk-centered fundus photograph with the retinal health information and notification system (RHINO) software, as described previously [60]. In brief, we measured the diameter (expressed in measurement units [MU]) of the six largest retinal vessels at 0.5–1.0-disc diameter away from the optic disc margin. Diameters of arteriolar or venular vessels were combined into an average arteriolar retinal diameter (i.e. central retinal arteriolar equivalent [CRAE]) or venular retinal diameter (i.e. central retinal venular equivalent [CRVE]).

#### Flicker light-induced increase in retinal arteriolar and venular diameter

We assessed the flicker light-induced increase in retinal arteriolar and venular diameters (in MU) with the Dynamic Vessel Analyzer (Imedos, Jena, Germany), as previously described [61–63]. Briefly, a 50 s-baseline recording was consecutively followed by a 40-s flicker light exposure and a 60-s recovery period. Baseline diameter was calculated as the average diameter between 20 and 50 s of the baseline recording. The diameter during flicker light exposure was calculated as the mean of the diameters assessed at time points 10 and 40 s of flicker light stimulation exposure. Flicker light-induced increase in retinal diameter was calculated as the diameter during flicker light exposure minus the baseline diameter.

#### Heat-induced skin hyperemia

We measured heat-induced skin hyperemia by laser Doppler flowmetry (Perimed, Järfälla, Sweden), as previously described [61, 63]. Briefly, at the wrist skin blood flow, expressed in arbitrary perfusion units (PU), was recorded unheated for 2 min to serve as a baseline. After 2 min, the temperature of the laser Doppler probe was rapidly and locally increased to 44 °C and was kept constant until the end of the registration. Heat-induced increase in skin blood flow was expressed as the increase in skin blood flow during the 23 min heating phase. We calculated heat-induced increase in skin blood flow as the average skin blood flow during the 23 min heating phase minus the baseline skin blood flow (i.e. average skin blood flow during the first 2 min).

#### Urinary albumin excretion

Urinary albumin excretion (UAE) was calculated as the average UAE of two 24-h urine collections (two collections were available for 91.3% of participants). We used an automatic analyzer to measure urinary albumin concentration with a standard immunoturbidimetric assay. We multiplied urinary albumin concentration by collection volume to obtain 24-h UAE. The detection limit for assessment of urinary albumin concentration was set at 1.5 mg/L.

#### Plasma biomarkers of microvascular dysfunction

We evaluated four plasma biomarkers of microvascular dysfunction (MVD) i.e. soluble intercellular adhesion molecule-1 [sICAM-1], soluble vascular adhesion molecule-1 [sVCAM-1], soluble E-selectin [sE-selectin], and Von Willebrand Factor [vWF] [64]. sICAM-1, sVCAM-1, and sE-selectin were measured in EDTA plasma samples with commercially available 4-plex sandwich immunoassay kits with different standards and antibodies (Meso Scale Discovery, Rockville, Maryland, United States of America). vWF was quantified in citrate plasma using ELISA (Dako, Glostrup, Denmark).

### Covariates

As described previously [56], we determined glucose metabolism status according to the World Health Organization 2006 criteria as normal glucose metabolism, prediabetes, type 2 diabetes, or other types of diabetes than type 2 [65]; assessed educational level (low, intermediate, high), income level and occupational status (low, intermediate, high) as measures of socioeconomic status [66], smoking status (never, former, current), and history of cardiovascular disease by questionnaire; assessed dietary habits with the Dutch Healthy Diet index sum score, a measure of adherence to the Dutch dietary guidelines 2015 [67], based on a validated food frequency questionnaire [58]; assessed lipid-modifying, antihypertensive, and glucose-lowering medication use as part of a medication interview; assessed weight, height, waist circumference, office and 24-h ambulatory blood pressure during a physical examination; calculated body-mass index (BMI) based on body weight and height; measured total daily physical activity (hours/day) with an accelerometer [68]; measured fasting plasma glucose, 2-h post load glucose, hemoglobin A1c (HbA1c), lipid profile, serum creatinine, serum cystatin C, and plasma biomarkers of low-grade inflammation [69] (i.e. high-sensitive C-reactive protein, serum amyloid A, interleukin-6, interleukin-8 and tumor necrosis factor alpha) in fasting venous blood samples; calculated the estimated glomerular filtration rate (eGFR) with the CKD-EPI (Chronic Kidney Disease Epidemiology collaboration) formula using serum creatinine and cystatin C [70]; and assessed presence of retinopathy in both eyes via fundus photography.

### Statistical analyses

We used a total MVD composite score as endpoint. We composed a total MVD composite score because we assume that all measures of MVD under study represent a similar underlying measure of MVD [2]. In order to perform analyses we recalculated several variables. First, we inversed (i.e. multiplied by -1) flicker light-induced increase in retinal arteriolar and venular diameters and heat-induced skin hyperemia so that higher values indicate more MVD. Second, we logarithmically transformed white matter hyperintensity volume, cerebral microbleeds, lacunar infarcts, and UAE as these outcome variables were not normally distributed. Third, to reduce noise (i.e. measurement error) we calculated composite scores for CSVD features, retinal microvascular diameters, flicker light-induced increase in retinal microvascular diameters, plasma biomarkers of MVD, and total MVD [71]. To maximize the number of participants that we could use in the main analyses, we included participants in the main analyses if data were available for at least two out of six measures of MVD. Then, we performed complete cases analyses, i.e. we included individuals in the main analyses if they had complete data on the total MVD composite score, alcohol consumption, and covariates required for the main statistical models (shown below). Last, we recalculated the Dutch Healthy Diet score so that the “diet score” reflects dietary intake without alcohol consumption.

As the association between alcohol consumption and MVD may be non-linear and quadratic (i.e. J-shaped), as previously described, we tested for a quadratic association [72]. To test for a quadratic association, we entered a quadratic term of total alcohol consumption in to the model (we used the formula y = x + x^2^). If the P-value of the quadratic term was < 0.05, we considered the association statistically better described by a quadratic association than by a linear association. We performed this test for total alcohol consumption instead of individual types of alcohol for reasons of statistical power, i.e. as the range of total alcohol consumption is greater than the range of individual types of alcohol consumption, the statistical power to detect a non-linear association is likely greater [73]. In these analyses we did not exclude zero alcohol consumers (more details in the next paragraph).

We used multivariable regression analyses to analyze non-linear and linear associations of total alcohol consumption, wine, beer, and spirits consumption with the total MVD composite score.

We analyzed both linear and non-linear associations. For non-linear analyses, we entered total alcohol consumption, wine, beer, and spirits consumption into the statistical model as dummies of none, moderate, or high, versus light, alcohol consumption. Wine, beer, and spirits consumption were entered in the same model to allow mutual adjustment for consumption of other alcoholic beverages. In these analyses we used light drinkers as a reference group as we cannot distinguish so-called sick quitters from never drinkers (i.e. life-long abstainers) within the none consumers [55]. For linear analyses, we entered alcohol in the model as a continuous variable (per unit). For P for trend analyses, we entered alcohol consumption in the model as a categorical variable (coded 0 = none, 1 = light, 2 = moderate, and 3 = high alcohol consumption). For all linear analyses, we used zero drinkers as reference group. We did not use light drinkers as a reference group for linear analyses because in order to perform such analyses zero drinkers should be excluded, a methodological choice which would result in a substantial reduction in the size of the study population and a considerable loss of statistical power [73].

Model 1 shows crude results. In model 2 we adjusted for age, sex, glucose metabolism status (entered as dummies of prediabetes, type 2 diabetes, or other types of diabetes versus normal glucose metabolism status [reference]) and educational level (low [reference], middle, high). We chose these variables as they are key potential confounders (all) or because individuals were oversampled by design according to a certain condition (type 2 diabetes). In model 3A we additionally adjusted for potential confounders (waist circumference, smoking status [never {reference, current, former,}], and diet score). In model 3B we additionally adjusted for variables which are potential confounders and may additionally also be potential mediators (office systolic blood pressure, use of antihypertensive medication [yes/no], total cholesterol/HDL cholesterol ratio, lipid-modifying medication [yes/no], and history of cardiovascular disease [yes/no]). Data were expressed as regression coefficients and corresponding 95% confidence intervals.

We tested for interaction by history of cardiovascular disease and sex. We a priori hypothesized that the shape of the association may differ between individuals with and without a history of cardiovascular disease [10–12] and between men and women [13, 14]. We used a likelihood ratio test to test for interaction. The likelihood ratio test compares the goodness in fit between the fully adjusted model (model 3B) with and without an interaction term (e.g. history of cardiovascular disease*total alcohol consumption). A statistically significant P-value from the likelihood ratio test indicates that the shape of the association under study is (statistically) different between subgroups (e.g. between individuals with and without a history of cardiovascular disease).

To test robustness of our observations we performed several additional analyses. Here we highlight a selection, all additional analyses are presented in the Supplemental Methods section. First, we analyzed associations of alcohol consumption with individual measures of MVD under study. Second, we tested whether the association of total alcohol consumption with the total MVD composite score was modified by individual cardiovascular risk factors (i.e. glucose metabolism status, hypertension, current smoking, and dyslipidemia). To test whether this association was modified by individual cardiovascular risk factors, we tested for interaction by these factors. Third, we investigated the association between total alcohol consumption and the total MVD composite score in individuals with zero, one, two, three, or four of the above cardiovascular risk factors to investigate whether associations were stronger in individuals with presumed increasingly higher levels of oxidative stress. Last, we investigated how the shape of the association was impacted when we left either wine, beer, or spirits out of the total alcohol consumption index.

We performed all regression analyses with Statistical Package for Social Sciences version 23.0 (IBM SPSS, IBM Corp, Armonk, NY, USA) and likelihood ratio tests with Software for Statistics and Data Sciences version 14.0 (StataCorp, Texas, USA). For all analyses, including interaction analyses, a P-value < 0.05 was considered statistically significant.

## Results

### Selection and characteristics of the study population

**Fig. 1:**
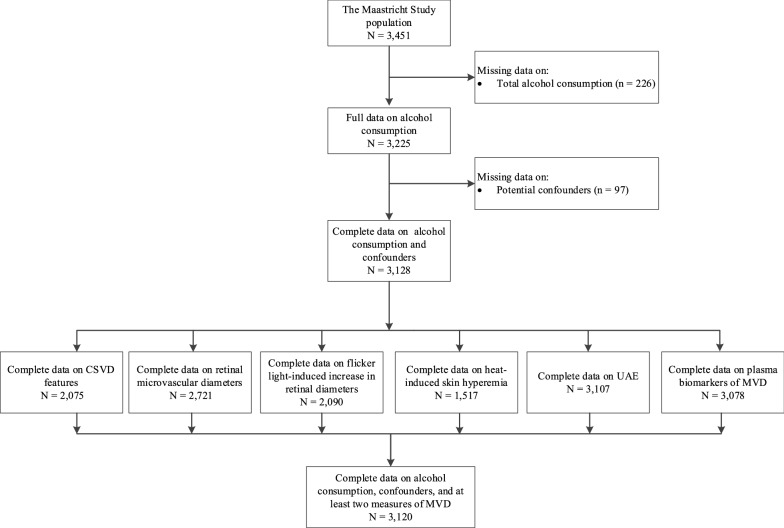
Delineates the selection of participants for inclusion. CSVD, cerebral small vessel disease; UAE, urinary albumin excretion; MVD, microvascular dysfunction

Figure 1 shows an overview of the study population selection and Tables 1A and 1B show general characteristics according to total alcohol consumption (shown for individuals with complete data on UAE [n = 3,107]). General characteristics of individuals in the study population are: mean age 60 years old, 51% men, 27.5% type 2 diabetes. Next, 16%, 31%, 20%, and 33% of participants were, respectively, none, light, moderate, and high total alcohol consumers, and 59%, 43%, and 10% of participants were, wine, beer, and/or spirits consumers, respectively. Overall, participants who consumed more alcohol were older and less likely to have type 2 diabetes. General characteristics of participants included in the study were highly comparable to those of participants with missing data (Additional file 1: Tables S1 and S2 show general characteristics of individuals who had available and missing data).

**Table 1:** General characteristics of the MVD study population in the UAE study population

Characteristic	Total study population (n = 3107)	Total alcohol consumption			
		None (n = 498)	Light (n = 964)	Moderate (n = 620)	High (n = 1,025)
Demographics					
Age, years	59.9 ± 8.2	59.0 ± 8.6	59.1 ± 8.8	60.1 ± 8.2	61.0 ± 7.2
Men	1,582 (50.9)	163 (32.7)	555 (57.6)	357 (57.6)	507 (49.5)
Lifestyle factors					
Smoking status					
Never	1088 (35.0)	214 (43.0)	379 (39.3)	236 (38.1)	259 (25.3)
Former	1,624 (52.3)	196 (39.4)	480 (49.8)	313 (50.5)	635 (62.0)
Current	395 (12.7)	88 (17.7)	105 (10.9)	71 (11.5)	131 (12.8)
Body mass index*, kg/m^2^	27.0 ± 4.5	28.2 ± 5.5	27.5 ± 4.6	26.4 ± 3.9	26.3 ± 4.0
Waist circumference, cm	95.8 ± 13.7	97.4 ± 15.7	97.3 ± 14.0	94.5 ± 12.7	94.2 ± 12.7
Physical activity*, hours/day	2.0 ± 0.7	1.9 ± 0.8	1.9 ± 0.7	2.0 ± 0.6	2.1 ± 0.7
Dutch Healthy Diet score, points	83.2 ± 14.7	84.8 ± 14.7	84.9 ± 14.1	85.7 ± 14.4	79.4 ± 14.8
Cardiovascular risk factors					
Glucose metabolism status					
Normal glucose metabolism	1,760 (56.6)	209 (42.0)	538 (55.8)	385 (62.1)	628 (61.3)
Prediabetes	460 (14.8)	59 (11.8)	137 (14.2)	90 (14.5)	174 (17.0)
Type 2 diabetes	854 (27.5)	225 (45.2)	282 (29.3)	137 (22.1)	210 (20.5)
Other types of diabetes	33 (1.1)	5 (1.0)	7 (0.7)	8 (1.3)	13 (1.3)
Fasting plasma glucose*, mmol/l	5.5 [5.1–6.5]	5.8 [5.1–7.3]	5.5 [5.0–6.7]	5.5 [5.0–6.4]	5.5 [5.1–6.2]
2-h post load plasma glucose*, mmol/l	9.3 [6.3–9.3]	7.4 [5.5–13.0]	6.3 [5.0–9.4]	6.1 [4.9–8.3]	6.1 [5.1–8.4]
HbA1c*, %	5.7 [5.4–6.2]	5.9 [5.5–6.7]	5.7 [5.3–6.3]	5.6 [5.3–6.1]	5.6 [5.3–6.0]
Use of glucose-lowering medication	697 (22.4)	196 (39.4)	244 (25.3)	102 (16.5)	155 (15.1)
Total/HDL cholesterol ratio	3.7 ± 1.2	3.8 ± 1.2	3.8 ± 1.2	3.6 ± 1.1	3.5 ± 1.2
Use of lipid-modifying medication	1,137 (36.6)	231 (46.4)	347 (36.0)	222 (35.8)	337 (32.9)
Office systolic blood pressure, mm Hg	135.0 ± 18.3	134.4 ± 18.3	134.3 ± 18.1	135.9 ± 18.4	135.5 ± 18.3
Office diastolic blood pressure, mm Hg	76.1 ± 9.9	75.0 ± 9.2	76.4 ± 10.0	76.3 ± 10.6	76.4 ± 9.8
Ambulatory systolic blood pressure, mm Hg	118.9 ± 11.7	116.7 ± 11.1	118.6 ± 11.7	119.8 ± 11.8	119.7 ± 11.7
Ambulatory diastolic blood pressure, mm Hg	73.4 ± 7.1	71.9 ± 9.0	73.7 ± 7.2	73.6 ± 7.3	73.7 ± 7.1
Use of antihypertensive medication	1,252 (40.3)	261 (52.4)	387 (40.1)	236 (38.1)	368 (35.9)
History of cardiovascular disease	522 (16.8)	115 (23.1)	175 (18.2)	101 (16.3)	131 (12.8)
Diabetic retinopathy*	41 (1.6)	9 (2.1)	20 (2.5)	6 (1.1)	6 (0.6)
eGFR, ml/min/1.73^2^	88.0 ± 14.9	86.4 ± 17.3	87.3 ± 15.5	88.6 ± 14.6	89.0 ± 13.2
Biomarkers of low-grade inflammation*					
C-reactive protein, µg/ml	1.2 [6.1–2.8]	1.7 [0.7–3.8]	1.4 [0.7–3.0]	1.2 [0.6–2.5]	1.0 [0.6–2.3]
Serum amyloid A, µg/ml	3.3 [2.1–5.4]	3.7 [2.3–6.4]	3.3 [1.9–5.7]	3.2 [2.0–5.3]	3.2 [2.1–5.1]
Tumour necrosis factor alpha, pg/ml	2.2 [1.9–2.6]	2.3 [1.9–2.7]	2.2 [1.9–2.6]	2.2 [1.9–2.5]	2.1 [1.8–2.5]
Interleukin-6, pg/ml	4.1 [3.3–5.3]	0.7 [0.5–1.0]	0.6 [0.4–0.9]	0.6 [0.4–0.8]	0.6 [0.4–0.9]
Interleukin-8, pg/ml	4.1 [3.3–5.3]	4.2 [3.4–5.4]	4.2 [3.3–5.4]	3.9 [3.2–5.3]	4.2 [3.3–5.3]
Other					
Educational status					
Low	1041 (33.5)	225 (45.2)	331 (34.3)	190 (30.6)	295 (28.8)
Medium	877 (28.2)	158 (31.7)	285 (29.6)	172 (27.7)	262 (25.6)
High	1189 (38.3)	115 (23.1)	348 (36.1)	258 (41.6)	468 (45.7)
Occupational status*					
Low	801 (31.0)	177 (46.6)	265 (32.5)	151 (28.8)	208 (20.3)
Middle	922 (35.7)	130 (34.2)	289 (35.5)	198 (37.7)	305 (35.3)
High	860 (33.3)	73 (19.2)	261 (32.0)	176 (33.5)	350 (40.6)
Income per month*, euros	2028 ± 818	1653 ± 704	1919 ± 725	2123 ± 823	2229 ± 869
Alcohol consumption					
Total alcohol consumption, units/day	0.85 [0.2–1.9]	0.0 ± 0.0	0.3 [0.1–0.5]	1.1 [0.8–1.5]	2.3 [1.8–3.1]
Total wine consumption, units/day	0.3 [0.0–1.1]	0.0 ± 0.0	0.1 [0.0–0.3]	0.6 [0.3–0.9]	1.7 [0.9–2.1]
Total beer consumption, gram/day	0.1 [0.0–0.5]	0.0 ± 0.0	0.1 [0.0–0.3]	0.3 [0.0–0.8]	0.3 [0.0–1.6]
Total spirits consumption, units/day	0.0 [0.0–0.0]	0.0 ± 0.0	0.0 [0.0–0.0]	0.0 [0.0–0.0]	0.0 [0.0–0.1]
Endpoints					
CSVD features					
White matter hyperintensity volume, ml^†^	0.0 [0.0–0.1]	0.0 [0.0–0.1]	0.0 [0.0–0.0]	0.0 [0.0–0.1]	0.0 [0.0–0.1]
Presence of cerebral microbleeds^†^	245 (11.8)	26 (8.3)	74 (11.8)	60 (14.2)	85 (11.9)
Number of cerebral microbleeds	0.0 [0.0–0.0]	0.0 [0.0–0.0]	0.0 [0.0–0.0]	0.0 [0.0–0.0]	0.0 [0.0–0.0]
Presence of lacunar infarcts^†^	114 (5.5)	20 (6.4)	37 (5.9)	21 (5.0)	36 (5.0)
Number of lacunar infarcts	0.0 [0.0–0.0]	0.0 [0.0–0.0]	0.0 [0.0–0.0]	0.0 [0.0–0.0]	0.0 [0.0–0.0]
Composite score^†^	0.0 ± 1.0	− 0.0 ± 1.0	− 0.0 ± 1.0	0.0 ± 1.0	0.0 ± 1.0
Retinal microvascular diameters					
Arteriolar diameter, MU^†^	142.3 ± 20.2	145.3 ± 19.9	143.0 ± 20.1	140.9 ± 20.7	141.1 ± 20.1
Venular diameter, MU^†^	214.6 ± 31.4	218.2 ± 31.3	215.6 ± 32.2	211.0 ± 31.4	214.0 ± 30.4
Composite score^†^	0.0 ± 1.0	− 0.0 ± 1.0	− 0.0 ± 1.0	0.1 ± 1.0	0.0 ± 1.0
Flicker light-induced increase in retinal microvascular diameters					
Arteriolar flicker light-induced dilation, MU^†^	4.4 ± 3.6	4.1 ± 3.7	4.3 ± 3.4	4.8 ± 3.8	4.3 ± 3.5
Venular flicker light-induced dilation, MU^†^	7.6 ± 4.1	7.7 ± 4.2	7.5 ± 4.0	7.5 ± 4.1	7.7 ± 4.1
Composite score†	0.0 ± 1.0	0.0 ± 1.0	0.0 ± 1.0	-0.1 ± 1.0	-0.0 ± 1.0
Heat-induced skin hyperemia, PU^†^	112.1 ± 57.3	113.1 ± 61.4	107.7 ± 53.0	109.9 ± 55.5	116.9 ± 59.8
UAE, mg/24 h^†^	6.7 [4.0–11.9]	7.3 [4.4–14.2]	6.7 [4.2–12.3]	6.4 [3.8–11.0]	6.5 [3.9–11.4]
≥ 30 mg/24 h^†^	270 (18.7)	59 (11.8)	86 (8.9)	47 (7.6)	78 (7.6)
Plasma biomarkers of MVD composite score					
sICAM-1, ng/ml^†^	353.9 ± 99.8	390.0 ± 134.2	349.2 ± 89.6	347.2 ± 95.5	344.6 ± 87.2
sVCAM-1, ng/ml^†^	428 ± 101.0	450.1 ± 125.9	432.8 ± 99.2	427.8 ± 96.3	412.9 ± 88.6
sE-selectin, ng/ml^†^	117.8 ± 65.7	131.8 ± 90.7	117.9 ± 64.2	119 ± 58.2	114.3 ± 55.0
vWF, %^†^	132.6 ± 48.4	140.4 ± 52.0	133.7 ± 47.1	131.5 ± 48.4	128.4 ± 47.3
Composite score^†^	0.0 ± 1.0	0.4 ± 1.3	0.0 ± 0.9	− 0.1 ± 1.0	− 0.1 ± 0.9

Data are presented as mean ± standard deviation, median [interquartile range] or n (%)

Definitions of alcohol consumption categories: none (< 1 unit per week [for both men and women]); light (≥ 1 unit/week to 1 unit/day for men, ≥ 1 unit/week to 0.5 unit/day for women); moderate (> 1 to 2 units/day for men, > 0.5 to 1 unit/day for women); and high (> 2 units/day for men, > 1 units/day for women)

*HbA1c* glycated hemoglobin, *HDL* high-density lipoprotein, *SD* standard deviation, *CSVD* cerebral small vessel disease, *SD* standard deviation, *MVD* microvascular dysfunction, *PU* perfusion units, *ICAM* soluble intercellular adhesion molecule-1, *sVCAM* soluble vascular adhesion molecule-1, *sE-selectin* soluble E-selectin, *vWF* von Willebrand factor, *UAE* urinary albumin excretion, *MU* measurement units

^†^value shown for study population with complete data on cerebral small vessel disease, or retinal arteriolar and venular diameters, or flicker light-induced increase in retinal arteriolar and venular diameter, or heat-induced skin hyperemia, or UAE, or plasma biomarkers of microvascular dysfunction i.e. for features of cerebral small vessel disease n = 2075; for retinal arteriolar and venular diameters n = 2721; for flicker light-induced increase in retinal arteriolar and venular diameter n = 2090; for heat-induced skin hyperemia n = 1517; for urinary albumin excretion n = 3107; and for plasma biomarkers of microvascular dysfunction n = 3078

^*^Data were available for: ambulatory blood pressure, n = 1345; BMI, n = 3106; physical activity, n = 2408; fasting plasma glucose, n = 3106; 2-h post load glucose, n = 2868; HbA1c, n = 3100; diabetic retinopathy, n = 2610; eGFR, n = 3082; biomarkers of low grade-inflammation, n = 2079; occupational status, n = 2,583; income, n = 2,368

#### Associations between alcohol consumption and measures of MVD

**Fig. 2:**
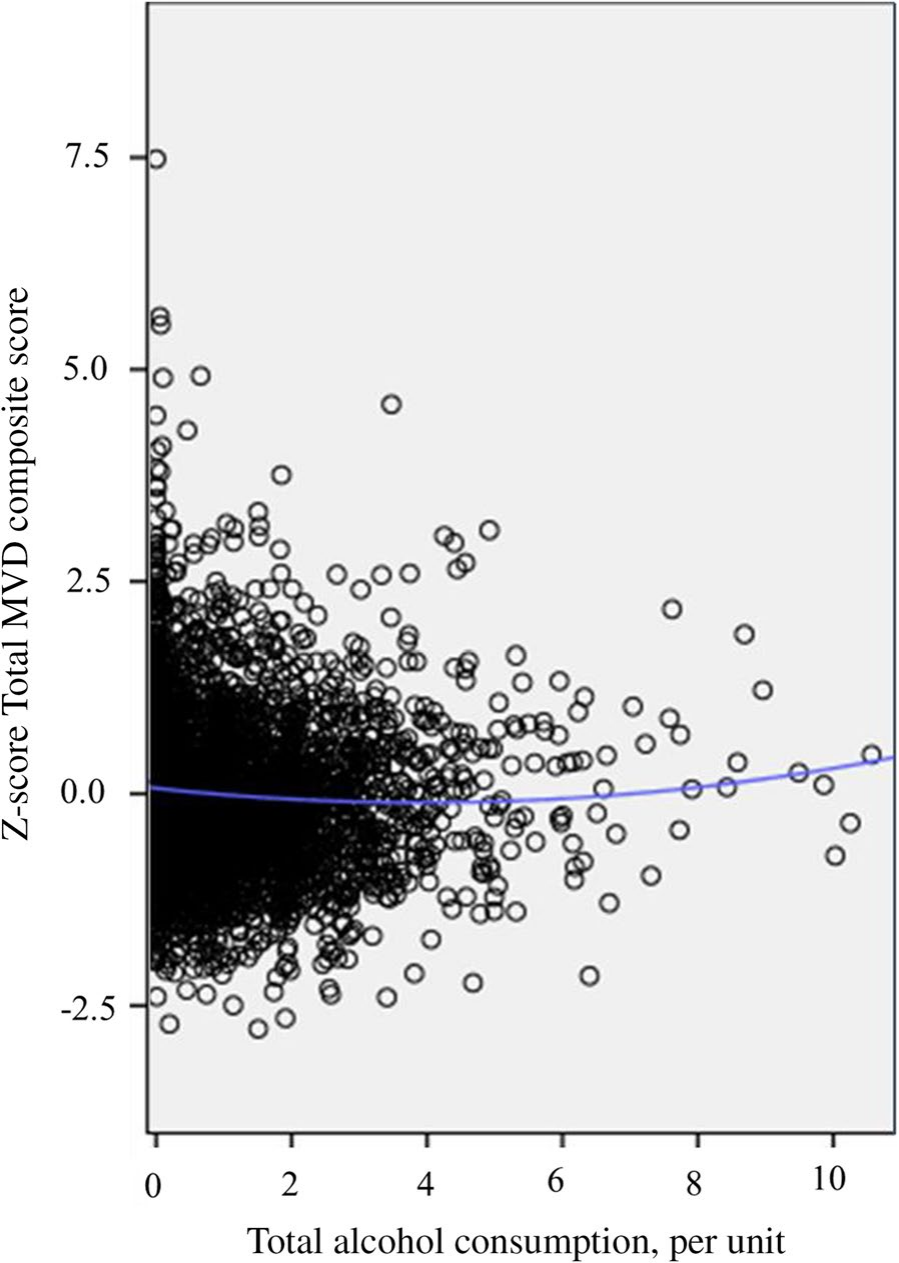
General population (N = 3,120; minimum of the J-curve at 4 units/day)**.** Figure 2 The Scatter plot shows data points for total alcohol consumption (x-axis; per unit) and the total MVD composite score (y-axis; in SD) where a quadratic association was modeled (blue line). In the general population the minimum of the J-curve was located at approximately 4 units/day

**Fig. 3:**
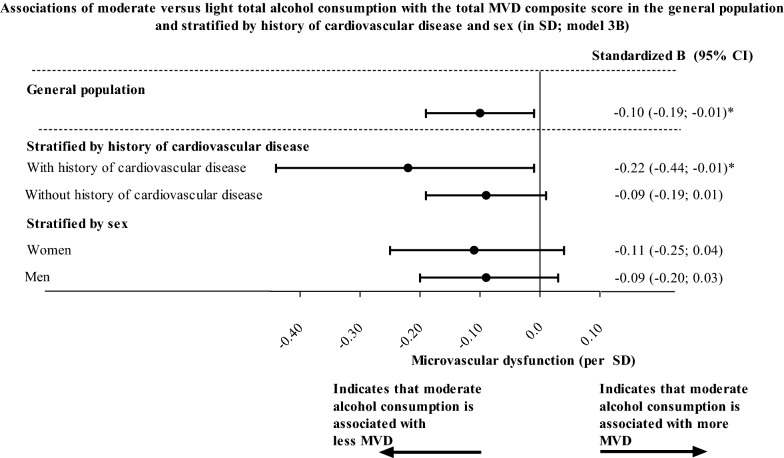
Associations of moderate versus light total alcohol consumption with the total MVD composite score in the general population. Betas and 95% confidence intervals indicate the strength of the associations of total alcohol consumption (moderate versus light) with total MVD composite score (per SD) where a negative beta indicates less MVD. The number of participants in analyses and the numerical values per SD for all endpoints are reported in the legends of Table 2 (general population), Additional file 1: Table S4 (history of cardiovascular disease strata) and Additional file 1: Table S5 (sex strata). Variables included in model 3B are age, sex (where applicable), glucose metabolism status, education level, waist circumference, smoking status, diet score, office systolic blood pressure, use of antihypertensive medication, total cholesterol/HDL cholesterol ratio, lipid-modifying medication, and history of cardiovascular disease (where applicable). *indicates P-value < 0.05. B, beta; CI: confidence interval; SD: standard deviation; MVD, microvascular dysfunction

The association between total alcohol consumption and MVD was non-linear, i.e. J-shaped (model 3B; P_quadratic_-value = 0.01; Fig. 2). The mathematical minimum of the J-curve (“minimum”) was at approximately 4 units/day in the crude model (i.e. the amount of total alcohol consumption where the association becomes directionally different; Fig. 2). After full adjustment (model 3B), moderate versus light total alcohol, wine, beer, and spirits consumption was statistically significantly associated with less MVD (model 3B; moderate versus light total alcohol, wine, beer, and spirits consumption, respectively; standardized betas [95% confidence interval], − 0.10 [− 0.19; − 0.01]; − 0.15 [− 0.25; − 0.05]; − 0.13 [− 0.25; − 0.02]; and − 0.16 [− 0.29; − 0.04]; Table 2 and Fig. 3).

**Table 2:** Associations of total alcohol, wine, beer, and spirits consumption with the total MVD composite score in the general population

	Alcohol consumption					
	Model	Continuous	None vs. light	Moderate vs. light	High vs. light	P for trend
		β (95% CI)	β (95% CI)	β (95% CI)	β (95% CI)	P−value
General population, n = 3120						
Total alcohol consumption	1 2 3A 3B	− 0.02 (− 0.05; 0.00) **− 0.05 (− 0.07; − 0.02)** **− 0.05 (− 0.08; − 0.03)** **− 0.04 (− 0.07; − 0.02)**	**0.23 (− 0.12; 0.33)** **0.16 (0.06; 0.26)** **0.15 (0.05; 0.25)** **0.12 (0.02; 0.22)**	**− 0.15 (− 0.25; − 0.05)** **− 0.12 (− 0.22; − 0.03)** **− 0.10 (− 0.19; − 0.01)** **− 0.10 (− 0.19; − 0.01)**	**− 0.15 (− 0.24; − 0.06)** **− 0.13 (− 0.21; − 0.05)** **− 0.14 (− 0.22; − 0.06)** **− 0.11 (− 0.19; − 0.03)**	**0.00** **0.00** **0.00** **0.00**
Wine consumption	1 2 3A 3B	**− 0.11 (− 0.15; − 0.08)** **− 0.10 (− 0.13; − 0.06)** **− 0.09 (− 0.12; − 0.06)** **− 0.08 (− 0.11; − 0.04)**	**0.24 (0.15; 0.33)** **0.15 (0.07; 0.24)** **0.11 (0.03; 0.20)** **0.10 (0.02; 0.18)**	**− 0.20 (− 0.31; − 0.09)** **− 0.17 (− 0.27; − 0.06)** **− 0.16 (− 0.26; − 0.06)** **− 0.15 (− 0.25; − 0.05)**	**− 0.12 (− 0.23; − 0.01)** − 0.06 (− 0.17; 0.04) − 0.07 (− 0.17; 0.03) − 0.05 (− 0.15; 0.05)	**0.00** **0.00** **0.00** **0.00**
Beer consumption	1 2 3A 3B	**0.04 (0.01; 0.08)** − 0.01 (− 0.04; 0.03) − 0.02 (− 0.05; 0.02) − 0.01 (− 0.04; 0.03)	**− 0.08 (− 0.17; − 0.00)** − 0.01 (− 0.10; 0.07) − 0.02 (− 0.10; 0.07) − 0.02 (− 0.10; 0.06)	**− 0.14 (− 0.27; − 0.01)** − 0.11 (− 0.23; 0.01) **− 0.14 (− 0.25; − 0.02)** **− 0.13 (− 0.25; − 0.02)**	0.00 (− 0.15; 0.15) − 0.05 (− 0.19; 0.08) − 0.10 (− 0.23; 0.04) − 0.07 (− 0.20; 0.06)	0.57 0.30 0.09 0.21
Spirits consumption	1 2 3A 3B	**0.17 (0.03; 0.31)** 0.05 (− 0.08; 0.17) − 0.00 (− 0.13; 0.12) − 0.01 (− 0.13; 0.11)	− 0.05 (− 0.13; 0.04) 0.00 (− 0.09; 0.09) − 0.01 (− 0.09; 0.08) − 0.01 (− 0.09; 0.07)	**− 0.24 (− 0.39; − 0.10)** **− 0.15 (− 0.28; − 0.02)** **− 0.17 (− 0.30; − 0.04)** **− 0.16 (− 0.29; − 0.04)**	**− 0.33 (− 0.58; − 0.08)** − 0.18 (− 0.41; 0.05) − 0.19 (− 0.42; 0.03) − 0.17 (− 0.40; 0.05)	**0.01** 0.40 0.83 0.69

Betas and 95% confidence intervals indicate the strength of the association between total alcohol, wine, beer, and spirits consumption with the total MVD composite score where a negative beta indicates less MVD. Total alcohol, wine, beer, and spirits consumption were entered in the models as a continuous variable (per unit, i.e. 10 g/day), as dummies (none, moderate or high versus light alcohol consumption) or (for the P-for trend analyses) as a categorical variable (none, light, moderate, and high alcohol consumption)

One SD corresponds with 1.6 ml white matter hyperintensity volume (logarithmic scale), 2.4 cerebral microbleeds (logarithmic scale), 1.6 lacunar infarcts (logarithmic scale; all three combined in the CSVD features composite score); 20.2 MU of CRAE, 31.4 MU of CRVE (combined in the retinal microvascular diameter composite score); 3.6 MU of flicker light-induced increase in retinal arteriolar diameter, 4.1 MU of flicker light-induced increase in retinal venular diameter (combined in the flicker light-induced increase in retinal microvascular diameter composite score); 0.98 mg/24 h of logarithmically transformed UAE; 57.3 PU of heat-induced skin hyperemia; or 99.8 ng/ml sICAM-1, 101.0 ng/ml of sVCAM-1, 65.7 ng/ml of sE-selectin, or 48.4% vWF (combined in the plasma biomarkers of MVD composite score)

The numbers of participants with complete data on CSVD features, retinal microvascular diameters, flicker light-induced increase in retinal microvascular diameters, heat-induced skin hyperemia, UAE, and plasma biomarkers of MVD respectively are n = 2075; n = 2721; n = 2090; n = 1,517; n = 3107; and n = 3078

Model 1: crude; Model 2: age, sex, glucose metabolism status (entered as dummies of type 2 diabetes, prediabetes, or other types of diabetes versus normal glucose metabolism status), education level [low, middle, high]; model 3A: model 2 + waist circumference, smoking status [current, ever, never], diet score; model 3B: model 3A + office systolic blood pressure, use of antihypertensive medication [yes/no] total cholesterol/HDL cholesterol ratio, lipid-modifying medication, prior cardiovascular disease. Additionally, for associations with heat-induced skin hyperemia baseline skin blood flow was entered in model 1

Bold denotes P-value < 0.05

*CI* confidence interval, *CSVD* cerebral small vessel disease, *CRAE* central retina arteriolar equivalent, *CRVE* central retinal venular equivalent, *SD* standard deviation, *PU* perfusion units, *UAE* urinary albumin excretion, *sICAM-1* soluble intercellular adhesion molecule-1, *sVCAM-1* soluble vascular adhesion molecule-1, *sE-selectin* soluble E-selectin, *vWF* von Willebrand factor, *MVD* microvascular dysfunction

### Interaction analyses

History of cardiovascular disease and sex modified the association between total alcohol consumption and the total MVD composite score (P-for-interaction values: < 0.001 and = 0.03, respectively). Additional file 1: Table S3 shows all P-for-interaction values.

### Stratified analyses

#### History of cardiovascular disease

In individuals with and without a history of cardiovascular disease the shapes of the non-linear association of total alcohol consumption with the total MVD composite score were different, both with regard to the location of the minimum of the J-curve as well as the depth of the minimum of the J-curve. The minimum of the J-curve, was at approximately 6 units/day in the crude model in individuals with a history of cardiovascular disease; and at approximately 2 units/day in the crude model in individuals without a history of cardiovascular disease (Additional file 1: Figure S1). Then, the minimum of the J-curve was lower in individuals with, versus without, a history of cardiovascular disease (indicating that higher than light total alcohol consumption was more strongly associated with less MVD in individuals with, versus without, a history of cardiovascular disease; e.g. model 3B; for moderate versus light total alcohol consumption, -0.22 [-0.44; -0.01] in individuals with a history of cardiovascular disease versus -0.09 [-0.19; 0.01] in individuals without a history of cardiovascular disease; Fig. 3 and Additional file 1: Table S4). Next, in individuals with a history of cardiovascular disease, wine and beer consumption were more strongly associated with less MVD than spirits consumption (model 3B; for moderate versus light wine, beer, and spirits consumption, respectively, -0.29 [-0.56; -0.03]; -0.28 [-0.55; 0.00]; and -0.21 [-0.51; 0.10]; Additional file 1: Table S4 and S Additional file 1: Figure S2).

#### Sex

In men and women, the shapes of the associations of total alcohol consumption with the total MVD composite score clearly differed with regard to the location of the minimum of the J-curve and somewhat, but not materially, differed with regard to the depth of the minimum of the J-curve. The minimum of the J-curve for total alcohol consumption in the association with the total MVD composite score was at approximately 5 units/day in men in the crude model; and at approximately 3 units per/day in women in the crude model (Additional file 1: Figure S1). Then, the strength of this association was somewhat stronger in women than in men (e.g. model 3B; moderate versus light total alcohol consumption, -0.11 [-0.25; 0.04] in women versus − 0.09 [− 0.20; 0.03] in men; Fig. 3 and Additional file 1: Table S5). Additionally, in both men and women, wine consumption was somewhat more strongly associated with less MVD, estimated from the total MVD composite score, than beer or spirits consumption, where wine, but not beer or spirits, consumption was somewhat more strongly associated with less MVD in women than in men (model 3B; moderate versus light total alcohol consumption, − 0.09 [− 0.14; − 0.01] in women versus -0.06 [-0.11; -0.02] in men; Additional file 1: Table S5).

#### Additional analyses

We observed quantitatively similar results in a range of additional analyses (all results are reported in the Extended Results section in the Additional file 1: Tables S6-S18 and Additional file 1: Figures S1-S14). We highlight three findings. First, we found that alcohol consumption was in the same direction associated with retinal arteriolar and venular diameters (model 3B; Additional file 1: Table S9). Second, we found that the minimum in the J-curve was at increasingly higher levels of total alcohol consumption in individuals with increasingly more cardiovascular risk factors (Additional file 1: Table S7 and Additional file 1: Figure S5). Third, when we left wine or beer consumption out of the total alcohol consumption index the location of the minimum of the J-curve was different (at higher levels of alcohol consumption when wine was left out of the index and at lower levels of alcohol consumption when beer was left out of the index; Additional file 1: Figure S8). We did not see material changes when we left spirit out of the index (Additional file 1: Figure S8).

## Discussion

The present population-based study has three main findings. First, in the complete population we found a J-shaped association between total alcohol consumption with MVD, indicating that moderate versus light total alcohol consumption was associated with less MVD and higher than moderate versus light total alcohol consumption was associated with more MVD. In addition, associations with MVD were similar for wine, beer, and spirits. Second, in individuals with, versus without, a history of the cardiovascular disease, the minimum of the J-curve was at higher levels of total alcohol consumption; and the depth of the minimum of the J-curve was considerably lower. In addition, in individuals with a history of cardiovascular disease, the depth of the minimum of the J-curve was considerably lower for wine and beer consumption than for spirits consumption. Third, in men, the minimum of the J-curve was at higher levels of alcohol consumption than in women; and in women the depth of the minimum of the J-curve was somewhat lower than in men (indicating a somewhat stronger association in women than in men). In addition, wine consumption was somewhat more strongly associated with less MVD in women than in men. We did, however, not see a consistent pattern for other types of alcoholic beverages (i.e. beer and spirits).

Our findings are in line with observations from most previous studies [15–53]. Importantly, the present study is the first large population-based study to comprehensively report associations of total alcohol, wine, beer, and spirits consumption with MVD assessed in various organs, both in the general population as well as in substrata of individuals with a history of cardiovascular disease or a cardiovascular risk factor. Further, the present study is the first study to report the associations of total alcohol, wine, beer, and spirits consumption with flicker light-induced increase in retinal diameters and heat-induced skin hyperemia.

Our observations support the concept that alcohol consumption is a determinant of MVD. All measures of MVD under study likely (in part) reflect endothelial cell function, which relies on NO bioavailability, and NO bioavailability can be modified by alcohol consumption [2, 6, 7]. Mechanistically, the J-shaped association between alcohol and MVD may reflect a triphasic balance, where in the descending part of the curve alcohol consumption induces a net increase in NO bioavailability (reducing MVD); at the minimum of the curve there is an equilibrium between increasing and reducing effects of alcohol on NO bioavailability (net no effect on MVD); and in the ascending part of the curve, alcohol consumption induces a net reduction in NO bioavailability (increasing MVD) [5, 9, 74–76]. Biologically, at lower levels of alcohol consumption ethanol likely increases NO bioavailability via stimulation of NO synthesis by the enzyme eNOS; [5, 9, 74–76] and polyphenols likely increase NO bioavailability via reducing eNOS uncoupling and scavenging of NO by oxidative stress [5, 9, 74–76]. Then, at higher levels of alcohol consumption ethanol likely reduces NO bioavailability by inducing oxidative stress [5, 9, 74–76].

In individuals with, versus without, a history of the cardiovascular disease, the minimum of the J-curve in the association of total alcohol consumption with the total MVD composite score was located at higher levels of total alcohol consumption, possibly because levels of background oxidative stress are higher in individuals with, versus without, a history of cardiovascular disease [5, 74]. Biologically, higher levels of ethanol-induced oxidative stress may be required to induce more oxidative stress than already present in the background [5, 27]. Indeed, consistent with this concept, we found that the minimum in the J-curve was located at higher levels of alcohol consumption in individuals with, versus without, a cardiovascular risk factor (for any individual risk factor under study).

In individuals with, versus without, a history of the cardiovascular disease the depth of the minimum of the J-shaped association of total alcohol consumption with MVD was considerably lower (indicating a stronger association of total alcohol consumption with MVD), possibly because at higher, versus lower, levels of background oxidative stress polyphenols can more potently increase NO bioavailability [5, 5, 27, 74]. Biologically, polyphenols can both increase NO bioavailability via preventing the scavenging of co-factors that are required for NO synthesis and via inhibiting a vicious circle in which oxidative stress scavenges NO and oxidizes NO into more oxidative stress (i.e. peroxynitrate, a reactive nitrogen species) [5, 27]. Indeed, consistent with this concept, in individuals with a history of cardiovascular disease, wine and beer consumption, which reflect greater intake of polyphenols than spirits consumption, were more strongly associated with less MVD than spirits consumption.

In men, versus women, the minimum of the J-curve was located at higher levels of total alcohol consumption, likely due to sex differences in the pharmacokinetics of ethanol [77, 78]. Biologically, as in women, versus men, the gastric activity of the antidiuretic hormone (ADH) is lower, which regulates the clearance of ethanol (first-pass metabolism), the consumption of a comparable quantity of ethanol likely leads to a higher level of ethanol in the blood of women than men [77, 78]. Additionally, as women on average have a lower volume of body water than men and ethanol is distributed in water in the body, the consumption of a comparable quantity of ethanol likely leads to higher blood concentrations of ethanol in women than in men [77, 78].

In women the depth of the minimum of the J-curve was somewhat, but not materially, lower than in men, possibly because certain small polyphenol-based pharmacodynamic sex differences exist [14, 79]. Biologically, as certain polyphenols in alcoholic beverages (e.g. resveratrol) can, via binding to the estrogen receptor, in a sex-specific manner alter intra-endothelial cell signaling pathways that regulate NO bioavailability, alcohol consumption may more strongly lead to an increase in endothelial cell NO bioavailability in women than in men [14, 79, 80]. Indeed, consistent with this concept, we found that wine consumption, which reflects greater resveratrol intake (mainly from red wine), was somewhat more strongly associated with less MVD in women than in men [14, 79].

In analyses with individual measures of MVD we observed that higher alcohol consumption was associated with narrower retinal microvascular diameters. Retinal arteriolar widening is thought to occur in early stages of MVD; thus, a narrower arteriolar diameter may represent less widening (i.e. indicating less MVD) [2, 4]. Biologically, widening of retinal arteriolar diameter is thought to reflect impairment of autoregulation, which is (in part) thought to be caused by endothelial cell dysfunction, as well as focal downstream ischemia [2, 4]. Indeed, human and animal data from observational and experimental studies in the retina, as well as in other organs such as the kidney, support this concept [2, 4].

Our findings should not be interpreted as to imply that changing alcohol consumption can be used to prevent MVD. Every unit increase in consumption of alcohol is associated with increased risk of loss of disability-adjusted life-years, as found in The Global Burden of Alcohol study which used data from 195 countries [81]. In addition, another important point is that it remains under debate which threshold for alcohol consumption should be recommended. Previous studies found differing thresholds at which alcohol consumption was associated with more favorable health outcomes. For example, a recent individual participant data analysis of n ~ 600,000 participants found that 100 g/week of alcohol consumption (for both men and women) was associated with a lower risk of all-cause mortality; [82] and a recent randomized clinical trial found that < 1 unit of alcohol consumption was associated with a decrease in arterial stiffness [83]. These results differ from our results, in which we found that up to two units per day of alcohol consumption for women and up to five units per day or alcohol consumption for men were associated with less MVD. Nevertheless, our findings add to the increasing body of evidence that it may be possible to reduce MVD via dietary interventions; and that it may be possible to personalize recommendations on alcohol consumption according to the presence of risk factors for cardiovascular disease [84].

Main strengths of this study are the large size of this population-based cohort study with oversampling of individuals with type 2 diabetes, which enables accurate comparison of individuals with and without diabetes [73]; the large number of potential confounders that was considered [85]; and the use of state-of-the-art techniques to assess CSVD features and MVD in various organ beds [60]. In addition, a strength of this study is that sick quitters were accounted for in analyses in which light alcohol consumption was used as a reference group [55].

Limitations include the following. First, due to the cross-sectional nature of the study causal inferences should be made with considerable caution. Second, some misclassification of high drinkers may have occurred as high drinkers may be more likely to self-underreport their alcohol consumption [36]. This may have led to an underestimation of strength of the associations in this study [71]. Third, even though we took an extensive set of confounders into account, we cannot fully exclude unmeasured confounding. For example, we did not take binge drinking into account and binge drinking may be more detrimental than chronic high alcohol consumption [6]. Fourth, there were relatively low numbers of high beer consumers (≤ 7% of participants) and moderate or high spirits consumers (≤ 2% of participants) in this study and this may resulted in a lack of statistical power to be able to detect statistically significant associations of beer and spirits consumption with endpoints under study (i.e. type 2 error) [73]. Fifth, we could not account for how drinking behavior changes related to the presence of certain medical conditions may have affected the analyses. For example, certain individuals with type 2 diabetes may have quit alcohol consumption due to dietary restrictions (imposed by their medical doctors due to the presence of cardiovascular risk factors). Sixth, we studied Caucasian individuals aged 40–75 years and therefore our results may be generalizable to such a population; whether these results also apply to other populations requires further study [86].

In conclusion, in this cross-sectional study we found a J-shaped association between total alcohol, wine, beer, and spirits consumption and MVD, indicating that moderate versus light alcohol consumption was associated with less MVD and higher than moderate versus light alcohol consumption was associated with more MVD. Additionally, the location and the depth of the minimum of the J-curve differed by history of cardiovascular disease and sex. Therefore, alcohol consumption may have an effect on MVD and via MVD mitigate microvascular clinical disease such as stroke, dementia, depression, retinopathy, and chronic kidney disease [1–3]. Although increasing alcohol consumption cannot be recommended as a policy, this study suggests that prevention of MVD may be possible through dietary interventions.

## Supplementary Information

**Additional file 1.**

## Data Availability

Data are available from The Maastricht Study for any researcher who meets the criteria for access to confidential data; the corresponding author may be contacted to request data. Data described in the manuscript, code book, and analytic code will be made available upon request pending (e.g., application and approval, payment, other).

## Abbreviations

MVD: Microvascular dysfunction
CSVD: Cerebral small vessel disease
UAE: Urinary albumin excretion
sICAM-1: Soluble intercellular adhesion molecule-1
sVCAM-1: Soluble vascular adhesion molecule-1
sE-selectin: Soluble E-selectin
vWF: Von Willebrand Factor
NO: Nitric oxide
eNOS: Endothelial cell nitric oxide synthase
CRAE: Central retinal arteriolar equivalent
CRVE: Central retinal venular equivalent
CKD: Chronic kidney disease
PU: Perfusion units
MU: Measurement units
