# Supplementary material for: Alcohol consumption and microvascular dysfunction: a J-shaped association: The Maastricht Study
Source: Cardiovasc Diabetol. 2023 Mar 24;22:67. doi: 10.1186/s12933-023-01783-x (PMC10039613; doi:10.1186/s12933-023-01783-x)
Supplement: Supplementary file 1 — Additional file 1. [file 12933_2023_1783_MOESM1_ESM.doc]

**SUPPLEMENTAL MATERIAL**

***Contents***

Supplemental Methods

Supplemental Results

Supplemental Figures

- Supplemental Figure S1 Scatter plots with quadratic regression line modeling, in individuals with and without a history of cardiovascular disease and in men and women
- Supplemental Figure S2 Associations of total alcohol, wine, beer, and spirits consumption (per unit) with measures of MVD (in SD; model 3B), in the general population and stratified by history of cardiovascular disease
- Supplemental Figure S3 Associations of total alcohol consumption (per unit) with measures of MVD (in SD; model 3B), in the general population and stratified by history of cardiovascular disease or cardiovascular risk factor status
- Supplemental Figure S4 Associations of wine, beer, and spirits consumption (per unit) with measures of MVD (in SD; model 3B), stratified by cardiovascular risk factor status
- Supplemental Figure S5 Associations of total alcohol consumption with the total MVD composite score, stratified by number of cardiovascular risks or history of cardiovascular disease
- Supplemental Figure S6 Scatter plots with quadratic regression line modeling, stratified by cardiovascular risk factor status
- Supplemental Figure S7 Scatter plot with a third degree polynomial function in the complete study population
- Supplemental Figure S8 Scatter plots with quadratic regression line modeling, where wine, beer, or spirits were left out of the total alcohol consumption index
- Supplemental Figure S9 Associations of total alcohol consumption (per unit) with measures of MVD (in SD; model 3B), in the general population
- Supplemental Figure S10 Associations of total alcohol consumption (per unit) with measures of MVD (in SD; model 3B), stratified by history of cardiovascular disease status
- Supplemental Figure S11 Associations of total alcohol consumption (per unit) with measures of MVD (in SD; model 3B), stratified by glucose metabolism status
- Supplemental Figure S12 Associations of total alcohol consumption (per unit) with measures of MVD (in SD; model 3B), stratified by hypertension status
- Supplemental Figure S13 Associations of total alcohol consumption (per unit) with measures of MVD (in SD; model 3B), stratified by dyslipidemia status
- Supplemental Figure S14 Associations of total alcohol consumption (high versus light) with measures of MVD (in SD; model 3B), stratified by current smoking status

Supplemental Tables

- Supplemental Table S1 Characteristics of the study population and individuals excluded from the analyses due to missing values for the CSVD features, retinal microvascular diameters, and flicker light-induced increase in retinal microvascular diameters study populations
- Supplemental Table S2 Characteristics of the study population and individuals excluded from the analyses due to missing values for the heat-induced skin hyperemia, UAE, and plasma biomarkers of MVD study populations
- Supplemental Table S3 P-values from likelihood ratio tests for interaction
- Supplemental Table S4 Associations of total alcohol, wine, beer, and spirits consumption with the total MVD composite score, stratified by history of cardiovascular disease status
- Supplemental Table S5 Associations of total alcohol, wine, beer, and spirits consumption with the total MVD composite score, stratified by sex
- Supplemental Table S6 Associations of total alcohol, wine, beer, and spirits consumption with the total MVD composite score, stratified by cardiovascular risk factor status
- Supplemental Table S7 Associations of total alcohol consumption with the total MVD composite score, stratified by number of cardiovascular risk factors
- Supplemental Table S8 Associations of total alcohol consumption with the total MVD composite score composed out of five instead of six measures of MVD
- Supplemental Table S9 Associations of total alcohol consumption with measures of MVD, in the general population
- Supplemental Table S10 Associations of total alcohol consumption with individual measures used in composite scores, in the general population
- Supplemental Table S11 Associations of wine, beer and spirits consumption with MVD measures in the general population and stratified by history of cardiovascular disease status
- Supplemental Table S12 Associations of total alcohol consumption with MVD measures, stratified by history of cardiovascular disease status
- Supplemental Table S13 Associations of total alcohol consumption with MVD measures, stratified by glucose metabolism status
- Supplemental Table S14 Associations of total alcohol consumption with MVD measures, stratified by presence of hypertension status
- Supplemental Table S15 Associations of total alcohol consumption with MVD measures, stratified by dyslipidemia status
- Supplemental Table S16 Associations of total alcohol consumption with MVD measures, stratified by current smoking status
- Supplemental Table S17 Associations of total alcohol consumption with the total MVD composite score and MVD measures, additionally adjusted for physical activity, diabetic retinopathy, eGFR, and plasma biomarkers of low-grade inflammation
- Supplemental Table S18 Associations of total alcohol consumption with CSVD features, retinal microvascular diameters, and measures of MVD, where educational level was replaced with income level (model 3C) or occupational status (model 3D); glucose metabolism status was replaced with fasting plasma glucose (model 3E), or 2-hour post load (model 3F) or HbA1c (model 3G); or office systolic blood pressure was replaced with office diastolic blood pressure (model 3H), 24-hour ambulatory systolic blood pressure (model 3I) or 24-hour ambulatory diastolic blood pressure (model 3J)

**Supplemental Methods**

**Assessment of cerebral small vessel disease (CSVD) features, retinal microvascular diameters, and microvascular dysfunction (MVD) measures**

Participants were asked to refrain from smoking and drinking caffeine-containing beverages three hours before the measurement.^1^ A light meal was allowed until ≥90 minutes prior to the examination. ​For retinal measurements, pupils were dilated with 0.5% tropicamide and 2.5% phenylephrine ≥15 minutes before the start of the examination. Skin blood flow measurements were performed in a climate-controlled room at 24 °C.^1^

*Brain magnetic resonance imaging*

We evaluated three CSVD features, i.e. white matter hyperintensity volume, lacunar infarcts and cerebral microbleeds with a 3T brain magnetic resonance imaging (MRI) scanner (Siemens Magnetom Prisma-fit Syngo MR D13D, Erlangen, Germany) and used a 64-element head/neck coil for parallel imaging. The MRI protocol consisted of a 3D T_1_-weighted magnetization prepared rapid acquisition gradient echo (MPRAGE) sequence (TR/TI/TE 2300/900/2.98 ms, 176 slices, 256×240 matrix size, 1.00 mm cubic voxel size); a fluid-attenuated inversion recovery (FLAIR) sequence (TR/TI/TE 5000/1800/394 ms, 176 slices, 512×512 matrix size, 0.49×0.49×1.00 mm voxel size); a combined proton density (PD) and T_2_-weighted turbo spin echo (TSE) pulse sequence (TR/TE1/TE2 3200/9.4/94 ms, 30 slices, 640×540 matrix size, 0.36×0.36×4.00 mm voxel size); and a susceptibility-weighted imaging (SWI) sequence (TR/TE 28/20 ms, 144 slices, 384×312 matrix size, 0.57×0.57×1.00 mm voxel size).

Contra-indications for MRI assessment were presence of: a cardiac pacemaker or implantable cardioverter-defibrillator, a neurostimulator, a non-detachable insulin pump, metallic vascular clips or stents in the head, a cochlear implant, a metal-containing intra-uterine device, metal splinters or shrapnel, dentures with magnetic clip, an inside bracket, pregnancy, epilepsy, or claustrophobia.

T_1_-weighted images and FLAIR images were analyzed with an ISO-13485:2012 certified automated method (which included visual inspection).^2, 3^ T_1_-weighted images were segmented into grey matter, white matter and cerebrospinal fluid volumes (1 voxel = 1.00 mm^3^ = 0.001 ml).^2^ Intracranial volume was calculated as the sum of grey matter, white matter (including white matter hyperintensity volume), and cerebrospinal fluid volumes. White matter hyperintensity volume was summed to assess total white matter hyperintensity burden, and expressed relative to intracranial volume. Lacunar infarcts were defined as focal brain parenchyma defects of ≥3 mm and <15 mm in size with a similar signal intensity as cerebrospinal fluid on all sequences and a hyperintense rim on T_2_ and FLAIR images.^4^ Cerebral microbleeds were rated on T_2_-weighted and SWI images by use of the Microbleed Anatomical Rating Scale,^5^ and were defined as focal lesions of ≥2 mm and ≤10 mm in size with a hypointense signal.^4^ The number of lacunar infarcts and cerebral microbleeds was rated manually by three neuroradiologists. The two-way mixed effects, consistency, intraclass correlation coefficients for the presence of lacunar infarcts and cerebral microbleeds for the three raters based on 50 randomly selected scans were 0.84 (95% confidence interval 0.74; 0.91) and 0.83 (0.72; 0.90), respectively.

*Retinal microvascular diameters*

We measured retinal arteriolar and venular diameters with static retinal vessel analysis from an optic disk-centered fundus photograph. All fundus photographs were taken with an auto-focus, auto-shot, and auto-tracking fundus camera (Model AFC-230; Nidek, Gamagori, Japan) in an optic disc-centered field of view of 45° in a darkened room. We manually evaluated quality of fundus photographs and excluded images of insufficient quality (e.g. images in which lashes obstructed the view on the optic nerve head or images that were defocused). Per participant we randomly selected one fundus photo from one eye (either left or right eye) to calculate retinal vessel diameters. We used the retinal health information and notification system (RHINO) software developed by the RetinaCheck group of the Technical University of Eindhoven (Eindhoven, the Netherlands) to assess the diameters of the six largest retinal vessels at 0.5–1.0-disc diameter away from the optic disc margin (expressed in measurement units [MU]).^6, 7^ We manually checked the automatically detected optic disc position and arteriole/venule classification and adjusted if necessary. Diameters of arteriolar or venular vessels were combined into an average arteriolar diameter (i.e. central retinal arteriolar equivalent [CRAE]) or venular diameter (i.e. central retinal venular equivalent [CRVE]).The scale factor, based on the optic disc diameter, was assumed to be 1800 μm,^8^ i.e. 1 MU = 1 pixel size× 1800 μm/pixel size of optic disc diameter. The calculations were based on the improved Knudtson–Parr–Hubbard formula. ^9^ We calculated the intraclass correlation coefficients for CRAE and CRVE to assess the agreement between analyses of the RHINO software with vs without manual identification of arterioles and venules using 2556 images. The intraclass correlation coefficients for CRAE and CRVE respectively were 0.910 and 0.897.

*Retinal flicker light-induced increase in retinal arteriolar and venular diameters*

The flicker light-induced increase in retinal arteriolar and venular diameters, which is thought to be related to nutritive demands of activated retinal neurons,^10^ was measured in a dimly lit room with the Dynamic Vessel Analyzer (DVA) (Imedos Systems GmbH, Jena, Germany). For safety reasons, participants with an intraocular pressure exceeding 30 mmHg were excluded from retinal measurements. Per participant, we randomly measured the left or right eye.

During the measurement, the participant was instructed and encouraged to focus on the tip of a fixated needle inside the retinal camera (FF450; Carl Zeiss GmbH, Jena, Germany) while the fundus of the eye was examined under green measuring light (530-600 nm, illumination of fundus approximately 6500 lux). A straight arteriolar or venular segment of approximately 1.5 mm in length located 0.5 to 2.0-disc diameter from the margin of the optic disc in the temporal section was examined. When the specific vessel profile was recognized the vessel diameter was automatically and continuously measured for 150 seconds. A baseline recording of 50 seconds was followed by a 40-second flicker-light exposure period (flicker frequency 12.5Hz, bright-to-dark contrast ratio 25:1) followed by a 60-second recovery period. The DVA automatically corrected for alterations in luminance caused by e.g. slight eye movements. During blinks and small eye movements the registration stopped and restarted once the vessel segments were automatically re-identified.^10^

The integrated DVA software (version 4.51, Imedos) automatically calculated the baseline arteriolar and venular diameter and the increase in arteriolar and venular diameters during flicker light exposure. Baseline diameter was calculated as the average diameter during the 20-50 seconds baseline recording and was expressed in MU, where 1 MU is equal to 1µm of the Gullstrand eye.^11^ Flicker light-induced increase in retinal microvascular diameter was calculated as the diameter during flicker light exposure minus the baseline diameter. The diameter during flicker light exposure was defined as the mean of the diameter assessed at time points 10 and 40 seconds of flicker light stimulation exposure. The purpose of taking the average increase in diameter at two time points during flicker light stimulation was to account for the inter-individual variation in increase in diameter during exposure to flicker light. For 95.4% of the measurements the assessment of flicker light-induced increase in arteriolar and venular diameter was based on the arteriolar and venular diameters assessed at both 10 and 40 seconds of flicker light stimulation. For 4.6% of the measurements the assessment of flicker light-induced increase in arteriolar and venular diameters was based on the arteriolar and venular diameter assessed at either 10 or 40 seconds of flicker light stimulation.

The measurement of retinal flicker light-induced increase in arteriolar and venular diameters was done by different trained observers. The inter‐observer reliability intraclass coefficients for the assessment of baseline retinal microvascular diameter and flicker-light induced increase in retinal microvascular diameter between two randomly selected observers were, respectively, 0.980 and 0.796 for arteriolar vessels and 0.972 and 0.871 for venular vessels (N=9 participants).^1^ The quality of the flicker light-induced increase in retinal arteriolar and venular response curves was analyzed by one observer. Retinal response curves of insufficient measurement quality, e.g. insufficient measurement points or movement artifacts, were evaluated and discussed with a second observer and excluded on mutual agreement. To assess the inter‐observer reliability of retinal response curves quality decisions, 50 curves were evaluated by two observers (inter‐observer reliability =0.883).^1^

*Heat-induced skin hyperemia*

Skin blood flow was measured with a laser-Doppler system (Periflux 5000; Perimed, Järfalla, Sweden) equipped with a thermostatic laser-Doppler probe (PF457; Perimed) at the dorsal side of the wrist of the left hand. The laser-Doppler output was recorded for 25 minutes with a sample rate of 32 Hz, which gives semiquantitative assessment of skin blood flow expressed in arbitrary perfusion units. Skin blood flow at the wrist, expressed in arbitrary perfusion units (PU), was recorded unheated for 2 minutes to serve as a baseline. After 2 minutes, the temperature of the laser Doppler probe was rapidly and locally increased to 44°C and was kept constant until the end of the registration. In 596 individuals, the recording of skin blood flow was incomplete between 20 and 25 minutes of the recording. For these individuals data were extrapolated to 25 minutes using the last-minute average as reference, with a weighted correction factor of 1.017. This correction factor is the ratio of the increase in average PU in the 20-25-minute interval as compared to the average PU in the 19-20-minute interval.

For analyses where heat-induced skin hyperemia was included in the total MVD composite score, we expressed heat-induced increase in skin blood as the increase in heat-induced increase in skin blood flow during the 23 minutes heating phase. We calculated heat-induced increase in skin blood flow as the average skin blood flow during the 23 minutes heating phase minus the baseline skin blood flow (i.e. average skin blood flow during the first 2 minutes). For other analyses, we expressed heat-induced increase in skin blood flow as the average skin blood flow during the 23 minutes heating phase and adjusted for the baseline skin blood flow to account for inter-individual differences in baseline skin blood flow, as previously described.^12^ We checked whether both approaches yielded the same results and this was the case (data not shown).

*Urinary albumin excretion*

Urinary albumin excretion (UAE) was calculated as the average UAE of two 24-hour urine collections (two collections were available for 91.3% of participants). We measured urinary albumin concentration with a standard immunoturbidimetric assay by an automatic analyzer (due to a change of supplier, by the Beckman Synchron LX20 and the Roche Cobas 6000). We multiplied urinary albumin concentration by collection volume to obtain 24-hour UAE. A urinary albumin concentration below the detection limit of the assay was set at 1.5 mg/L (2 mg/L for the Beckman Synchron LX20 and 3 mg/L for the Roche Cobas 6000) before multiplying by collection volume. Only urine collections with a collection time between 20 and 28 hours were considered valid. If needed, UAE was extrapolated to 24-hour excretion.

*Plasma biomarkers of microvascular dysfunction*

Four plasma biomarkers of MVD were evaluated (soluble intercellular adhesion molecule-1 [sICAM-1], soluble vascular adhesion molecule-1 [sVCAM-1], soluble E-selectin [sE-selectin] and Von Willebrand Factor [vWF]). sICAM-1, sVCAM-1 and sEselectin of the first 866 individuals of The Maastricht Study were measured in ethylenediaminetetraacetic acid (EDTA) plasma samples with commercially available 4-plex sandwich immunoassay kits (Meso Scale Discovery (MSD), Rockville, Maryland, United States of America) as described previously.^13^ From individual 867 onwards, plasma biomarkers were measured in EDTA plasma samples with renewed commercially available 4-plex sandwich immunoassay kits with different standards and antibodies (Meso Scale Discovery (MSD), Rockville, Maryland, United States of America). For this technique in this study, the intra- and inter-assay coefficients of variation were respectively 10.3% and 8.4% for sICAM-1, 5.0% and 4.7% for sVCAM-1, and 2.9% and 7.4% for sE-selectin. Absolute values of plasma biomarkers differed between the individuals measured with the old and renewed 4-plex sandwich immunoassay kits. To realign the absolute values of individuals measured with the old 4-plex sandwich immunoassay to individuals measured with the renewed 4-plex sandwich immunoassay, realign formulas were calculated with Deeming regression analyses.^14^ In order to do so, the first 419 out of 866 individuals, who were measured with the old 4-plex sandwich immunoassay, were measured with the renewed 4-plex sandwich immunoassay as well. The biomarker of endothelial dysfunction vWF was quantified in citrate plasma using ELISA (Dako, Glostrup, Denmark). The intra- and inter-assay coefficients of variation were 3.0% and 4.3%, respectively.

**Covariates**

Questionnaires were used to assess education level, occupational level, and smoking. Educational level was assessed from a questionnaire in which participants were asked to indicate their highest level of education. Categories of educational level were computed as follows: low = no education, (un)completed primary education, or lower vocational education; intermediate = intermediate vocational education or higher secondary education; high = higher vocational education or university education. Next, occupational status groups (low, intermediate, high) were composed based on tertiles of the International Socio‑Economic Index 2008 of Occupational Status. Smoking status was assessed from participants responses to a questionnaire in which they were asked they were current or former smokers, or never smoked.

**Statistical analyses**

*Calculation of the MVD composite score*

We used a total MVD composite score as endpoint. We composed a total MVD composite score because we assume that all measures of MVD under study represent a similar underlying measure of MVD.^15^ In order to perform analyses we recalculated several variables. First, we inversed (i.e. multiplied by -1) flicker light-induced increase in retinal arteriolar and venular diameters and heat-induced skin hyperemia so that higher values indicate more MVD. Second, we logarithmically transformed white matter hyperintensity volume, cerebral microbleeds, lacunar infarcts, and UAE as these outcome variables were not normally distributed. Third, to reduce noise (i.e. measurement error) we calculated composite scores for CSVD features, retinal microvascular diameters, flicker light-induced increase in retinal microvascular diameters, plasma biomarkers of MVD, and total MVD.^16^ Before we computed the total MVD composite score, we checked whether associations of alcohol consumption with individual outcome variables were directionally consistent and this was the case for all MVD measures (including for retinal arteriolar and venular diameters). To calculate the composite score, we standardized all MVD measures (i.e. expressed as z-score) and summarized these z-scores into a total MVD composite z-score. We composed the total MVD composite z-score in two steps. To start, we combined z-scores for individual CSVD features, retinal arteriolar and venular diameters, flicker light-induced increase in retinal arteriolar and venular diameters, and individual plasma biomarkers of MVD into composite z-scores. Next, we computed the total MVD composite score by averaging the composite z-scores for CSVD features, retinal microvascular diameters, flicker light-induced increase in retinal microvascular diameters, and plasma biomarkers of MVD and the z-scores for heat-induced skin hyperemia and UAE. We then again standardized the composite score to ensure that the total MVD z-score was correctly calculated (if the composite score is not standardized again, the composite score may be somewhat spuriously estimated due to small rounding errors as a consequence of the averaging of z-scores [which themselves are rounded numbers]). To maximize the number of participants that we could use in the main analyses, we included participants in the main analyses if data were available for at least two out of six measures of MVD. Then, we performed complete cases analyses, i.e. we included individuals in the main analyses if they had complete data on the total MVD composite score, alcohol consumption, and covariates required for the main statistical models (shown below). Last, we recalculated the Dutch Healthy Diet score so that the “diet score” reflects dietary intake without alcohol consumption.

*Other*

Collinearity diagnostics (i.e. tolerance <0.10 and/or variance inflation factor >10) were used to detect multicollinearity between covariates.

*Additional analyses*

To test robustness of our observations we performed several additional analyses. First, we tested whether the association of total alcohol consumption with the total MVD composite score was modified by individual cardiovascular risk factors (i.e. glucose metabolism status, hypertension, current smoking, and dyslipidemia). To test whether this association was modified by individual cardiovascular risk factors, we tested for interaction by these factors. For tests of interaction by glucose metabolism status, we excluded individuals with other types of diabetes than type 2 diabetes because the number of participants with other types of diabetes was small. We defined hypertension, smoking, and dyslipidemia as follows: presence of hypertension was defined as office systolic blood pressure ≥ 140 mm Hg and/or use of antihypertensive medication; current smokers were defined as smokers and both former and never smokers were considered non-smokers; and presence of dyslipidemia was defined as LDL> 3.5 mmol/L (i.e. median LDL level in the general population) and/or use of lipid-modifying medication. Second, we investigated the association between total alcohol consumption and the total MVD composite score in individuals with zero, one, two, three, or four of the above cardiovascular risk factors to investigate whether associations were stronger in individuals with presumed increasingly higher levels of oxidative stress. Third, we investigated whether the association of alcohol consumption with the total MVD composite score was better described by a third, fourth, or fifth order polynomial function than a polynomial function of a first order (i.e. a linear function). To investigate this, we analyzed the association using the following formulas: y=x + x^3^; y=x + x^4^; and y=x + x^5^; and we calculated the P-values of the third (x^3^), fourth (x^4^), or fifth (x^5^) order terms of the polynomial functions. If the P-value of any factor was statistically significant (P-value<0.05) we considered the association better described by a higher order polynomial function than a first degree polynomial (i.e. linear) function. Fourth, we calculated at which amount of alcohol consumption the minimum of the J-curve was located (in the association of total alcohol consumption with the total MVD composite score) after full adjustment (model 3B). Fifth, we investigated how the shape of the association was impacted when we left either wine, beer, or spirits out of the total alcohol consumption index. Sixth, we studied the association between total alcohol consumption and a total MVD composite score based on five instead of six measures of MVD, where we, one by one, left out one of measures of MVD. Seventh, we investigated the associations of total alcohol, wine, beer, and spirits consumption with individual measures used in the total MVD composite scores as endpoint. Additionally, in analyses with heat-induced skin hyperemia as a separate outcome, we entered baseline skin blood flow in model 1 (more details are presented in the Supplemental Methods section). Eighth, we additionally adjusted for accelerometer-assessed physical activity. We did not include accelerometer-assessed physical activity in the main analyses because data was missing for a relatively large number of participants (up to n=699 had missing data).^12^ Nineth, we repeated analyses with additional adjustment for diabetic retinopathy, eGFR, or plasma biomarkers of low-grade inflammation. We entered these covariates in a separate model instead of in model 3B because up to n=497 participants had missing data on one or more of these variables. Tenth, if associations under study were modified by glucose metabolism status, we tested whether interaction terms composed of continuous glycemic measures (i.e. fasting plasma glucose, 2-hour post load glucose, and HbA1c) instead of glucose metabolism status also modified these associations (e.g. HbA1c*total alcohol consumption). Last, we repeated analyses after replacement of educational level with income level and occupational status; after replacement of glucose metabolism status with fasting plasma glucose, 2-hour post load glucose, or HbA1c; and after replacement of office systolic blood pressure with office diastolic blood pressure, 24-hour ambulatory systolic blood pressure, or 24-hour diastolic blood pressure.

**References**

1. Li W, Schram MT, Sorensen BM, van Agtmaal MJM, Berendschot T, Webers CAB, Jansen JFA, Backes WH, Gronenschild E, Schalkwijk CG, Stehouwer CDA and Houben A. Microvascular Phenotyping in the Maastricht Study: Design and Main Findings, 2010-2018. *Am J Epidemiol*. 2020;189:873-884.

2. Vrooman HA, Cocosco CA, van der Lijn F, Stokking R, Ikram MA, Vernooij MW, Breteler MM and Niessen WJ. Multi-spectral brain tissue segmentation using automatically trained k-Nearest-Neighbor classification. *Neuroimage*. 2007;37:71-81.

3. de Boer R, Vrooman HA, van der Lijn F, Vernooij MW, Ikram MA, van der Lugt A, Breteler MM and Niessen WJ. White matter lesion extension to automatic brain tissue segmentation on MRI. *Neuroimage*. 2009;45:1151-61.

4. Wardlaw JM, Smith EE, Biessels GJ, Cordonnier C, Fazekas F, Frayne R, Lindley RI, O'Brien JT, Barkhof F, Benavente OR, Black SE, Brayne C, Breteler M, Chabriat H, Decarli C, de Leeuw FE, Doubal F, Duering M, Fox NC, Greenberg S, Hachinski V, Kilimann I, Mok V, Oostenbrugge R, Pantoni L, Speck O, Stephan BC, Teipel S, Viswanathan A, Werring D, Chen C, Smith C, van Buchem M, Norrving B, Gorelick PB, Dichgans M and nEuroimaging STfRVco. Neuroimaging standards for research into small vessel disease and its contribution to ageing and neurodegeneration. *Lancet Neurol*. 2013;12:822-38.

5. Gregoire SM, Chaudhary UJ, Brown MM, Yousry TA, Kallis C, Jager HR and Werring DJ. The Microbleed Anatomical Rating Scale (MARS): reliability of a tool to map brain microbleeds. *Neurology*. 2009;73:1759-66.

6. Bart M. ter Haar Romeny EJB, Jiong Zhang, Samaneh Abbasi-Sureshjani, Fan Huang, Remco Duits, Behdad Dashtbozorg, Tos T. J. M. Berendschot, Iris Smit-Ockeloen, Koen A. J. Eppenhof, Jinghan Feng, Julius Hannink, Jan Schouten, Mengmeng Tong, Hanhui Wu, Han W. van Triest, Shanshan Zhu, Dali Chen, Wei He, Ling Xu, Ping Han & Yan Kang Brain-inspired algorithms for retinal image analysis. *Machine Vision and Applications*. 2016;27: 1117–1135.

7. Erik Bekkers RD, Tos Berendschot, and Romeny BtH. A Multi-Orientation Analysis Approach to Retinal Vessel

Tracking. *J Math Imaging Vis*. 2013.

8. Jonas JB, Gusek GC and Naumann GO. Optic disc, cup and neuroretinal rim size, configuration and correlations in normal eyes. *Invest Ophthalmol Vis Sci*. 1988;29:1151-8.

9. Knudtson MD, Lee KE, Hubbard LD, Wong TY, Klein R and Klein BE. Revised formulas for summarizing retinal vessel diameters. *Curr Eye Res*. 2003;27:143-9.

10. Nagel E and Vilser W. Flicker observation light induces diameter response in retinal arterioles: a clinical methodological study. *Br J Ophthalmol*. 2004;88:54-6.

11. Nagel E, Vilser W, Fink A and Riemer T. [Variance of retinal vessel diameter response to flicker light. A methodical clinical study]. *Ophthalmologe*. 2006;103:114-9.

12. Sorensen BM, van der Heide FCT, Houben A, Koster A, T TJMB, J SAGS, Kroon AA, van der Kallen CJH, Henry RMA, van Dongen M, S JPME, H HCMS, van der Berg JD, Schaper NC, Schram MT and Stehouwer CDA. Higher levels of daily physical activity are associated with better skin microvascular function in type 2 diabetes-The Maastricht Study. *Microcirculation*. 2020;27:e12611.

13. van Dooren FE, Schram MT, Schalkwijk CG, Stehouwer CD, Henry RM, Dagnelie PC, Schaper NC, van der Kallen CJ, Koster A, Sep SJ, Denollet J, Verhey FR and Pouwer F. Associations of low grade inflammation and endothelial dysfunction with depression - The Maastricht Study. *Brain Behav Immun*. 2016;56:390-6.

14. van Bussel BC, Ferreira I, van de Waarenburg MP, van Greevenbroek MM, van der Kallen CJ, Henry RM, Feskens EJ, Stehouwer CD and Schalkwijk CG. Multiple inflammatory biomarker detection in a prospective cohort study: a cross-validation between well-established single-biomarker techniques and an electrochemiluminescense-based multi-array platform. *PLoS One*. 2013;8:e58576.

15. Stehouwer CDA. Microvascular Dysfunction and Hyperglycemia: A Vicious Cycle With Widespread Consequences. *Diabetes*. 2018;67:1729-1741.

16. Hutcheon JA, Chiolero A and Hanley JA. Random measurement error and regression dilution bias. *BMJ*. 2010;340:c2289.

**Supplemental Results**

Additional analyses

Quantitatively similar results were observed in a range of additional analyses. First, all cardiovascular risk factors statistically significantly modified the association of total alcohol consumption with the total MVD composite score (P_interaction_=0.02 for glucose metabolism status, P_interaction_<0.001 for hypertension, P_interaction_=0.046 for dyslipidemia, and P_interaction_=0.045 for current smoking; Supplemental Table S3 shows all P-for-interaction values). In stratified analyses, higher than light total alcohol consumption, wine, beer, and spirits consumption was generally more strongly associated with less MVD in individuals with, versus without, a cardiovascular risk factor (Supplemental Table S6 and Supplemental Figures S3 and S4). In addition, wine consumption was generally more strongly associated with less MVD than, consecutively, beer or spirits consumption (Supplemental Table S6 and Supplemental Figures S3 and S4). Second, we found that the strength of the association between total alcohol consumption and the total MVD composite score was increasingly stronger in individuals with increasingly more cardiovascular risk factors (Supplemental Table S7 and Supplemental Figure S5). Additionally, the minimum of the J-curve was at approximately 1 unit/day of total alcohol consumption in individuals without any cardiovascular risk factors and between 2 and 6 units/day in individuals with any risk factor, e.g. 4 units in individuals with type 2 diabetes (Supplemental Figure S6). Third, we found that a third order polynomial function, but not a fourth or fifth order polynomial function, statistically significantly better than a first order polynomial (i.e. linear) function described the association of total alcohol consumption with the total MVD composite score (P-value x^3^ term =0.02). When we plotted the association for a third order polynomial function we found that the shape of curve did not materially differ from the shape of the curve of a second order polynomial (i.e. quadratic) function (Supplemental Figure S7). Fourth, we found that that after full adjustment (model 3B) the minimum of the J curve for the association of total alcohol consumption with the total MVD composite score was located at 5.6 units of total alcohol consumption per day in the complete study population; 7.6 and 4.1 units of alcohol consumption per day in, respectively, individuals with and without a history of cardiovascular disease; and 6.1 and 3.1 units of total alcohol consumption per day in, respectively, men and women. Fifth, when we left wine or beer consumption out of the total alcohol consumption index the minimum of the J-curve was, respectively, higher and lower (Supplemental Figure S8). Further, the shape of the J-curve changed somewhat when wine or beer, but not spirits, consumption was left out of the total alcohol consumption index: without wine the sharp U-curve (at the minimum of the J curve) disappeared; without beer this U-curve became sharper; and without spirits the J-curve did not materially differ from the J-curve in the complete study population (Supplemental Figure S8). Sixth, we observed numerically similar associations when we analyzed the associations of total alcohol consumption with the total MVD composite score, when such a composite score was composed out of any five instead of six endpoints (Supplemental Table S8). Seventh, we overall had similar findings for associations of total alcohol, wine, beer, and spirits consumption with individual measures of MVD, both in the general population (Supplemental Tables S9-S11 and Supplemental Figure S9; only shown for total alcohol consumption) as well as in substrata of individuals with and without a history of cardiovascular disease (Supplemental Tables S11 and S12 and Supplemental figure S10; only shown for total alcohol consumption). In addition, we generally had similar findings in analyses with individual measures of MVD that were stratified by cardiovascular risk factor status (Supplemental Tables S13-S16 and Supplemental Figures S11-S14; only shown for total alcohol consumption). Of note, of all the associations of alcohol consumption with individual measures of MVD used in the total MVD composite score, associations of alcohol consumption with heat-induced skin hyperemia were relatively the least strong. Eighth, associations did not materially change after additional adjustment for accelerometer-assessed physical activity, diabetic retinopathy, eGFR, or plasma biomarkers of low-grade inflammation (Supplemental Table S17). Sixth, HbA1c (P_interaction_=0.01), fasting plasma glucose (P_interaction_=0.07), and 2-hour post load glucose (P_interaction_=0.08) (borderline) significantly modified associations of alcohol consumption with the total MVD composite score (Supplemental Table S3). Last, associations under study were numerically nearly identical when educational status was replaced with income level or occupational status; or when glucose metabolism status was replaced with fasting plasma glucose, 2-hour post load glucose, or HbA1c; or when office systolic blood pressure was replaced with office diastolic blood pressure, or systolic or diastolic 24-hour ambulatory blood pressure (Supplemental Table S18).

**Supplemental Figures**

**Total alcohol consumption and the total MVD composite score, in individuals with a**

**history of cardiovascular disease**

**
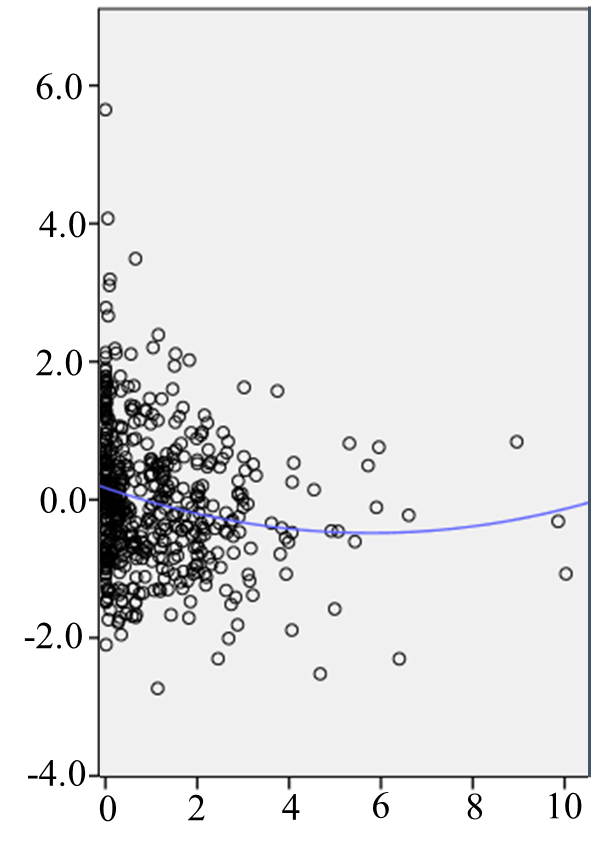
**

**Z-score Total MVD composite score**

**Total alcohol consumption, per unit**

**Supplemental Figure S1.1 With history of cardiovascular disease (N=595; minimum at 6 units/day)**

**Total alcohol consumption and the total MVD composite score, in individuals without a history of cardiovascular disease**


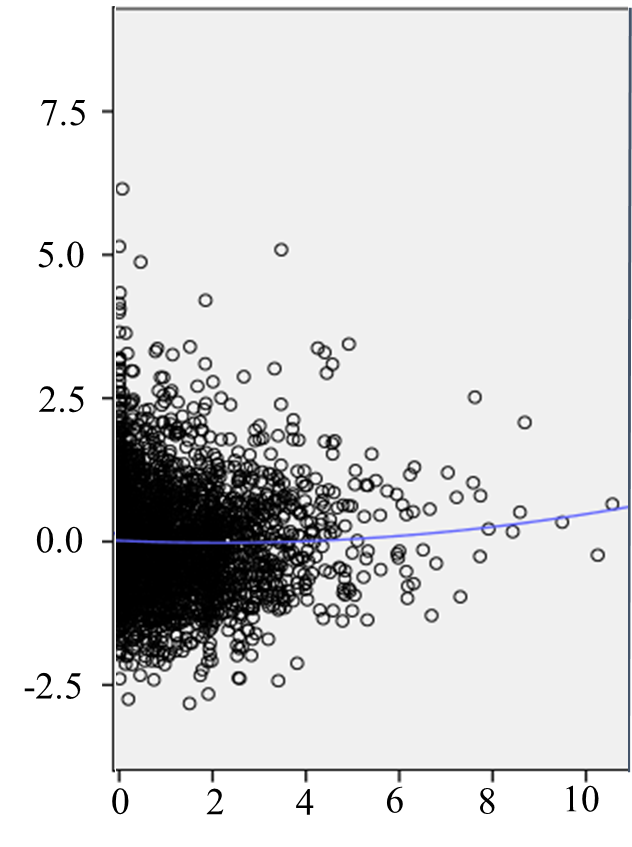


**Z-score Total MVD composite score**

**Total alcohol consumption, per unit**

**Supplemental Figure S1.2 Without history of cardiovascular disease (N=2,595; minimum at 2 units/day)**

**Total alcohol consumption and the total MVD composite score, in men**

**
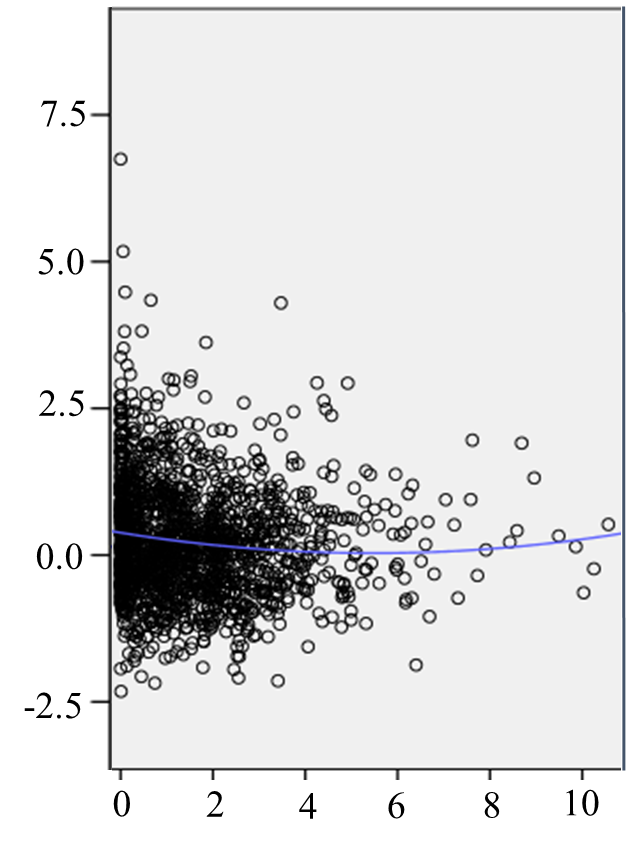
**

**Z-score Total MVD composite score**

**Total alcohol consumption, per unit**

**Supplemental Figure S1.3 Men (N=1,592; minimum at 5 units/day)**

**Total alcohol consumption and the total MVD composite score, in women**


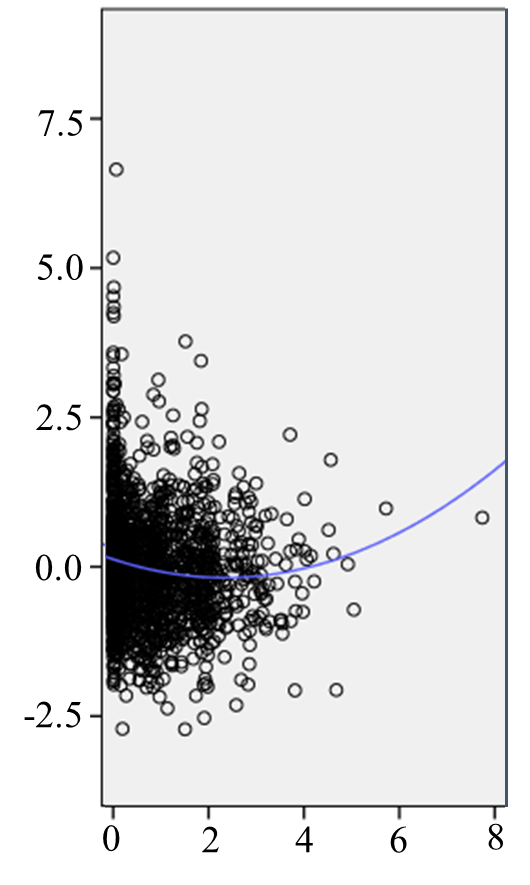


**Z-score Total MVD composite score**

**Total alcohol consumption, per unit**

**Supplemental Figure S1.4 Women (N=1,528; minimum at 3 units per day)**

Supplemental Figure S1 shows scatter plots with data points for total alcohol consumption (x-axis; per unit) and the total MVD composite score (y-axis; per SD) where a quadratic association was modeled (blue line). In individuals with a history of cardiovascular disease the minimum of the J-curve was at approximately 6 units/day (Supplemental Figure 1.1). In individuals without a history of cardiovascular disease the minimum of the J-curve was at approximately 2 units/day (Supplemental Figure 1.2). In men the minimum of the J-curve was at approximately 5 units/day (Supplemental Figure 1.3). In women the minimum of the J-curve was at approximately 2 units/day (Supplemental Figure 1.4).

Abbreviations: MVD, microvascular dysfunction; SD, standard deviation.

Supplemental Figure S2 shows associations of total alcohol, wine, beer, and spirits consumption with the total MVD composite score, in the general population and stratified by history of cardiovascular disease.

Betas and 95% confidence intervals indicate the strength of the associations of total alcohol consumption, wine, beer, or spirits consumption (per unit) with the total MVD composite score (per SD) where a negative beta indicates less MVD. The numbers included in the analyses and the numerical values per SD for all endpoints are reported in legends of Table 2 (general population) and Supplemental Table S4 (history of cardiovascular disease strata).

Variables included in model 3B are age, sex, glucose metabolism status, education level, waist circumference, smoking status, diet score, office systolic blood pressure, use of antihypertensive medication, total cholesterol / HDL cholesterol ratio, lipid-modifying medication, and history of cardiovascular disease (where applicable).

* indicates P-value<0.05.

Abbreviations: B, beta; CI: confidence interval; SD: standard deviation; MVD, microvascular dysfunction.

Supplemental Figure S3 shows associations of total alcohol consumption with the total MVD composite score, in the general population and stratified by cardiovascular risk factor status

Betas and 95% confidence intervals indicate the strength of the associations of total alcohol consumption (high versus light) with the total MVD composite score (per SD) where a negative beta indicates less MVD. The numbers included in the analyses and the numerical values per SD for all endpoints are reported in the legends of Table 2 (general population) and Supplemental Table S6 (cardiovascular risk factors).

Variables included in model 3B are age, sex, glucose metabolism status (where applicable), education level, waist circumference, smoking status (where applicable), diet score, office systolic blood pressure, use of antihypertensive medication (where applicable), total cholesterol / HDL cholesterol ratio, lipid-modifying medication (where applicable), and history of cardiovascular disease.

* indicates P-value<0.05.

Abbreviations: B, beta; CI: confidence interval; SD: standard deviation; MVD, microvascular dysfunction.

Supplemental Figure S4 shows associations of wine, beer, and spirits consumption with the total MVD composite score, stratified by cardiovascular risk factor status.

Betas and 95% confidence intervals indicate the strength of the associations wine, beer, or spirits consumption (per unit) with the total MVD composite score (per SD) where a negative beta indicates less MVD. The numbers included in the analyses and the numerical values per SD for all endpoints are reported in the legends of Table 2 (general population) and Supplemental Table S6 (cardiovascular risk factors).

Variables included in model 3B are age, sex, glucose metabolism status (where applicable), education level, waist circumference, smoking status (where applicable), diet score, office systolic blood pressure, use of antihypertensive medication (where applicable), total cholesterol / HDL cholesterol ratio, lipid-modifying medication (where applicable), and history of cardiovascular disease.

* indicates P-value<0.05.

Abbreviations: B, beta; CI: confidence interval; SD: standard deviation; MVD, microvascular dysfunction.

Supplemental Figure S5 shows associations of total alcohol consumption with the total MVD composite score, stratified by number of cardiovascular risks or history of cardiovascular disease (model 3B)

Betas and 95% confidence intervals indicate the strength of the associations of total alcohol consumption (high versus light) with the total MVD composite score (per SD) where a negative beta indicates less MVD. The numbers included in the analyses and the numerical values per SD for all endpoints are reported in legends of Supplemental Table S4 (history of cardiovascular disease) and Supplemental Table S6 (cardiovascular risk factors)..

Variables included in model 3B are age, sex, glucose metabolism status (where applicable), education level, waist circumference, smoking status (where applicable), diet score, office systolic blood pressure, use of antihypertensive medication (where applicable), total cholesterol / HDL cholesterol ratio, lipid-modifying medication (where applicable), and history of cardiovascular disease (where applicable).

* indicates P-value<0.05.

Abbreviations: B, beta; CI: confidence interval; SD: standard deviation; MVD, microvascular dysfunction.

**Total alcohol consumption and the total MVD composite score, in individuals with normal glucose metabolism and without hypertension, without dyslipidemia, and without current smoking**

**
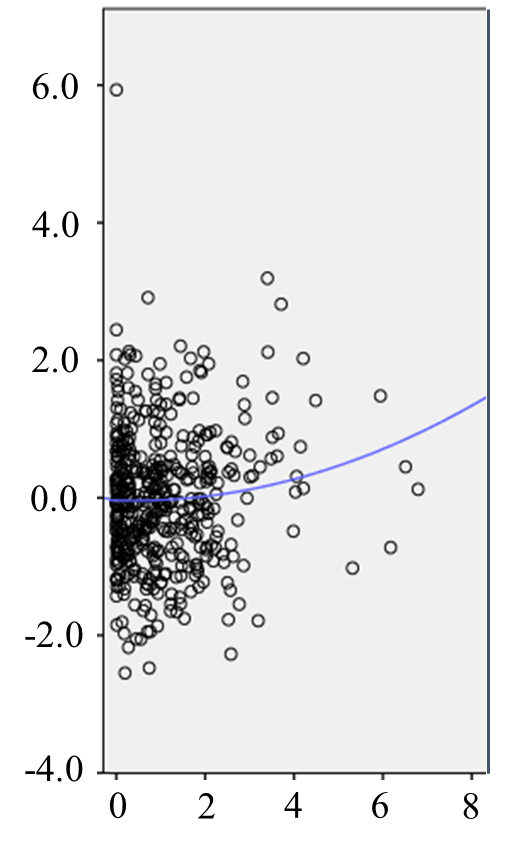
**

**Z-score Total MVD composite score**

**Total alcohol consumption, per unit**

**Supplemental Figure S6.1**

**Total alcohol consumption and the total MVD composite score, stratified by glucose metabolism status**

**
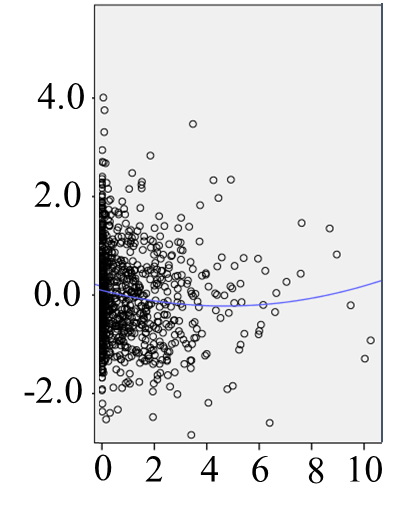
**


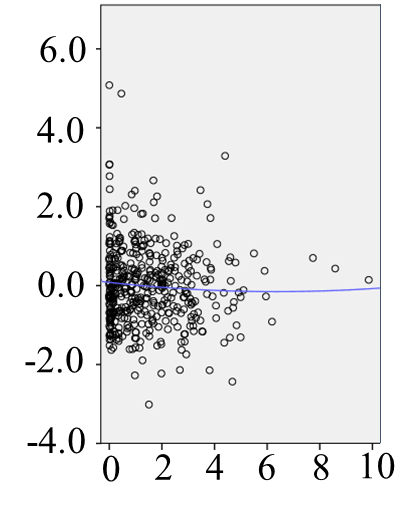

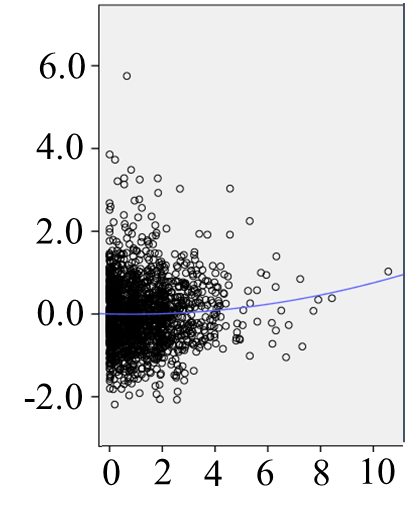


**Z-score Total MVD composite score**

**Z-score Total MVD composite score**

**Z-score Total MVD composite score**

**Z-score Total MVD composite score**

**Total alcohol consumption, per unit**

**Total alcohol consumption, per unit**

**Total alcohol consumption, per unit**

**Type 2 diabetes**

**Normal glucose metabolism**

**Prediabetes**

**Supplemental Figure S6.2**

**Total alcohol consumption and the total MVD composite score, stratified by hypertension status**

**
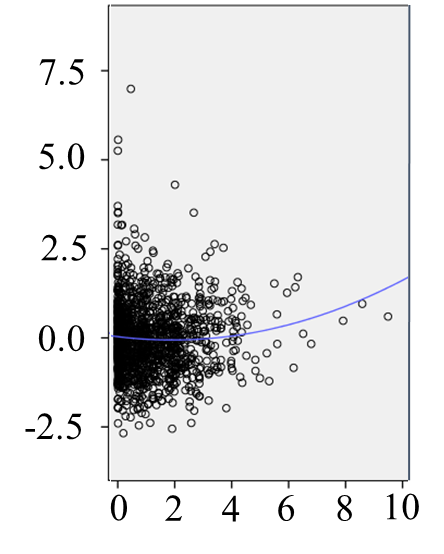
**

**
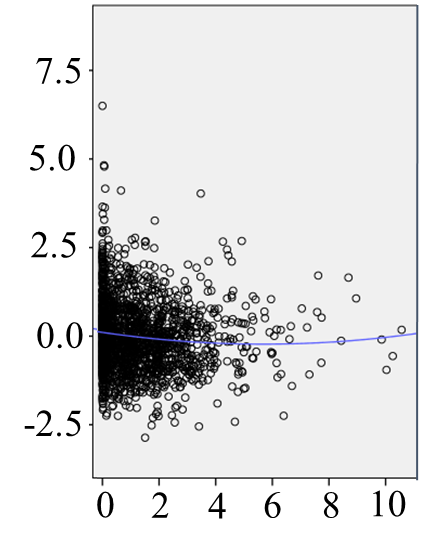
**

**Z-score Total MVD composite score**

**Z-score Total MVD composite score**

**Total alcohol consumption, per unit**

**Total alcohol consumption, per unit**

**With hypertension**

**Without hypertension**

**Supplemental Figure S6.3**

**Total alcohol consumption and the total MVD composite score, stratified by dyslipidemia status**

**
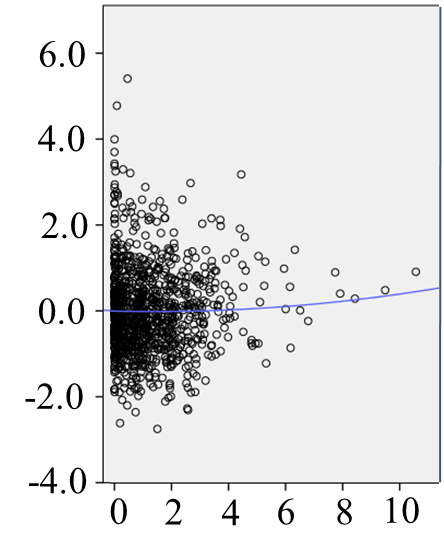
**

**
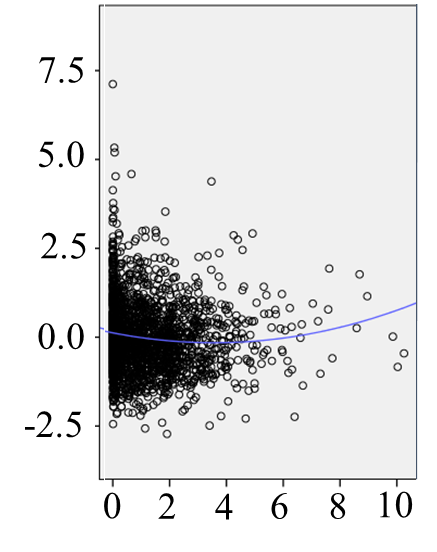
**

**Z-score Total MVD composite score**

**Z-score Total MVD composite score**

**Total alcohol consumption, per unit**

**Total alcohol consumption, per unit**

**With dyslipidemia**

**Without dyslipidemia**

**Supplemental Figure S6.4**

**Total alcohol consumption and the total MVD composite score, stratified by current smoking status**

**
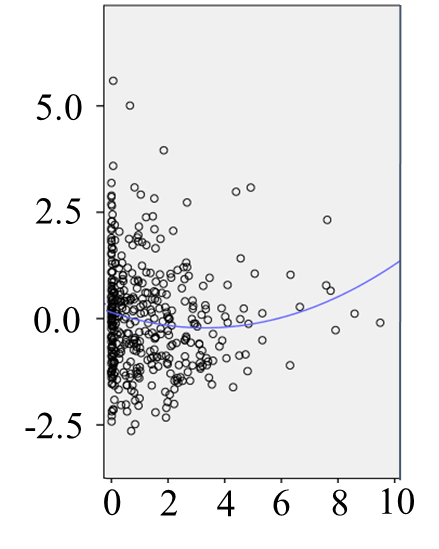

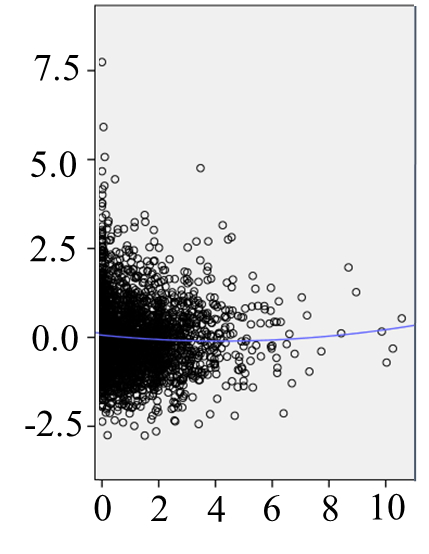
**

**Z-score Total MVD composite score**

**Z-score Total MVD composite score**

**Total alcohol consumption, per unit**

**Total alcohol consumption, per unit**

**With current smoking**

**Without current smoking**

**Supplemental Figure S6.5**

Supplemental Figure S6 shows scatter plots with data points for total alcohol consumption (x-axis; per unit) and the total MVD composite score (y-axis; per SD) where a quadratic association was modeled (blue line). Supplemental Figure S6 shows the scatter plot in individuals without any risk factors (n=450; S6.1), or stratified by glucose metabolism status (S6.2), hypertension status (S6.3), dyslipidemia status (S6.4), or current smoking status (S6.5). The number of participants per stratum is reported in the legend of Supplemental Table S6.

Abbreviations: MVD, microvascular dysfunction; SD, standard deviation.

**Total alcohol consumption and the total MVD composite score, where a third degree polynomial function was modeled in the complete study population**

**
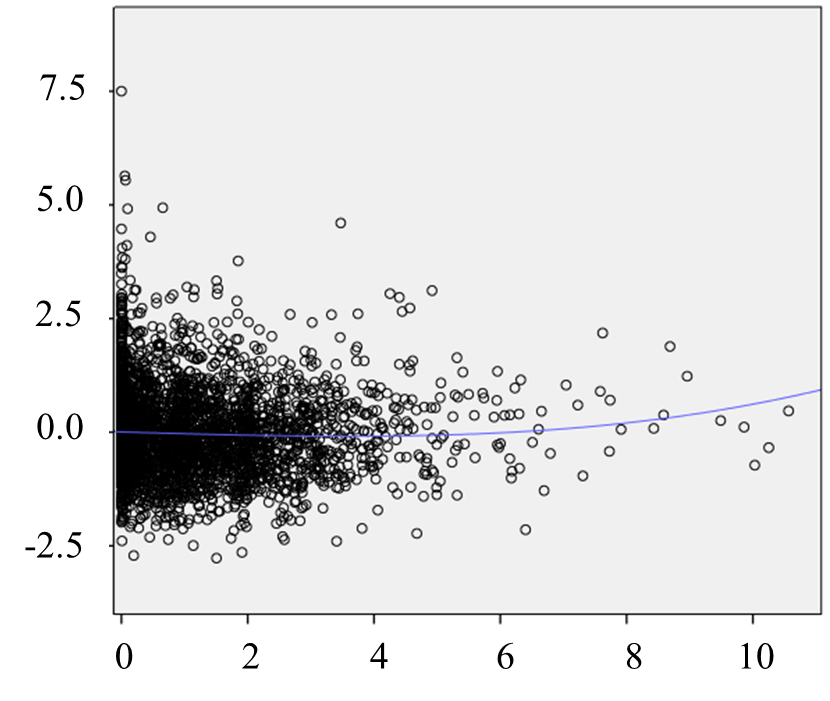
**

**Z-score Total MVD composite score**

**Total alcohol consumption, per unit**

Supplemental Figure S7 shows a scatter plot with data points for total alcohol consumption (x-axis; per unit) and the total MVD composite score (y-axis; per SD) where a third degree polynomial function was modeled (y=x +x^3^; blue line) in the complete study population (n=3,120).

Abbreviations: MVD, microvascular dysfunction; SD, standard deviation.


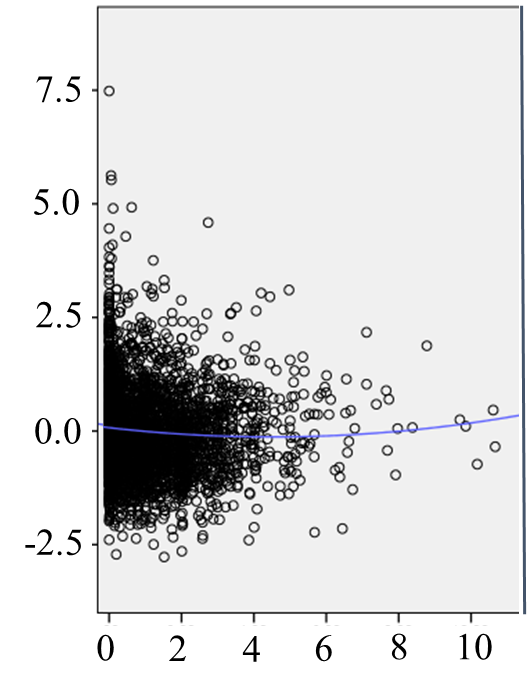

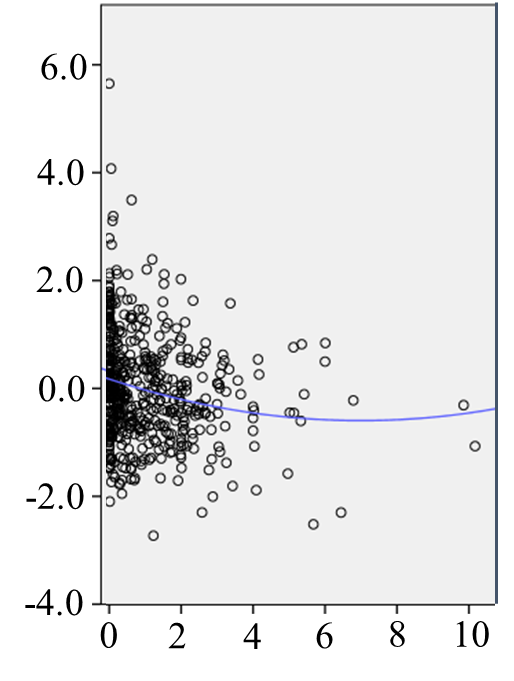


**Z-score Total MVD composite score**

**Z-score Total MVD composite score**

**Total alcohol consumption, per unit**

**Total alcohol consumption, per unit**

**General population**

**With a history of cardiovascular disease**

**Supplemental Figure S8.1 Total alcohol consumption without spirits (i.e. only wine and beer)**

**
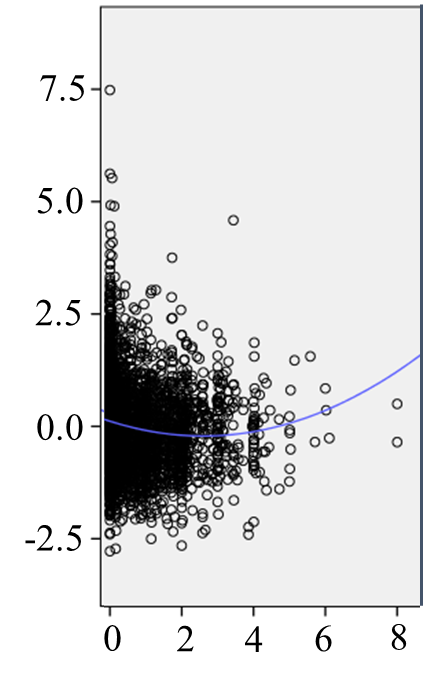

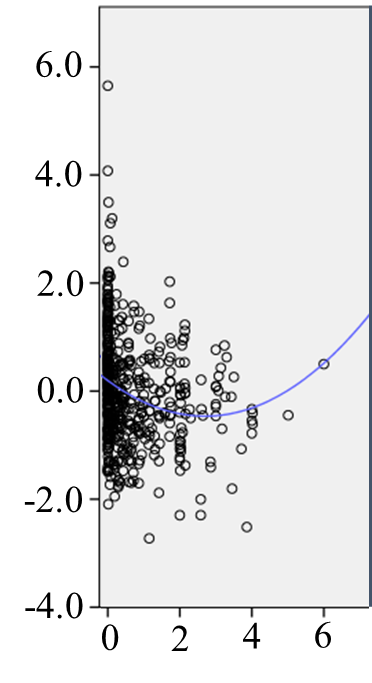
**

**Z-score Total MVD composite score**

**Z-score Total MVD composite score**

**Total alcohol consumption, per unit**

**Total alcohol consumption, per unit**

**General population**

**With a history of cardiovascular disease**

**Supplemental Figure S8.2 Total alcohol consumption without beer (i.e. only wine and spirits)**

**
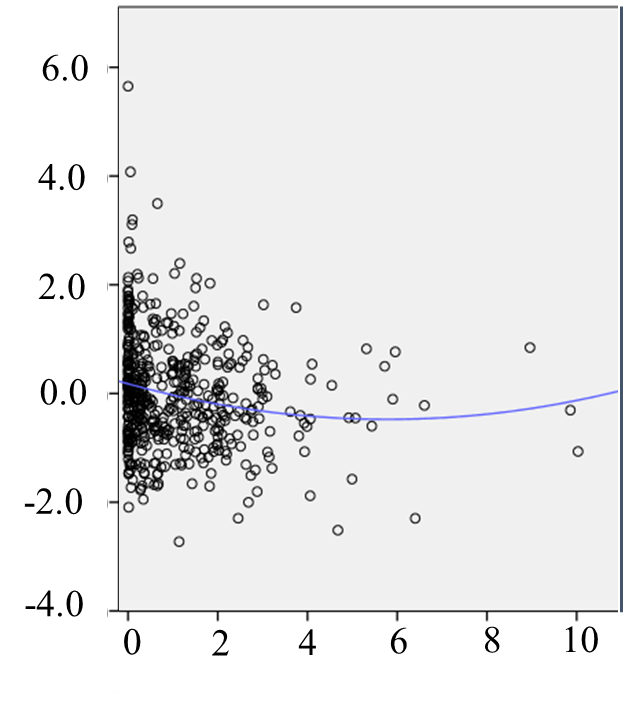

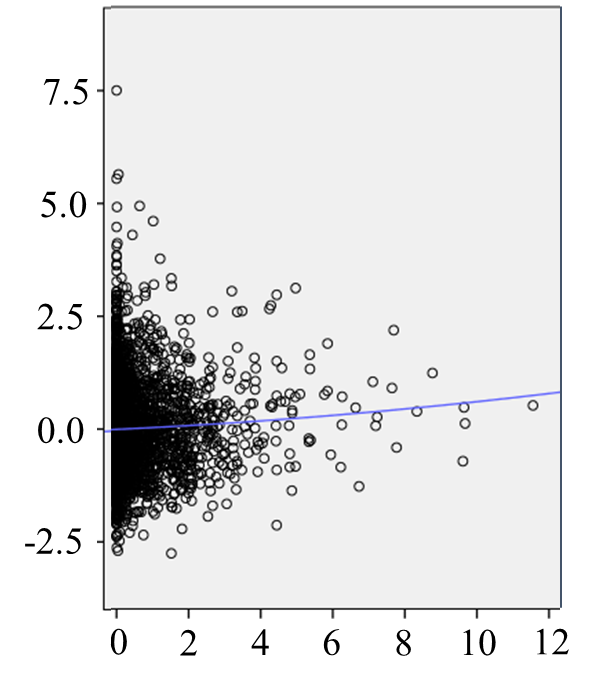
**

**Z-score Total MVD composite score**

**Z-score Total MVD composite score**

**Total alcohol consumption, per unit**

**Total alcohol consumption, per unit**

**General population**

**With a history of cardiovascular disease**

**Supplemental Figure S8.3 Total alcohol consumption without wine (i.e. only beer and spirits)**

Supplemental Figure S8 shows scatter plots with data points for total alcohol consumption (x-axis; per unit) and the total MVD composite score (y-axis; per SD) where a quadratic association was modeled (blue line). Supplemental Figure S8 shows the scatter plots in the general population and in individuals with a history of cardiovascular disease for total alcohol consumption without spirits (S8.1), without beer (S8.2), and without wine (S8.3). The number of participants per stratum are reported in the legend of Table 2 (general population) and Supplemental Table S4 (history of cardiovascular disease strata).

Abbreviations: MVD, microvascular dysfunction; SD, standard deviation.

**Associations of total alcohol consumption (per unit) with measures of MVD (in SD; model 3B), in the general population**

Supplemental Figure S9 shows associations of total alcohol consumption with CSVD features, retinal microvascular diameters and MVD measures, where MVD was estimated from flicker light-induced increase in retinal microvascular diameters, heat-induced skin hyperemia, UAE, and plasma biomarkers of MVD. Betas and 95% confidence intervals indicate the strength of the associations of total alcohol consumption (per unit) with measures of MVD (per SD) where a negative beta indicates less MVD. The number of participants in analyses and the numerical values per SD for all endpoints are reported in the legend of Table 2.

Variables included in model 3B are age, sex, glucose metabolism status, education level, waist circumference, smoking status, diet score, office systolic blood pressure, use of antihypertensive medication, total cholesterol / HDL cholesterol ratio, lipid-modifying medication, and history of cardiovascular disease. Additionally, for associations with heat-induced skin hyperemia baseline skin blood flow was entered in the model.

For UAE, the beta per unit of total alcohol consumption corresponds with an odds ratio of 0.98 (95% CI, 0.96; 1.01) for 30mg/24-hour greater urinary albumin excretion.

* indicates P-value<0.05.

Abbreviations: B, beta; CI: confidence interval; SD: standard deviation; MVD, microvascular dysfunction.

**Associations of total alcohol consumption (high versus light) with measures of MVD (in SD; model 3B), stratified by history of cardiovascular disease status
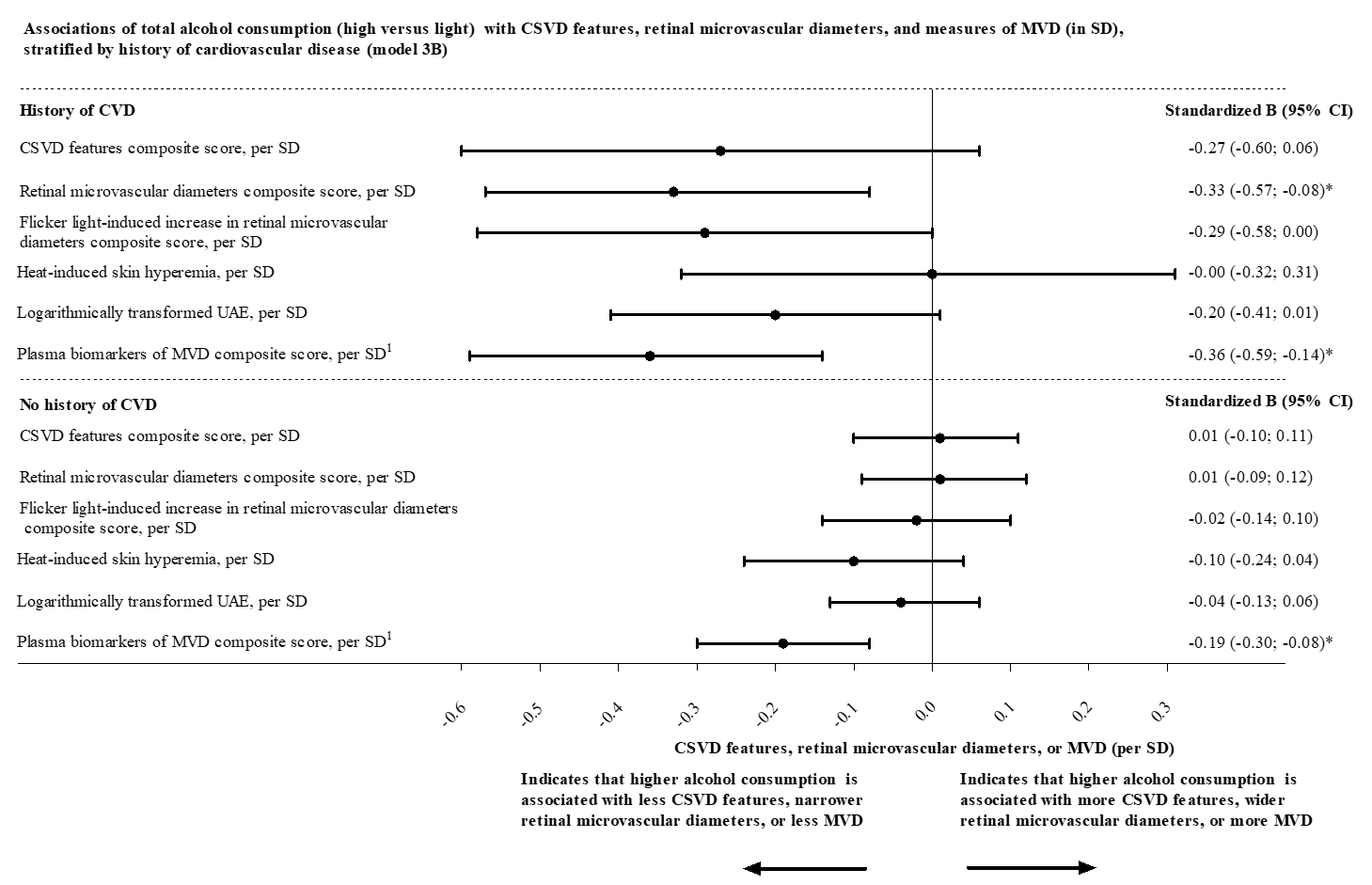
**

Supplemental Figure S10 shows history of cardiovascular disease-stratified associations of total alcohol consumption with CSVD features, retinal microvascular diameters and MVD measures, where MVD was estimated from flicker light-induced increase in retinal microvascular diameters, heat-induced skin hyperemia, UAE, and plasma biomarkers of MVD. Betas and 95% confidence intervals indicate the strength of the associations of total alcohol consumption (per unit) with measures of MVD (per SD) where a negative beta indicates less MVD. The number of participants in analyses and the numerical values per SD for all endpoints are reported in the legend of Supplemental Table S4.

Variables included in model 3B are age, sex, glucose metabolism status, education level, waist circumference, smoking status, diet score, office systolic blood pressure, use of antihypertensive medication, total cholesterol / HDL cholesterol ratio, and lipid-modifying medication. Additionally, for associations with heat-induced skin hyperemia baseline skin blood flow was entered in the model.

^1^ For the associations with plasma biomarkers of MVD composite score, the association for light versus none total alcohol consumption is presented instead of the association for high versus light total alcohol consumption.

For UAE, the beta of high versus light total alcohol consumption corresponds with an odds ratio of 0.79 (95% CI, 0.62; 1.01) and 0.97 (95% CI, 0.89; 1.05) for 30mg/24-hour greater urinary albumin excretion for individuals with and without a history of cardiovascular disease .

* indicates P-value<0.05.

Abbreviations: B, beta; CI, confidence interval; SD: standard deviation; UAE, urinary albumin excretion; MVD, microvascular dysfunction**.**

**Associations of total alcohol consumption (high versus light) with measures of MVD (in SD; model 3B), stratified by glucose metabolism status**

Supplemental Figure S11 shows glucose metabolism status-stratified associations of total alcohol consumption with CSVD features, retinal microvascular diameters and MVD measures, where MVD was estimated from flicker light-induced increase in retinal microvascular diameters, heat-induced skin hyperemia, UAE, and plasma biomarkers of MVD. Betas and 95% confidence intervals indicate the strength of the associations of total alcohol consumption (per unit) with measures of MVD (per SD) where a negative beta indicates less MVD. The number of participants in analyses and the numerical values per SD for all endpoints are reported in the legend of Supplemental Table S6.

Variables entered in model 3B are age, sex, education level, waist circumference, smoking status, diet score, office systolic blood pressure, use of antihypertensive medication, total cholesterol / HDL cholesterol ratio, lipid-modifying medication, and prior cardiovascular disease. Additionally, for associations with heat-induced skin hyperemia baseline skin blood flow was entered in the model.

^1^ indicates that the association for moderate versus light total alcohol consumption is presented instead of the association for high versus light total alcohol consumption.

^2^ indicates that the association for light versus none total alcohol consumption is presented instead of the association for high versus light total alcohol consumption.

For UAE, the beta for total alcohol consumption (high versus light) corresponds with an odds ratio of 0.79 (95% CI, 0.65; 0.96) for 30mg/24-hour greater urinary albumin excretion in individuals with type 2 diabetes, 0.88 (95% CI, 0.69; 1.12) for 30mg/24-hour greater urinary albumin excretion in individuals with prediabetes (for moderate versus light instead of high versus light), and 1.01 (95% CI, 0.92; 1.12) for 30mg/24-hour greater urinary albumin excretion in individuals with normal glucose metabolism (for high versus light).

* indicates P-value<0.05.

Abbreviations: B, beta; CI, confidence interval; SD: standard deviation; UAE, urinary albumin excretion; MVD, microvascular dysfunction.

**Associations of total alcohol consumption (high versus light) with measures of MVD (in SD; model 3B), stratified by hypertension status**

Supplemental Figure S12 shows hypertension status-stratified associations of total alcohol consumption with CSVD features, retinal microvascular diameters and MVD measures, where MVD was estimated from flicker light-induced increase in retinal microvascular diameters, heat-induced skin hyperemia, UAE, and plasma biomarkers of MVD. Betas and 95% confidence intervals indicate the strength of the associations of total alcohol consumption (per unit) with measures of MVD (per SD) where a negative beta indicates less MVD. The number of participants in analyses and the numerical values per SD for all endpoints are reported in the legend of Supplemental Table S6.

Variables entered in model 3B are age, sex, glucose metabolism status, education level, waist circumference, smoking status, diet score, office systolic blood pressure, use of antihypertensive medication (where applicable), total cholesterol / HDL cholesterol ratio, lipid-modifying medication, and history of cardiovascular disease. Additionally, for associations with heat-induced skin hyperemia baseline skin blood flow was entered in the model.

^1^ indicates that the association for moderate versus light alcohol consumption is presented instead of the association for high versus light alcohol consumption.

^2^ indicates that the association for light versus none alcohol consumption is presented instead of the association for high versus light alcohol consumption.

For UAE, the beta for high versus light total alcohol consumption corresponds with an odds ratio of 0.92 (95% CI, 0.81; 1.04) and the beta for moderate versus light total alcohol consumption corresponds with an odds ratio of 0.92 (95% CI, 0.81; 1.02) for 30mg/24-hour greater urinary albumin excretion for individuals with and without hypertension.

Bold denotes P-value<0.05.

Abbreviations: B, beta; CI, confidence interval; SD: standard deviation; CSVD, cerebral small vessel disease; UAE, urinary albumin excretion; MVD, microvascular dysfunction; HDL, high-density lipoprotein.

**Associations of total alcohol consumption (high versus light) with measures of MVD (in SD; model 3B), stratified by dyslipidemia status**

Supplemental Figure S13 shows dyslipidemia status-stratified associations of total alcohol consumption with CSVD features, retinal microvascular diameters and MVD measures, where MVD was estimated from flicker light-induced increase in retinal microvascular diameters, heat-induced skin hyperemia, UAE, and plasma biomarkers of MVD. Betas and 95% confidence intervals indicate the strength of the associations of total alcohol consumption (per unit) with measures of MVD (per SD) where a negative beta indicates less MVD. The number of participants in analyses and the numerical values per SD for all endpoints are reported in the legend of Supplemental Table S6.

Variables entered in model 3B are age, sex, glucose metabolism status, education level, waist circumference, smoking status (former versus never smoker; only applicable in the analyses in individuals without [current] smoking), diet score, office systolic blood pressure, use of antihypertensive medication, total cholesterol / HDL cholesterol ratio, lipid-modifying medication (where applicable), and history of cardiovascular disease.

^1^ indicates that the association for moderate versus light alcohol consumption is presented instead of the association for high versus light alcohol consumption.

^2^ indicates that the association for light versus none alcohol consumption is presented instead of the association for high versus light alcohol consumption.

For UAE, the beta of total alcohol consumption (for high versus light) corresponds with an odds ratio of 0.93 (95% CI, 0.84; 1.03) and the beta of total alcohol consumption (for moderate versus light) 0.89 (95% CI, 0.77; 1.04) for 30mg/24-hour greater urinary albumin excretion for individuals with and without dyslipidemia.

Bold denotes P-value<0.05.

Abbreviations: B, beta; CI, confidence interval; SD: standard deviation; MVD, microvascular dysfunction; HDL, high-density lipoprotein.

**Associations of total alcohol consumption (high versus light) with measures of MVD (in SD; model 3B), stratified by current smoking status**

Supplemental Figure S14 shows current smoking status-stratified associations of total alcohol consumption with CSVD features, retinal microvascular diameters and MVD measures, where MVD was estimated from flicker light-induced increase in retinal microvascular diameters, heat-induced skin hyperemia, UAE, and plasma biomarkers of MVD. Betas and 95% confidence intervals indicate the strength of the associations of total alcohol consumption (per unit) with measures of MVD (per SD) where a negative beta indicates less MVD. The number of participants in analyses and the numerical values per SD for all endpoints are reported in the legend of Supplemental Table S6.

Variables entered in model 3B are age, sex, glucose metabolism status, education level, waist circumference, smoking status (former versus never smoker; only applicable in the analyses in individuals without current smoking), diet score, office systolic blood pressure, use of antihypertensive medication, total cholesterol / HDL cholesterol ratio, lipid-modifying medication, and history of cardiovascular disease.

^1^ indicates that the association for light versus none alcohol consumption is presented instead of the association for high versus light alcohol consumption.

^2^ indicates that the association for moderate versus light alcohol consumption is presented instead of the association for high versus light alcohol consumption.

Bold denotes P-value<0.05.

For UAE, the beta of total alcohol consumption (for moderate versus light) in model 3B correspond with the following odds ratios (95% CI) for 30mg/24-hour greater UAE of 0.74 (0.55; 1.01) in individuals with current smoking and 0.95 (0.86; 1.05) in individuals without current smoking.

Abbreviations: B, beta; CI, confidence interval; SD: standard deviation; CSVD, cerebral small vessel disease; UAE, urinary albumin excretion; MVD, microvascular dysfunction; HDL, high-density lipoprotein.

**Supplemental Tables**

**Supplemental Table S1 Characteristics of the study population and individuals excluded from the analyses due to missing values for the CSVD features, retinal microvascular diameters, and flicker light-induced increase in retinal microvascular diameters study populations**

|  | CSVD features | | | Retinal microvascular diameters | | | Flicker light-induced increase in retinal microvascular diameters | | |
| --- | --- | --- | --- | --- | --- | --- | --- | --- | --- |
|  | Complete (n=2,075) | Number of participants with missing data in complete / missing | Missing (n=1,376) | Complete (n=2,721) | Number of participants with missing data in complete / missing | Missing (n=730) | Complete (n=2,090) | Number of participants with missing data in complete / missing | Missing (n=1,361) |
| **Demographics** |  |  |  |  |  |  |  |  |  |
| Age, years | 59.4 ± 8.1 | 0/0 | 60.4 ± 8.4 | 59.8 ± 8.2 | 0/0 | 59.5 ± 8.7 | 59.6 ± 8.2 | 0/0 | 60.0 ± 8.4 |
| Men | 1,051 (50.7) | 0/0 | 724 (52.6) | 1,383 (50.8) | 0/0 | 392 (53.7) | 1,043 (49.9) | 0/0 | 732 (53.8) |
| **Lifestyle factors** |  |  |  |  |  |  |  |  |  |
| Smoking status:  Never  Former  Current | 780 (37.6)  1,059 (51.0)  236 (11.4) | 0/50 | 392 (29.6)  700 (52.8)  234 (17.6) | 957 (35.2)  1,431 (52.6)  333 (12.2) | 0/50 | 215 (31.6)  328 (48.2)  137 (20.1) | 744 (35.6)  1,097 (52.5)  249 (11.9) | 0/50 | 428 (32.6)  662 (50.5)  221 (16.9) |
| Body mass index, kg/m^2^ | 26.5 ± 4.1 | 0/3 | 27.9 ± 5.0 | 27.0 ± 4.5 | 1/2 | 27.4 ± 4.8 | 26.9 ± 4.5 | 1/2 | 27.4 ± 4.7 |
| Waist circumference, cm | 94.2 ± 12.6 | 0/4 | 98.6 ± 15.0 | 95.7 ± 13.7 | 0/4 | 96.8 ± 14.1 | 95.3 ± 13.7 | 0/4 | 97.0 ± 14.0 |
| Physical activity, hours/day | 2.1 ± 0.7 | 461/345 | 1.9 ± 0.7 | 2.0 ± 0.7 | 508/298 | 2.0 ± 0.7 | 2.0 ± 0.7 | 300/506 | 1.9 ± 0.7 |
| Dutch Healthy diet score, points | 84.0 ± 14.6 | 0/226 | 81.9 ± 14.8 | 83.6 ± 14.7 | 0/226 | 81.4 ± 14.9 | 83.8 ± 14.7 | 0/226 | 82.2 ±14.8 |
| **Cardiovascular risk factors** |  |  |  |  |  |  |  |  |  |
| Glucose metabolism status  Normal glucose metabolism  Prediabetes  Type 2 diabetes  Other types of diabetes | 1,274 (61.4)  321 (15.5)  459 (22.1) 21 (1.0) | 0/0 | 650 (47.2)  190 (13.8)  516 (37.5) 20 (1.5) | 1,533 (56.3)  403 (14.8)  758 (27.9) 27 (1.0) | 0/0 | 391 (53.6)  108 (14.8)  217 (29.7) 14 (1.9) | 1,197 (57.3)  307 (14.7)  563 (26.9) 23 (1.1) | 0/0 | 727 (53.4)  204 (15.0)  412 (30.3) 18 (1.3) |
| Fasting plasma glucose, mmol/l | 5.5 [5.0 – 6.2] | 0/1 | 5.7 [5.1 – 7.1] | 5.5 [5.0 – 6.5] | 1/0 | 5.6 [5.1 – 6.7] | 5.5 [5.0 – 6.5] | 0/1 | 5.6 [5.1 – 6.7] |
| 2-hour post load plasma glucose, mmol/l | 6.0 [4.9 – 8.5] | 114/178 | 6.8 [5.3 – 11.4] | 6.3 [5.1 – 9.4] | 206/86 | 6.3 [5.0 – 9.2] | 6.3 [5.1 – 9.1] | 148/144 | 6.3 [5.0 – 9.5] |
| HbA1c, % | 5.6 [5.3 – 6.0] | 4/9 | 5.8 [5.4 – 6.5] | 5.6 [5.3 – 6.2] | 6/7 | 5.8 [5.5 – 6.3] | 5.6 [5.3 – 6.2] | 3/10 | 5.8 [5.5 – 6.3] |
| Use of glucose-lowering medication | 361 (17.4) | 0/0 | 446 (32.4) | 624 (22.9) | 0/0 | 183 (25.1) | 463 (22.2) | 0/0 | 344 (25.3) |
| Total/HDL cholesterol ratio | 3.6 ± 1.2 | 0/4 | 3.6 ± 4.6 | 3.6 ± 1.1 | 0/4 | 3.9 ± 1.3 | 3.6 ± 1.1 | 0/4 | 3.8 ± 1.2 |
| Use of lipid-modifying medication | 647 (31.2) | 0/0 | 612 (44.5) | 999 (36.7) | 0/0 | 260 (35.6) | 745 (35.6) | 0/0 | 514 (37.8) |
| Office systolic blood pressure, mm Hg | 133.9 ± 17.3 | 0/2 | 136.8 ± 19.3 | 134.9 ± 18.1 | 0/2 | 135.6 ± 18.5 | 134.9 ± 18.1 | 0/2 | 135.4 ± 18.4 |
| Office diastolic blood pressure, mm Hg | 75.9 ± 9.8 | 0/2 | 76.6 ± 10.0 | 76.1 ± 9.9 | 0/2 | 76.2 ± 9.7 | 76.2 ± 9.9 | 0/2 | 76.1 ± 9.8 |
| Ambulatory systolic blood pressure, mm Hg | 118.4 ± 11.2 | 224/180 | 120.1 ± 12.7 | 118.9 ± 11.5 | 299/105 | 119.9 ± 12.8 | 118.9 ± 11.5 | 230/174 | 119.3 ± 12.3 |
| Ambulatory diastolic blood pressure, mm Hg | 73.4 ± 7.0 | 224/180 | 73.6 ± 7.4 | 73.4 ± 7.1 | 299/105 | 74.1 ± 7.2 | 73.3 ± 7.1 | 230/174 | 73.7 ± 7.2 |
| Use of antihypertensive medication | 728 (35.1) | 0/0 | 664 (48.3) | 1,105 (40.6) | 0/0 | 287 (39.3) | 804 (38.5) | 0/0 | 588 (43.2) |
| History of cardiovascular disease | 253 (12.2) | 0/67 | 315 (24.1) | 459 (16.9) | 0/67 | 109 (16.4) | 330 (15.8) | 0/67 | 238 (18.4) |
| Diabetic retinopathy | 22 (1.3) | 322/262 | 24 (2.2) | 41 (1.6) | 169/425 | 5 (1.6) | 33 (1.6) | 60/534 | 13 (1.6) |
| eGFR, ml/min/1.73^2^ | 88.7 ± 14.3 | 14/19 | 87.3 ± 15.8 | 88.2 ± 14.8 | 20/13 | 88.0 ± 15.5 | 88.2 ± 14.6 | 12/21 | 88.0 ± 15.4 |
| Plasma biomarkers of low-grade inflammation  C-reactive protein, µg/ml  Serum amyloid A, µg/ml  Tumor necrosis factor alpha, pg/ml  Interleukin-6, pg/ml  Interleukin-8, pg/ml | 1.2 [0.6 – 2.5]  3.2 [2.0 – 5.4]  2.2 [1.9 – 2.5]  0.6 [0.4 – 0.8]  4.0 [3.2 – 5.1] | 14/25 | 1.4 [0.7 – 3.2]  3.3 [2.1 – 5.6]  2.3 [1.9 – 2.7]  0.7 [0.4 – 1.0]  4.4 [3.4 – 5.7] | 1.2 [0.6 – 2.7]  3.3 [2.1 – 5.5]  2.2 [1.9 – 2.6]  0.6 [0.4 – 0.9]  4.1 [3.3 – 5.3] | 25/14 | 1.4 [0.6 – 3.1]  3.1 [2.0 – 5.5]  2.2 [1.9 – 2.5]  0.6 [0.4 – 0.9]  5.5 [4.3 – 7.4] | 1.2 [0.6 – 2.7]  3.3 [2.1 – 5.4]  2.2 [1.9 – 2.6]  0.6 [0.4 – 0.9]  4.1 [3.2 – 5.3] | 16/23 | 1.3 [0.6 – 3.0]  3.2 [2.0 – 5.5]  2.2 [1.9 – 2.5]  0.6 [0.4 – 0.9]  4.3 [3.4 – 5.5] |
| **Other** |  |  |  |  |  |  |  |  |  |
| Educational status  Low   Middle  High | 630 (30.4)  606 (29.2)  839 (40.4) | 0/74 | 505 (38.8)  348 (26.7)  449 (34.5) | 910 (33.4)  777 (28.6)  1034 (38.0) | 0/74 | 225 (34.3)  177 (27.0)  254 (38.7) | 676 (32.3) 601 (28.8) 813 (38.9) | 0/74 | 459 (35.7) 353 (27.4) 475 (36.9) |
| Occupational status  Low   Middle  High | 508 (22.8)  651 (36.9)  603 (34.2) | 313/314 | 385 (36.3)  344 (32.4)  333 (31.4) | 708 (31.3)  808 (35.7)  749 (33.1) | 456/171 | 185 (33.1)  187 (33.5)  187 (33.5) | 539 (30.9) 642 (36.8) 564 (32.3) | 345/282 | 354 (32.8) 353 (32.7) 372 (34.5) |
| Income per month, euros | 2,070 ± 821 | 452/416 | 1,922 ± 808 | 2012 ± 806 | 641/227 | 2,024 ± 873 | 2034 ± 804 | 471/397 | 1,982 ± 843 |
| **Alcohol consumption** |  |  |  |  |  |  |  |  |  |
| Total alcohol consumption, units/day  None  Light  Moderate  High  Total wine consumption, units/day  None  Light  Moderate  High  Total beer consumption, units/day  None  Light  Moderate  High  Total spirits consumption, units /day  None  Light  Moderate  High | 0.9 [0.2 – 1.9]  313 (15.1)  627 (30.2)  422 (20.3) 713 (34.4)  0.3 [0.0 – 1.2]  772 (37.2)  527 (25.4)  347 (16.7) 429 (20.7)  0.1 [0.0 – 0.5] 1,184 (57.1)  543 (26.2)  206 (10.0) 140 (6.7)  0.0 [0.0 – 0.0] 1,870 (90.1)  173 (8.3)  25 (1.2) 7 (0.3) | 0/226 | 0.7 [0.1 – 1.7]  207 (18.0)  387 (33.7)  220 (19.1) 336 (24.4)  0.2 [0.0 – 1.0]  559 (48.6)  239 (20.8)  162 (14.1) 190 (16.5)  0.1 [0.0 – 0.5] 672 (58.4)  280 (24.3)  115 (10.0) 83 (7.2)  0.0 [0.0 – 0.0] 1,032 (89.7)  93 (8.1)  20 (1.7) 5 (0.4) | 0.9 [0.1 – 1.9]  439 (16.1)  833 (30.6)  544 (20.0) 905 (33.3)  0.3 [0.0 – 1.2]  1070 (39.3)  669 (24.6)  443 (16.3) 539 (19.8)  0.1 [0.0 – 0.5] 1,580 (58.1)  692 (25.4)  269 (9.9) 180 (6.6)  0.0 [0.0 – 0.0] 2,443 (89.8)  231 (8.5)  35 (1.3) 12 (0.4) | 0/226 | 0.7 [0.1 – 1.7]  81 (16.1)  181 (35.9)  98 (19.4) 144 (28.6)  0.1 [0.0 – 0.9]  261 (51.8)  97 (19.2)  66 (13.1) 80 (15.9)  0.1 [0.0 – 0.6] 276 (54.8)  131 (26.0)  54 (10.7) 43 (8.5)  0.0 [0.0 – 0.0] 459 (91.1)  35 (6.9)  10 (2.0) 0 (0.0) | 0.9 [0.2 – 1.9]  329 (15.7)  640 (30.6)  418 (20.0) 703 (33.6)  0.3 [0.0 – 1.2]  791 (37.8)  519 (24.8)  349 (16.7) 431 (20.6)  0.1 [0.0 – 0.4] 1,227 (58.7)  543 (26.0)  192 (9.2) 128 (6.1)  0.0 [0.0 – 0.0] 1,877 (89.8)  175 (8.4)  28 (1.3) 10 (0.5) | 0/226 | 0.8 [0.1 – 1.8]  191 (16.8)  374 (33.0)  224 (19.7) 346 (30.5)  0.2 [0.0 – 1.0]  540 (47.6)  247 (21.8)  160 (14.1) 188 (16.6)  0.1 [0.0 – 0.6] 629 (55.4)  280 (24.7)  131 (11.5) 95 (8.4)  0.0 [0.0 – 0.0] 1,025 (90.3)  91 (8.0)  17 (1.5) 2 (0.2) |
| **Endpoints** |  |  |  |  |  |  |  |  |  |
| CSVD features  White matter hyperintensity volume, ml  Participants with cerebral microbleeds  Number of cerebral microbleeds  Number of participants with lacunar infarcts  Number of lacunar infarcts | 0.0 [0.0 – 0.01]  245 (11.8)  0.0 [0.0-0.0]  114 (5.5)  0.0 [0.0-0.0] | 0/1,194 | 0.0 [0.0 – 0.01]  26 (14.3)  0.0 [0.0-0.0]  10 (4.4)  0.0 [0.0-0.0] | -  -  - | - | -  -  - | -  -  - | - | -  -  - |
| Retinal microvascular diameters  Arteriolar diameter, MU  Venular diameter, MU | -  - | - | -  - | 142.3 ± 20.2  214.6 ± 31.4 | 0/493 | 140.7 ± 122.9  214.0 ± 31.9 | -  - | - | -  - |
| Flicker light-induced increase in retinal microvascular diameters  Arteriolar, MU  Venular, MU | -  - | - | -  - | -  - | - | -  - | -  4.4 ± 3.6  7.6 ± 4.1 | 0/1,161 | -  3.8 ± 3.5  7.5 ± 3.9 |

Data are presented as mean ± standard deviation, median [interquartile range] or n(%).

Abbreviations: CSVD, cerebral small vessel disease; eGFR, estimated glomerular filtration rate; HbA1c, glycated hemoglobin; HDL, high-density lipoprotein; SD, standard deviation; MVD, microvascular dysfunction; MU, measurement units.

**Supplemental Table S2 Characteristics of the study population and individuals excluded from the analyses due to missing values for the heat-induced skin hyperemia, UAE, and plasma biomarkers of MVD study populations**

|  | Heat-induced skin hyperemia | | | UAE | | | Plasma biomarkers of MVD | | |
| --- | --- | --- | --- | --- | --- | --- | --- | --- | --- |
|  | Complete (n=1,517) | Number of participants with missing data in complete / missing | Missing (n=1,934) | Complete  (n=3,107) | Number of participants with missing data in complete / missing | Missing (n=344) | Complete (n=3,078) | Number of participants with missing data in complete / missing | Missing (n=373) |
| **Demographics** |  |  |  |  |  |  |  |  |  |
| Age, years | 60.2 ± 8.1 | 0/0 | 59.4 ± 8.4 | 59.9 ± 8.2 | 0/0 | 58.5 ± 8.9 | 59.9 ± 8.2 | 0/0 | 58.8 ± 9.0 |
| Men | 786 (51.8) | 0/0 | 989 (51.1) | 1,582 (50.9) | 0/0 | 193 (56.1) | 1,577 (51.2) | 0/0 | 198 (53.1) |
| **Lifestyle factors** |  |  |  |  |  |  |  |  |  |
| Smoking status:  Never  Former  Current | 500 (33.0)  841 (55.4)  176 (11.6) | 0/50 | 672 (35.7)  918 (48.7)  294 (15.6) | 1,088 (35.0)  1,624 (52.3)  395 (12.7) | 0/50 | 84 (28.6)  135 (45.9)  75 (25.5) | 1,075 (34.9)  1,612 (52.4)  391 (12.7) | 0/50 | 97 (30.0)  147 (45.5)  79 (24.5) |
| Body mass index, kg/m^2^ | 27.0 ± 4.4 | 1/2 | 27.1 ± 4.6 | 27.0 ± 4.5 | 1/2 | 27.6 ± 5.1 | 27.1 ± 4.5 | 1/2 | 27.4 ± 4.9 |
| Waist circumference, cm | 96.0 ± 13.4 | 0/4 | 95.9 ± 14.1 | 95.8 ± 13.7 | 0/4 | 97.6 ± 14.7 | 95.8 ± 13.7 | 0/4 | 97.0 ± 14.2 |
| Physical activity, hours/day | 2.0 ± 0.7 | 250/556 | 2.0 ± 0.7 | 2.0 ± 0.7 | 699/107 | 2.0 ± 0.7 | 2.0 ± 0.7 | 686/120 | 1.9 ± 0.7 |
| Dutch Healthy diet score, points | 83.1 ± 14.6 | 0/226 | 83.4 ± 14.8 | 83.2 ± 14.7 | 0/226 | 83.6 ± 14.8 | 83.2 ± 14.7 | 0/226 | 83.7 ± 14.2 |
| **Cardiovascular risk factors** |  |  |  |  |  |  |  |  |  |
| Glucose metabolism status  Normal glucose metabolism  Prediabetes  Type 2 diabetes  Other types of diabetes | 825 (54.4)  230 (15.2)  439 (28.9) 23 (1.5) | 0/0 | 1,099 (56.8)  281 (14.5)  536 (27.7) 18 (0.9) | 1,760 (56.6)  460 (14.8)  854 (27.5) 33 (1.1) | 0/0 | 164 (47.7)  51 (14.8)  121 (35.2) 8 (2.3) | 1,742 (56.6)  455 (14.8)  849 (27.6) 32 (1.0) | 0/0 | 182 (48.8)  56 (15.0)  126 (33.8) 9 (2.4) |
| Fasting plasma glucose, mmol/l | 5.6 [5.1 – 6.6] | 0/1 | 5.6 [5.1 – 6.5] | 5.5 [5.1 – 6.5] | 1/0 | 5.7 [5.1 – 7.1] | 5.5 [5.1 – 6.5] | 1/0 | 5.6 [5.0 – 7.0] |
| 2- hour post load plasma glucose, mmol/l | 6.3 [5.1 – 9.8] | 122/170 | 6.3 [5.1 – 9.0] | 9.3 [6.3 – 9.3] | 239/53 | 6.4 [5.0 – 9.6] | 6.3 [5.1 – 9.2] | 240/52 | 6.5 [5.0 – 9.8] |
| HbA1c, % | 5.7 [5.4 – 6.3] | 1/12 | 5.6 [5.3 – 6.2] | 5.7 [5.4 – 6.2] | 7/6 | 5.8 [5.5 – 6.5] | 5.7 [5.4 – 6.2] | 8/5 | 5.8 [5.5 – 6.4] |
| Use of glucose-lowering medication | 366 (24.1) | 0/0 | 441 (22.8) | 697 (22.4) | 0/0 | 110 (32.0) | 694 (22.5) | 0/0 | 113 (30.3) |
| Total/HDL cholesterol ratio | 3.7 ± 1.1 | 0/4 | 3.7 ± 1.2 | 3.7 ± 1.2 | 0/4 | 3.8 ± 1.2 | 3.7 ± 1.2 | 0/4 | 3.8 ± 1.2 |
| Use of lipid-modifying medication | 592 (39.0) | 0/0 | 667 (34.5) | 1,137 (36.6) | 0/0 | 122 (35.5) | 1,128 (36.6) | 0/0 | 131 (35.1) |
| Office systolic blood pressure, mm Hg | 135.8 ± 18.3 | 0/2 | 134.5 ± 18.1 | 135.0 ± 18.3 | 0/2 | 135.3 ± 17.6 | 135.0 ± 18.2 | 0/2 | 135.5 ± 18.0 |
| Office diastolic blood pressure, mm Hg | 76.5 ± 9.6 | 0/2 | 75.9 ± 10.0 | 76.1 ± 9.9 | 0/2 | 76.3 ± 9.2 | 76.1 ± 9.9 | 0/2 | 76.1 ± 9.0 |
| Ambulatory systolic blood pressure, mm Hg | 119.5 ± 11.4 | 183/2 | 118.7 ± 12.2 | 118.9 ± 11.7 | 349/55 | 120.9 ± 13.2 | 119.0 ± 11.7 | 348/56 | 120.0 ± 12.7 |
| Ambulatory diastolic blood pressure, mm Hg | 73.5 ± 6.9 | 183/2 | 73.5 ± 7.4 | 73.4 ± 7.1 | 349/55 | 74.6 ± 7.2 | 73.4 ± 7.2 | 348/56 | 74.2 ± 7.2 |
| Use of antihypertensive medication | 638 (42.1) | 0/0 | 754 (39.0) | 1,252 (40.3) | 0/0 | 140 (40.7) | 1,246 (40.5) | 0/0 | 146 (39.1) |
| History of cardiovascular disease | 265 (17.5) | 0/67 | 303 (16.2) | 522 (16.8) | 0/67 | 46 (16.6) | 519 (16.9) | 0/67 | 49 (16.0) |
| Diabetic retinopathy | 22 (1.6) | 144/450 | 24 (1.6) | 41 (1.6) | 497/97 | 5 (2.0) | 42 (1.6) | 488/106 | 4 (1.5) |
| eGFR, ml/min/1.73^2^ | 88.1 ± 14.6 | 10/23 | 88.1 ± 15.2 | 88.0 ± 14.9 | 25/8 | 89.5 ± 15.3 | 88.0 ± 14.9 | 7/26 | 89.2 ± 15.3 |
| Plasma biomarkers of low-grade inflammation  C-reactive protein, µg/ml  Serum amyloid A, µg/ml  Tumor necrosis factor alpha, pg/ml  Interleukin-6, pg/ml  Interleukin-8, pg/ml | 1.2 [0.6 – 2.7]  3.3 [2.1 – 5.5]  2.2 [1.9 – 2.6]  0.6 [0.4 – 0.9]  4.1 [3.3 – 5.3] | 11/28 | 1.3 [0.6 – 2.9]  3.2 [2.0 – 5.5]  2.2 [1.9 – 2.6]  0.6 [0.4 – 0.9]  4.2 [3.3 – 5.4] | 1.2 [6.1 – 2.8]  3.3 [2.1 – 5.4]  2.2 [1.9 – 2.6]  4.1 [3.3 – 5.3]  4.1 [3.3 – 5.3] | 28/11 | 1.3 [0.6 – 3.1]  3.1 [2.0 – 5.5]  2.2 [1.9 – 2.6]  0.6 [0.4 – 1.0]  4.3 [3.3 – 5.6] | 1.2 [0.6 – 2.8]  3.2 [2.0 – 5.5]  2.2 [1.9 – 2.6]  0.6 [0.4 – 0.9]  4.1 [3.3 – 5.3] | 1/38 | 1.4 [0.6 – 3.1]  3.1 [2.0 – 5.4]  2.2 [1.9 – 2.6]  0.6 [0.4 – 1.0]  4.3 [3.3 – 5.5] |
| **Other** |  |  |  |  |  |  |  |  |  |
| Educational status  Low   Middle  High | 504 (33.2) 428 (28.2) 585 (38.6) | 0/74 | 631 (33.9) 526 (28.3) 703 (37.8) | 1,041 (33.5)  877 (28.2)  1,189 (38.3) | 0/74 | 94 (34.8)  77 (28.5)  99 (36.7) | 1,027 (33.4) 867 (28.2) 1,184 (38.5) | 0/74 | 108 (36.1) 87 (29.1) 104 (34.8) |
| Occupational status  Low   Middle  High | 412 (32.5) 432 (34.1) 423 (33.4) | 250/377 | 481 (30.9) 563 (36.2) 513 (32.9) | 801 (31.0)  922 (35.7)  860 (33.3) | 524/103 | 92 (38.2)  73 (30.3)  76 (31.5) | 797 (31.1) 908 (35.5) 854 (33.4) | 519/108 | 96 (36.2) 87 (32.8) 82 (30.9) |
| Income per month, euros | 2,029 ± 813 | 353/515 | 2,002 ± 824 | 2,028 ± 818 | 739/129 | 1,871 ± 819 | 2,028 ± 821 | 734/134 | 1,886 ± 787 |
| **Alcohol consumption** |  |  |  |  |  |  |  |  |  |
| Total alcohol consumption, units/day  None  Light  Moderate  High  Total wine consumption, units/day  None  Light  Moderate  High  Total beer consumption, units/day  None  Light  Moderate  High  Total spirits consumption, units /day  None  Light  Moderate  High | 0.9 [0.1 – 1.9]  239 (15.8)  459 (30.3)  313 (20.6) 506 (33.4)  0.3 [0.0 – 1.2]  628 (41.4)  338 (22.3)  248 (16.3) 303 (20.0)  0.1 [0.0 – 0.5] 864 (57.0)  390 (25.7)  159 (10.5) 104 (6.9)  0.0 [0.0 – 0.0] 1358 (89.5)  133 (8.8)  17 (1.1) 9 (0.6) | 0/226 | 0.8 [0.1 – 1.8]  281 (16.5)  555 (32.5)  329 (19.3) 543 (31.8)  0.3 [0.0 – 1.1]  703 (41.2)  428 (25.1)  261 (15.3) 316 (18.5)  0.1 [0.0 – 0.5] 992 (58.1)  433 (25.4)  164 (9.6) 119 (7.0)  0.0 [0.0 – 0.0] 1544 (90.4)  133 (7.8)  28 (1.6) 3 (0.2) | 0.9 [0.1 – 1.9]  498 (16.0)  964 (31.0)  620 (20.0) 1025 (33.0)  0.3 [0.0 – 1.1]  1265 (40.7)  743 (23.9)  497 (16.0) 602 (19.4)  0.1 [0.0 – 0.5] 1775 (57.1)  802 (25.8)  312 (10.0) 218 (7.0)  0.0 [0.0 – 0.0] 2789 (89.8)  262 (8.4)  44(1.4) 12 (0.4) | 0/226 | 0.4 [0.0 – 1.3]  22 (18.6)  50 (42.4)  22 (18.6) 24 (20.3)  0.1 [0.0 – 0.6]  66 (55.9)  23 (19.5)  12 (10.2) 17 (14.4)  0.0 [0.0 – 0.3] 81 (68.6)  21 (17.8)  11 (9.3) 5 (4.2)  0.0 [0.0 – 0.0] 113 (95.8)  4 (3.4)  1 (0.8) 0 (0.0) | 0.9 [0.1 – 1.9]  498 (16.2)  951 (30.9)  615 (20.0) 1014 (32.9)  0.3 [0.0 – 1.1]  1253 (40.7)  734 (23.8)  495 (16.1) 596 (19.4)  0.1 [0.0 – 0.5] 1763 (57.3)  793 (25.8)  309 (10.0) 213 (6.9)  0.0 [0.0 – 0.0] 2764 (89.8)  260 (8.4)  42 (1.4) 12 (0.4) | 0/226 | 0.5 [0.1 – 1.4]  22 (15.0)  63 (42.9)  27 (18.4) 35 (23.8)  0.1 [0.0 – 0.6]  78 (53.1)  32 (21.8)  14 (9.5) 23 (15.6)  0.0 [0.0 – 0.4] 93 (63.3)  30 (20.4)  14 (9.5) 10 (6.8)  0.0 [0.0 – 0.0] 138 (93.9)  6 (4.1)  3 (2.0) (0.0) |
| **Endpoints** |  |  |  |  |  |  |  |  |  |
| Heat-induced skin hyperemia, PU | 112.1 ± 57.3 | 0/1,775 | 109.9 ± 55.8 | - | - | - | - | - | - |
| Urinary albumin excretion, mg/24 hours  ≥30 mg/24h | -  - | - | -  - | 6.7 [4.0 – 11.9]  270 (18.7) | 0/42 | 7.2 [4.3 – 12.9]  29 (9.6) | -  - | - | -  - |
| Plasma biomarkers of MVD  sICAM-1, ng/ml  sVCAM-1, ng/ml  sE-selectin, ng/ml  vWF, % | -  -  -  - | - | -  -  -  - | -  -  -  - | - | -  -  -  - | -  353.9 ± 99.8  428.1 ± 101.0  117.8 ± 65.7  132.6 ± 48.4 | 0/40 | -  365.0 ± 98.8  433.0 ± 108.2  125.6 ± 59.6  135.9 ± 49.3 |

Data are presented as mean ± standard deviation, median [interquartile range] or n(%).

Abbreviations: HDL, high-density lipoprotein; eGFR, estimated glomerular filtration rate; HbA1c, glycated hemoglobin; SD, standard deviation; MVD, microvascular dysfunction; PU, perfusion units; sICAM-1, soluble intercellular adhesin molecule-1; sVCAM-1, soluble vascular adhesion molecule-1; sE-selectin, soluble E-selectin; vWF, von Willebrand factor; UAE, urinary albumin excretion.

**Supplemental Table S3 P-values from likelihood ratio tests for interaction**

|  | Main analyses | | Additional analyses | | | | | | |
| --- | --- | --- | --- | --- | --- | --- | --- | --- | --- |
|  | History of cardiovascular disease status | Sex | Glucose metabolism status | Hypertension status | Smoking status | Dyslipidemia status | Fasting plasma glucose | 2-hour post load glucose | HbA1c |
|  | Total alcohol consumption  entered as a continuous/ categorical variable(s) | Total alcohol consumption  entered as a continuous/ categorical variable(s) | Total alcohol consumption  entered as a continuous/ categorical variable(s) | Total alcohol consumption  entered as a continuous/ categorical variable(s) | Total alcohol consumption  entered as a continuous/ categorical variable(s) | Total alcohol consumption  entered as a continuous/ categorical variable(s) | Total alcohol consumption  entered as a continuous/ categorical variable(s) | Total alcohol consumption  entered as a continuous/ categorical variable(s) | Total alcohol consumption  entered as a continuous/ categorical variable(s) |
| **Determinants** | P-value | P-value | P-value | P-value | P-value | P-value | P-value | P-value | P-value |
| Total alcohol consumption | **<0.001/<0.001** | 0.87/**0.03** | 0.24/0.12 | **<0.001/0.01** | 0.45/0.87 | 0.06/0.21 | NA | NA | NA |
| Individual types of alcohol consumption | **<0.001/<0.001** | 0.60/0.09 | 0.18/**0.02** | 0.33**/<0.001** | 0.24/**0.045** | **0.046/**0.19 | 0.31/0.07 | 0.46/0.08 | 0.69/**0.01** |

P-values are derived from the likelihood ratio test in which the goodness in fit between the model with and without interaction term(s) was tested. A significant P-value (P<0.05) indicates that the models with and without addition of the interaction term statistically significantly differ. Total alcohol consumption was either entered as continuous variable or as a categorical variable (i.e. dummies of none, moderate, or high versus light alcohol consumption).

The following variables were entered in the model in addition to total alcohol consumption or and the interaction term(s) of alcohol consumption with prior cardiovascular disease, blood pressure, glucose metabolism status, continuous measures of glucose, cholesterol, or smoking status : age, sex, glucose metabolism status), educational level, waist circumference, office systolic blood pressure, use of antihypertensive medication, total cholesterol / HDL cholesterol ratio, lipid-modifying medication, smoking status, prior cardiovascular disease, diet score, and, only for heat-induced skin hyperemia, baseline skin blood.

For blood pressure and cholesterol level we entered interaction terms with both blood pressure and antihypertensive or total cholesterol / HDL cholesterol ratio and lipid-modifying medication, respectively. Individuals with other types of diabetes than type 2 diabetes were excluded when interaction terms with glucose metabolism status or continuous measures of glycemia were tested. Interaction by continuous measures of glycemia was only investigated if glucose metabolism status modified an association (in these additional analyses glucose metabolism status was not entered in the model but replaced with the applicable continuous measure of glycemia).

Bold denotes P<0.05.

Abbreviations: NA, not applicable; CI: confidence interval; SD: standard deviation; HbA1c, glycated hemoglobin; HDL, high-density lipoprotein.

**Supplemental Table S4 Associations of alcohol consumption and the total microvascular dysfunction composite score, stratified by history of cardiovascular disease**

|  | Alcohol consumption | | | | | |
| --- | --- | --- | --- | --- | --- | --- |
|  | Model | Continuous | None vs. light | Moderate vs. light | High vs. light | P for trend |
|  |  | β (95% CI) | β (95% CI) | β (95% CI) | β (95% CI) | P- value |
| **With a history of cardiovascular disease, n=525** | | | | | | |
| Total alcohol consumption | 1  2  3A  3B | **-0.12 (-0.18; -0.06)**  **-0.15 (-0.21; -0.09)**  **-0.15 (-0.21; -0.09)**  **-0.14 (-0.20; -0.08)** | **0.40 (0.17; 0.62)**  **0.23 (0.02; 0.45)**  0.21 (-0.01; 0.42)  0.17 (-0.05; 0.39) | -0.09 (-0.33; 0.14)  **-0.23 (-0.45; -0.01)**  -0.21 (-0.43; 0.00)  -**0.22 (-0.44; -0.01)** | **-0.35 (-0.57; -0.13)**  **-0.35 (-0.56; -0.15)**  **-0.35 (-0.55; -0.15)**  -**0.36 (-0.56; -0.16)** | **0.00**  **0.00**  **0.00**  **0.00** |
| Wine consumption | 1  2  3A  3B | **-0.23 (-0.32; -0.14)**  **-0.18 (-0.27; -0.09)**  **-0.17 (-0.26; -0.08)**  **-0.16 (-0.25; -0.08)** | **0.42 (0.22; 0.63)**  0.22 (0.02; 0.43)  0.16 (-0.04; 0.36)  0.14 (-0.06; 0.34) | -0.25 (-0.54; 0.04)  **-0.28 (-0.54; -0.01)**  **-0.27 (-0.54; -0.01)**  **-0.29 (-0.56; -0.03)** | -0.28 (-0.56; 0.01)  -0.22 (-0.49; 0.05)  -0.24 (-0.50; 0.03)  -0.23 (-0.49; 0.04) | **0.00**  **0.00**  **0.00**  **0.00** |
| Beer consumption | 1  2  3A  3B | -0.06 (-0.15; 0.02)  **-0.14 (-0.22; -0.05)**  **-0.15 (-0.23; -0.07)**  **-0.14 (-0.22; -0.06)** | -0.10 (-0.30; 0.10)  0.06 (-0.14; 0.25)  0.06 (-0.13; 0.25)  0.05 (-0.14; 0.24) | -0.19 (-0.50; 0.12)  -0.23 (-0.52; 0.05)  -0.27 (-0.54; 0.01)  -0.28 (-0.55; 0.00) | -0.39 (-0.80; 0.01)  **-0.42 (-0.79; -0.06)**  **-0.50 (-0.86; -0.14)**  -0.48 (-0.84; 0.12) | 0.52  **0.02**  **0.00**  **0.01** |
| Spirits consumption | 1  2  3A  3B | 0.14 (-0.18; 0.45)  0.02 (-0.27; 0.31)  0.02 (-0.26; 0.30)  -0.00 (-0.28; 0.28) | -0.11 (-0.32; 0.10)  0.02 (-0.18; 0.22)  0.03 (-0.17; 0.23)  0.02 (-0.17; 0.22) | -0.19 (-0.53; 0.15)  -0.15 (-0.46; 0.16)  -0.20 (-0.50; 0.11)  -0.21 (-0.51; 0.10) | -0.35 (-0.99; 0.30)  -0.13 (-0.72; 0.45)  -0.24 (-0.82; 0.34)  -0.24 (-0.82; 0.34) | 0.94  0.50  0.39  0.32 |
| **Without a history of cardiovascular disease, n=2,595** | | | | | | |
| Total alcohol consumption | 1  2  3A  3B | 0.01 (-0.02; 0.04)  -0.04 (-0.04; -0.01)  -0.02 (-0.05; 0.01)  -0.02 (-0.04; 0.01) | 0.11 (-0.01; 0.23)  0.08 (-0.04; 0.19)  0.07 (-0.05; 0.18)  0.06 (-0.06; 0.17) | **-0.15 (-0.26; -0.04)**  **-0.11 (-0.21; -0.01)**  -0.09 (-0.19; 0.01)  -0.09 (-0.19; 0.01) | -0.09 (-0.18; 0.01)  -0.08 (-0.17; 0.01)  -0.09 (-0.17; 0.00)  -0.08 (-0.16; 0.01) | **0.00**  **0.00**  **0.00**  **0.01** |
| Wine consumption | 1  2  3A  3B | **-0.07 (-0.11; -0.03)**  **-0.07 (-0.11; -0.03)**  **-0.07 (-0.10; -0.03)**  **-0.06 (-0.10; -0.02)** | **0.16 (0.06; 0.26)**  **0.10 (0.01; 0.20)**  0.07 (-0.02; 0.17)  0.07 (-0.03; 0.16) | **-0.18 (-0.30; -0.06)**  **-0.16 (-0.28; -0.05)**  **-0.16 (-0.27; -0.05)**  **-0.15 (-0.27; -0.04)** | -0.07 (-0.19; 0.04)  -0.05 (-0.16; 0.06)  -0.05 (-0.16; 0.06)  -0.04 (-0.15; 0.07) | **0.00**  **0.00**  **0.00**  **0.00** |
| Beer consumption | 1  2  3A  3B | **0.08 (0.04; 0.12)**  0.04 (0.00; 0.08)  0.03 (-0.01; 0.07)  0.03 (-0.01; 0.07) | -0.08 (-0.17; 0.01)  -0.04 (-0.13; 0.06)  -0.04 (-0.13; 0.05)  -0.04 (-0.13; 0.05) | -0.13 (-0.27; 0.01)  -0.10 (-0.23; 0.03)  -0.12 (-0.25; 0.01)  -0.12 (-0.25; 0.01) | 0.13 (-0.03; 0.29)  0.06 (-0.09; 0.20)  0.03 (-0.12; 0.17)  0.03 (-0.12; 0.18) | 0.11  0.56  0.87  0.77 |
| Spirits consumption | 1  2  3A  3B | **0.19 (0.04; 0.34)**  0.08 (-0.06; 0.22)  0.03 (-0.11; 0.17)  0.02 (-0.12; 0.16) | -0.03 (-0.13; 0.07)  -0.01 (-0.11; 0.08)  -0.02 (-0.11; 0.08)  -0.02 (-0.11; 0.08) | **-0.26 (-0.42; -0.10)**  **-0.19 (-0.33; -0.04)**  **-0.20 (-0.34; -0.05)**  **-0.19 (-0.34; -0.05)** | **-0.30 (-0.58; -0.03)**  -0.22 (-0.48; 0.04)  -0.21 (-0.46; 0.04)  -0.20 (-0.46; 0.05) | **0.00**  0.12  0.55  0.69 |

Betas and 95% confidence intervals indicate the strength of the association between total alcohol, wine, beer, and spirits consumption with the total MVD composite score where a negative beta indicates less MVD. Total alcohol, wine, beer, and spirits consumption were entered in the models as a continuous variable (per unit, i.e. 10 g/day), as dummies (none, moderate or high versus light alcohol consumption) or (for the P-for trend analyses) as a categorical variable (none, light, moderate, and high alcohol consumption).

For individuals with and without a history of cardiovascular disease, respectively, 1 SD corresponds with 1.6 and 1.6 ml white matter hyperintensity volume; 3.1 and 2.3 cerebral microbleeds; 2.5 and 1.5 lacunar infarcts (all logarithmically transformed and standardized per stratum and summed in to the composite score for CSVD features, where the numbers of participants per stratum respectively are n=253 and n=1,822); 21.1 and 20.1 MU of CRAE; 32.8 and 31.1 MU of CRVE (per stratum summed in to the composite score for retinal diameters, where the numbers of participants per stratum respectively are n=459 and n=2,262); 3.7 and 3.5 MU of flicker light-induced increase in retinal arteriolar diameter and 4.3 and 4.1 MU of flicker light-induced increase in retinal venular diameter (per stratum summed in to the composite score for flicker light-induced increase in retinal diameters, where the numbers of participants per stratum respectively are n=330 and n=1,760); 54.2 PU and 57.7 PU of heat-induced skin-hyperemia (where the numbers of participants per stratum respectively are n=265 and n=1,252); 1.16 and 0.93 mg/24 hours of logarithmically transformed UAE (where the numbers of participants per stratum respectively are n=522 and n=2,585); or 122.2 and 93.9 ng/ml sICAM-1; 119.8 and 95.4 ng/ml of sVCAM-1; 91.1 ng/ml and 58.8 ng/ml of sE-selectin; or 51.4 and 47.2% vWF (per stratum summed in to the composite score for plasma biomarkers of MVD, where the numbers of participants per stratum respectively are n=519 and n=2,559).

Model 1: crude; Model 2: age, sex, glucose metabolism status (entered as dummies of type 2 diabetes, prediabetes, or other types of diabetes versus normal glucose metabolism status), educational level [low, middle, high]; model 3A: model 2 + waist circumference, smoking status [current, ever, never], diet score; model 3B: model 3A+ office systolic blood pressure, use of antihypertensive medication [yes/no] total cholesterol / HDL cholesterol ratio, lipid-modifying medication.

Bold denotes P-value<0.05.

Abbreviations: CI: confidence interval; CSVD, cerebral small vessel disease; CRAE, central retina arteriolar equivalent; CRVE, central retinal venular equivalent; SD: standard deviation; MU, measurement units; PU, perfusion units; UAE, urinary albumin excretion; sICAM-1, soluble intercellular adhesion molecule-1; sVCAM-1, soluble vascular adhesion molecule-1; sE-selectin, soluble E-selectin; vWF, von Willebrand factor; MVD, microvascular dysfunction.

**Supplemental Table S5 Associations of total alcohol, wine, beer, and spirits consumption with the total MVD composite score**, stratified by sex

|  |  | | Men | | | | | | Women | | | | | |
| --- | --- | --- | --- | --- | --- | --- | --- | --- | --- | --- | --- | --- | --- | --- |
|  | Model | | Continuous | None vs. light | Moderate vs. light | High vs. light | P for trend | Continuous | | None vs. light | Moderate vs. light | High vs. light | P for trend |  |
|  |  | | β (95% CI) | β (95% CI) | β (95% CI) | β (95% CI) | P-value | β (95% CI) | | β (95% CI) | β (95% CI) | β (95% CI) | P-value |  |
| Total alcohol consumption | 1  2  3A  3B | **-0.05 (-0.08; -0.02)**  **-0.04 (-0.07; -0.01)**  **-0.05 (-0.07; -0.02)**  **-0.04 (-0.06; -0.01)** | | **0.46 (0.29; 0.63)**  **0.30 (0.14; 0.45)**  **0.27 (0.11; 0.42)**  **0.22 (0.07; 0.37)** | -0.12 (-0.25; 0.01)  -0.10 (-0.22; 0.02)  -0.08 (-0.19; 0.04)  -0.09 (-0.20; 0.03) | **-0.15 (-0.27; -0.03)**  **-0.14 (-0.24; -0.03)**  **-0.15 (-0.26; -0.04)**  **-0.13 (-0.23; -0.02)** | **0.00**  **0.00**  **0.00**  **0.00** | **-0.09 (-0.14; -0.03)**  **-0.07 (-0.12; -0.02)**  **-0.08 (-0.13; -0.03)**  **-0.07 (-0.12; -0.01)** | | **0.26 (0.11; 0.40)**  0.11 (-0.03; 0.25)  0.11 (-0.02; 0.25)  0.09 (-0.04; 0.23) | **-0.18 (-0.32; -0.02)**  **-0.16 (-0.30; -0.01)**  -0.13 (-0.27; 0.02)  -0.11 (-0.25; 0.04) | -0.10 (-0.23; 0.03)  -0.12 (-0.25 0.00)  -0.12 (-0.24; 0.00)  -0.10 (-0.22; 0.03) | **0.00**  **0.00**  **0.00**  **0.00** |  |
| Wine | 3B | **-0.06 (-0.11; -0.02)** | | **0.13 (0.03; 0.23)** | **-0.17 (-0.31; -0.04)** | -0.00 (-0.16; 0.15) | **0.00** | **-0.09 (-0.14; -0.01)** | | 0.03 (-0.11; 0.18) | **-0.18 (-0.34; -0.02)** | -0.14 (-0.28; 0.01) | **0.00** |  |
| Beer | 3B | -0.02 (-0.05; 0.02) | | 0.03 (-0.07; 0.13) | -0.11 (-0.24; 0.02) | -0.07 (-0.22; 0.08) | 0.09 | 0.07 (-0.05; 0.19) | | **-0.11 (-0.26; 0.04)** | -0.17 (-0.43; 0.09) | -0.02 (-0.32; 0.29) | 0.40 |  |
| Spirits | 3B | -0.02 (-0.15; 0.11) | | 0.05 (-0.06; 0.15) | -0.15 (-0.30; -0.01) | -0.20 (-0.47; 0.07) | 0.62 | 0.02 (-0.32; 0.37) | | -0.07 (-0.23; 0.09) | -0.23 (-0.51; 0.05) | -0.26 (-0.78; 0.25) | 0.98 |  |

Betas and 95% confidence intervals represent the difference in total MVD composite score (per SD) per unit of total alcohol, wine, beer, or spirits consumption or for none, moderate, or high versus light total alcohol, wine, beer, or spirits consumption where a negative beta indicates less MVD. Total alcohol, wine, beer and spirits consumption were entered in the models as a continuous variable (per unit, i.e. 10 g/day), as dummies (none, moderate or high versus light alcohol consumption) or (for the P-for trend analyses) as a categorical variable (none, light, moderate, and high alcohol consumption). For all analyses the value of one SD was numerically comparable to the value of one SD that was presented in the legend of Table 2. The number of men and women, respectively are n=1,592 and n=1,528.

Model 1: crude; Model 2: age, glucose metabolism status (entered as dummies of type 2 diabetes, prediabetes, or other types of diabetes versus normal glucose metabolism status), educational level [low, middle, high]; model 3A: model 2 + waist circumference, smoking status [current, ever, never], diet score; model 3B: model 3A+ office systolic blood pressure, use of antihypertensive medication [yes/no] total cholesterol / HDL cholesterol ratio, lipid-modifying medication, prior cardiovascular disease.

Bold denotes P-value<0.05.

Abbreviations: β, beta; CI: confidence interval; SD: standard deviation; MVD, microvascular dysfunction; HDL, high-density lipoprotein.

**Supplemental Table S6 Associations of total alcohol, wine, beer, and spirits consumption with the total MVD composite score, stratified by cardiovascular risk factor status**

|  | Alcohol consumption | | | | | |
| --- | --- | --- | --- | --- | --- | --- |
|  | Model | Continuous | None vs. light | Moderate vs. light | High vs. light | P for trend |
|  |  | β (95% CI) | β (95% CI) | β (95% CI) | β (95% CI) | P- value |
| **With type 2 diabetes, n=857** |  |  |  |  |  |  |
| - Total alcohol consumption | 1  2  3A  3B | **-0.05 (-0.10; -0.01)**  **-0.09 (-0.13; -0.04)**  **-0.08 (-0.13; -0.04)**  **-0.07 (-0.11; -0.02)** | 0.04 (-0.13; 0.21)  0.17 (-0.01; 0.34)  0.15 (-0.03; 0.32)  0.09 (-0.08; 0.26) | **-0.21 (-0.41; -0.01)**  **-0.20 (-0.40; -0.00)**  -0.16 (-0.35; 0.03)  -0.18 (-0.37; 0.01) | **-0.25 (-0.43; -0.07)**  **-0.24 (-0.41; -0.06)**  **-0.23 (-0.40; -0.06)**  **-0.20 (-0.37; -0.04)** | **0.00**  **0.00**  **0.00**  **0.00** |
| - Wine - Beer - Spirits | 3B  3B  3B | **-0.11 (-0.18; -0.03)**  -0.04 (-0.10; 0.02)  -0.01 (-0.24; 0.21) | 0.08 (-0.08; 0.25)  0.15 (-0.01; 0.32)  **0.17 (0.00; 0.33)** | **-0.35 (-0.59; -0.12)**  -0.03 (-0.28; 0.21)  -0.08 (-0.34; 0.19) | 0.01 (-0.23; 0.26)  -0.09 (-0.33; 0.16)  -0.22 (-0.63; 0.20) | **0.03**  **0.02**  0.94 |
| **With prediabetes, n=461** |  |  |  |  |  |  |
| - Total alcohol consumption | 1  2  3A  3B | -0.02 (-0.08; 0.03)  -0.06 (-0.11; 0.00)  -0.06 (-0.11; 0.00)  -0.06 (-0.12; 0.00) | 0.31 (0.00; 0.61)  **0.43 (0.12; 0.73)**  **0.42 (0.12; 0.72)**  **0.38 (0.08; 0.68)** | 0.00 (-0.27;0.26)  -0.08 (-0.34; 0.18)  -0.07 (-0.33; 0.19)  -0.11 (-0.37; 0.15) | -0.10 (-0.32; 0.12)  -0.18 (-0.40; 0.04)  -0.17 (-0.39; 0.05)  -0.19 (-0.41; 0.03) | **0.02**  **0.00**  **0.00**  **0.00** |
| - Wine - Beer - Spirits | 3B  3B  3B | **-0.12 (-0.20; -0.04)**  0.02 (-0.07; 0.10)  -0.16 (-0.48; 0.16) | **0.31 (0.08; 0.54)**  **-0.25 (-0.48; -0.02)**  -0.21 (-0.44; 0.03) | -0.15 (-0.45; 0.14)  -0.44 (-0.74; 0.13)  -0.59 (-0.92; 0.26) | -0.11 (-0.38; 0.16)  -0.30 (-0.64; 0.04)  **-0.77 (-1.29; -0.25)** | **0.00**  0.63  **0.13** |
| **With normal glucose metabolism, n=1,769** | | | | | | |
| - Total alcohol consumption | 1  2  3A  3B | 0.03 (-0.01; 0.07)  -0.02 (-0.06; 0.02)  -0.03 (-0.07; 0.01)  -0.01 (-0.05; 0.03) | 0.02 (-0.14; 0.18)  0.07 (-0.09; 0.22)  0.06 (-0.10; 0.22)  0.04 (-0.11; 0.20) | -0.09 (-0.22; 0.04)  **-0.14 (-0.27; -0.01)**  -0.12 (-0.25; 0.01)  **-**0.10 (-0.22; 0.03) | -0.03 (-0.15; 0.08)  -0.10 (-0.22; 0.01)  -0.11 (-0.23; 0.00)  -0.08 (-0.20; 0.03) | 0.40  **0.01**  **0.01**  0.05 |
| - Wine - Beer - Spirits | 3B  3B  3B | -0.05 (-0.10; 0.00)  0.03 (-0.03; 0.08)  0.07 (-0.13; 0.26) | 0.03 (-0.09; 0.15)  **-0.12 (0.24; 0.00)**  -0.12 (0.25; -0.00) | **-0.15 (-0.28; -0.01)**  -0.15 (-0.32; 0.01)  -0.15 (-0.34; 0.04) | -0.08 (-0.21; 0.06)  0.05 (-0.16; 0.26)  0.06 (-0.31; 0.43) | **0.02**  0.22  0.65 |
| **With hypertension, n=1,792** |  |  |  |  |  |  |
| - Total alcohol consumption | 1  2  3A  3B | **-0.05 (-0.08; -0.02)**  **-0.06 (-0.09; -0.03)**  **-0.06 (-0.09; -0.03)**  **-0.05 (-0.08; -0.02)** | **0.22 (0.08; 0.35)**  **0.21 (0.08; 0.34)**  **0.19 (0.06; 0.32)**  **0.15 (0.02; 0.28)** | **-0.14 (-0.27; -0.00)**  -0.09 (-0.21; 0.03)  -0.06 (-0.18; 0.06)  -0.06 (-0.18; 0.06) | **-0.23 (-0.35; -0.11)**  **-0.16 (-0.26; -0.05)**  **-0.15 (-0.26; -0.04)**  **-0.13 (-0.24; -0.03)** | **0.00**  **0.00**  **0.00**  **0.00** |
| - Wine - Beer - Spirits | 3B  3B  3B | **-0.08 (-0.13; -0.04)**  -0.03 (-0.07; 0.01)  0.00 (-0.14; 0.15) | **0.17 (0.06; 0.28)**  0.06 (-0.05; 0.17)  0.08 (-0.03; 0.18) | -0.11 (-0.24; 0.03**)**  -0.00 (-0.15; 0.15)  -0.05 (-0.22; 0.11) | 0.01 (-0.13; 0.15)  -0.16 (-0.22; 0.01)  **-0.33 (-0.61; -0.05)** | **0.01**  **0.02**  0.75 |
| **Without hypertension, n=1,328** |  |  |  |  |  |  |
| - Total alcohol consumption | 1  2  3A  3B | 0.01 (-0.03; 0.06)  -0.02 (-0.07; 0.02)  -0.04 (-0.09; 0.01)  -0.03 (-0.08; 0.01) | **0.18 (0.01; 0.35)**  0.11 (-0.06; 0.28)  0.11 (-0.06; 0.28)  0.09 (-0.07; 0.26) | **-0.16 (-0.32; -0.01)**  **-0.18 (-0.33; -0.04)**  **-0.18 (-0.33; -0.03)**  **-0.17 (-0.32; -0.02)** | -0.02 (-0.15; 0.12)  -0.10 (-0.23; 0.04)  -0.12 (-0.25; 0.01)  -0.11 (-0.24; 0.02) | 0.05  **0.01**  **0.00**  **0.01** |
| - Wine - Beer - Spirits | 3B  3B  3B | **-0.08 (-0.14; -0.03)**  0.05 (-0.02; 0.13)  -0.06 (-0.31; 0.19) | -0.02 (-0.16; 0.12)  **-0.15 (-0.29; -0.01)**  **-0.14 (-0.29; 0.00)** | **-0.27 (-0.44; -0.11)**  **-0.34 (-0.54; -0.15)**  **-0.37 (-0.60; -0.14)** | **-0.18 (-0.34; -0.03)**  0.18 (-0.06; 0.43)  0.12 (-0.32; 0.56) | **0.00**  0.21  0.89 |
| **With dyslipidemia, n=2,064** |  |  |  |  |  |  |
|  |  |  |  |  |  |  |
| - Total alcohol consumption | 1  2  3A  3B | **-0.05 (-0.08; -0.02)**  **-0.06 (-0.09; -0.03)**  **-0.07 (-0.10; -0.04)**  **-0.06 (-0.09; -0.03)** | **0.20 (0.07; 0.33)**  **0.17 (0.05; 0.29)**  **0.16 (0.04; 0.28)**  0.12 (-0.00; 0.24) | **-0.16 (-0.28; -0.03)**  **-0.12 (-0.23; -0.00)**  -0.10 (-0.21; 0.01)  -0.10 (-0.21; 0.01) | **-0.21 (-0.31; -0.10)**  **-0.15 (-0.25; -0.05)**  **-0.15 (-0.25; -0.05)**  **-0.13 (-0.23; -0.04)** | **0.00**  **0.00**  **0.00**  **0.00** |
| - Wine - Beer - Spirits | 3B  3B  3B | **-0.08 (-0.12; -0.04)**  **-0.04 (-0.09; -0.00)**  0.05 (-0.09; 0.19) | **0.12 (0.02; 0.22)**  0.05 (-0.05; 0.15)  0.05 (-0.05; 0.15) | **-0.15 (-0.27; -0.02)**  -0.07 (-0.21; 0.07)  -0.09 (-0.24; 0.07) | -0.02 (-0.14; 0.11)  -0.15 (-0.21; 0.07)  -0.20 (-0.48; 0.09) | **0.00**  **0.01**  0.42 |
| **Without dyslipidemia, n=1,056** |  |  |  |  |  |  |
| - Total alcohol consumption | 1  2  3A  3B | 0.03 (-0.02; 0.07)  -0.02 (-0.06; 0.03)  -0.02 (-0.07; 0.02)  -0.01 (-0.05; 0.04) | **0.26 (0.06; 0.45)**  0.16 (-0.03; 0.34)  0.15 (-0.03; 0.33)  0.13 (-0.06; 0.31) | -0.12 (-0.29; 0.05)  -0.14 (-0.30; 0.02**)**  -0.11 (-0.27; 0.05)  -0.09 (-0.25; 0.07) | -0.02 (-0.17; 0.13)  -0.10 (-0.24; 0.04)  -0.10 (-0.24; 0.04)  -0.07 (-0.21; 0.07) | **0.02**  **0.01**  **0.01**  **0.04** |
| - Wine - Beer - Spirits | 3B  3B  3B | **-0.08 (-0.14; -0.01)**  **0.07 (0.00; 0.13)**  -0.18 (-0.46; 0.09) | 0.07 (-0.08; 0.22)  -0.16 (-0.30; 0.02)  -0.14 (-0.28; 0.01) | **-0.19 (-0.37; -0.02)**  -0.28 (-0.49; 0.07)  **-0.35 (-0.59; -0.13)** | -0.11 (-0.29; 0.06)  0.12 (-0.12; 0.36)  -0.11 (-0.48; -0.27) | **0.01**  0.12  **0.03** |
| **With current smoking, n=396** |  |  |  |  |  |  |
| - Total alcohol consumption | 1  2  3A  3B | -0.03 (-0.09; 0.04)  -0.04 (-0.10; 0.03)  -0.02 (-0.08; 0.03)  -0.01 (-0.07; 0.05) | 0.24 (-0.04; 0.52)  0.09 (-0.18; 0.36)  0.11 (-0.15; 0.37)  0.11 (-0.14; 0.37) | -0.24 (-0.54; 0.06)  -0.20 (-0.48; 0.08)  -0.15 (-0.41; 0.12)  -0.14 (-0.40; 0.13) | -0.19 (-0.45; 0.06)  -0.19 (-0.42; 0.05)  -0.16 (-0.38; 0.07)  -0.12 (-0.35; 0.11) | **0.00**  **0.02**  **0.02**  0.06 |
| - Wine - Beer - Spirits | 3B  3B  3B | **-0.10 (-0.19; -0.01)**  0.06 (-0.02; 0.14)  -0.07 (-0.31; 0.17) | 0.04 (-0.20; 0.27)  -0.10 (-0.33; 0.12)  -0.07 (-0.30; 0.15) | **-0.52 (-0.83; -0.20)**  -0.14 (-0.46; 0.18)  -0.23 (-0.57; 0.11) | -0.12 (-0.41; 0.17)  0.07 (-0.25; 0.38)  -0.24 (-0.75; 0.27) | **0.03**  0.35  0.40 |
| **Without current smoking, n=2,724** |  |  |  |  |  |  |
| - Total alcohol consumption | 1  2  3A  3B | -0.02 (-0.05; 0.00)  **-0.06 (-0.08; -0.03)**  **-0.06 (-0.08; -0.03)**  **-0.05 (-0.08; -0.02)** | **0.20 (0.08; 0.32)**  **0.16 (0.05; 0.26)**  **0.16 (0.05; 0.27)**  0.13 (0.02; 0.24) | -0.14 (-0.24; -0.03)  **-0.12 (-0.22; -0.02)**  -0.10 (-0.19; 0.00)  -0.09 (-0.19; 0.01) | **-0.15 (-0.24; -0.06)**  **-0.13 (-0.22; -0.04)**  -0.13 (-0.22; 0.05)  **-0.11 (-0.20; -0.02)** | **0.00**  **0.00**  **0.00**  **0.00** |
| - Wine - Beer - Spirits | 3B  3B  3B | **-0.07 (-0.11; -0.04)**  -0.03 (-0.07; 0.01)  0.01 (-0.13; 0.15) | **0.11 (0.02; 0.20)**  -0.00 (-0.09; 0.08)  0.00 (-0.09; 0.09) | -0.10 (-0.21; 0.00)  -0.12 (-0.25; 0.00)  -0.13 (-0.27; 0.01) | -0.04 (-0.15; 0.07)  -0.10 (-0.25; 0.05)  -0.14 (-0.39; 0.11) | **0.00**  0.09  0.98 |

Betas and 95% confidence intervals indicate the strength of the association between total alcohol, wine, beer, and spirits consumption with the total MVD composite score where a negative beta indicates less MVD. Total alcohol, wine, beer, and spirits consumption were entered in the models as a continuous variable (per unit, i.e. 10 g/day), as dummies (none, moderate or high versus light alcohol consumption) or (for the P-for trend analyses) as a categorical variable (none, light, moderate, and high alcohol consumption).

For individuals with type 2 diabetes, individuals with prediabetes, and individuals with normal glucose metabolism, respectively, one SD corresponds with 1.6, 1.7 and 1.5 ml white matter hyperintensity volume; 2.6, 2.4 and 2.3 cerebral microbleeds; 2.0, 1.9 and 1.4 lacunar infarcts (all logarithmically transformed and standardized per stratum and summed in to the composite score for CSVD features, where the numbers of participants per stratum respectively are n=459, n=321, and n=1,274); 20.9, 20.2 and 19.9 MU of CRAE; 32.7, 31.5 and 30.5 MU of CRVE (per stratum summed in to the composite score for retinal diameters, where the numbers of participants per stratum respectively are n=758, n=403, and n=1,533); 3.5, 3.4 and 3.6 MU of flicker light-induced increase in retinal arteriolar diameter and 4.2, 4.4 and 4.0 MU of flicker light-induced increase in retinal venular diameter (per stratum summed in to the composite score for flicker light-induced increase in retinal diameters, where the numbers of participants per stratum respectively are n=563, n=307, and n=1,197); 48.8, 53.2, and 60.7 PU of heat-induced skin hyperemia (where the numbers of participants per stratum respectively are n= 439, n=230, n=825); 1.1 mg/24 hours, 0.9 mg/24 hours, 0.81 mg/24 hours of logarithmically transformed UAE (where the numbers of participants per stratum respectively are n= 854, n=460, n=1,760); 127.7, 95.7, and 78.6 ng/ml of sICAM-1; 119.6, 101.1, and 85.6 ng/ml of sVCAM-1; 88.3, 55.3, and 46.8 ng/ml of sE-selectin; and 53.3, 47.8, and 44.2 % vWF (per stratum combined in the plasma biomarkers of MVD composite score, where the numbers of participants per stratum respectively are n= 849, n=455, n=1,742).

For individuals with and without hypertension, respectively, 1 SD corresponds with 1.6 and 1.4 ml white matter hyperintensity volume; 2.6 and 2.1 cerebral microbleeds; 1.8 and 1.4 lacunar infarcts (all logarithmically transformed and standardized per stratum and summed in to the composite score for CSVD features, where the numbers of participants per stratum respectively are n=1,101 and n=974); 20.4 and 19.6 MU of CRAE, 32.3 and 30.0 MU of CRVE (per stratum summed in to the composite score for retinal diameters, where the numbers of participants per stratum respectively are n=1,559 and n=1,162); 3.5 and 3.6 MU of flicker light-induced increase in retinal arteriolar diameter and 4.0 and 4.2 MU of flicker light-induced increase in retinal venular diameter (per stratum summed in to the composite score for flicker light-induced increase in retinal diameters, where the numbers of participants per stratum respectively are n=1,173 and n=917); 55.7 and 58.6 PU of heat-induced skin hyperemia, where the numbers of participants per stratum respectively are n=898 and n=619; and 1.2 and 0.7 mg/24hours of logarithmically transformed UAE, where the numbers of participants per stratum respectively are n=1,788 and n=1,319; or 107.3 and 86.1 ng/ml sICAM-1; 107.5 and 88.3 ng/ml of sVCAM-1; 74.8 ng/ml and 46.7 ng/ml of sE-selectin; or 49.5 and 46.1% vWF (per stratum summed in to the composite score for plasma biomarkers of MVD, where the numbers of participants per stratum respectively are n=1,772 and n=1,306).

For individuals with and without hypercholesterolemia, respectively, 1 SD corresponds with 1.6 and 1.5 ml white matter hyperintensity volume; 2.5 and 2.2 cerebral microbleeds; 1.8 and 1.4 lacunar infarcts (all logarithmically transformed and standardized per stratum and summed in to the composite score for CSVD features, where the numbers of participants per stratum respectively are n=1,301 and n=774); 19.8 and 21.0 MU of CRAE; 31.4 and 31.3 MU of CRVE (per stratum summed in to the composite score for retinal diameters, where the numbers of participants per stratum respectively are n=1,780 and n=941); 3.6 and 3.5 MU of flicker light-induced increase in retinal arteriolar diameter and 4.1 and 4.1 MU of flicker light-induced increase in retinal venular diameter (per stratum summed in to the composite score for flicker light-induced increase in retinal diameters, where the numbers of participants per stratum respectively are n=1350 and n=740); 57.5 and 56.7 PU of heat-induced skin hyperemia, where the numbers of participants per stratum respectively are n=1,055 and n=462; 1.0 and 0.9 mg/24-hours of UAE, where the numbers of participants per stratum respectively are n=2,060 and n=1,047); or 104.1 and 89.7 ng/ml sICAM-1; 101.8 and 99.3 ng/ml of sVCAM-1; 70.7 ng/ml and 52.6 ng/ml of sE-selectin; or 48.3 and 48.4% vWF (per stratum summed in to the composite score for plasma biomarkers of MVD, where the numbers of participants per stratum respectively are n=2,038 and n=1,040).

For current and non-current smokers, respectively, 1 SD corresponds with 1.6 and 1.6 ml white matter hyperintensity volume; 2.3 and 2.4 cerebral microbleeds; 1.8 and 1.6 lacunar infarcts (all logarithmically transformed and standardized per stratum and summed in to the composite score for CSVD features, where the numbers of participants per stratum respectively are n=236 and n=1,839); 20.7 and 20.1 MU of CRAE; 32.5 and 31.0 MU of CRVE (per stratum summed in to the composite score for retinal diameters, where the numbers of participants per stratum respectively are n=333 and n=2,388); 3.6 and 3.6 MU of flicker light-induced increase in retinal arteriolar diameter and 4.8 and 4.0 MU of flicker light-induced increase in retinal venular diameter (per stratum summed in to the composite score for flicker light-induced increase in retinal diameters, where the numbers of participants per stratum respectively are n=249 and n=1,841); 48.7 and 58.1 PU of heat-induced skin hyperemia, where the numbers of participants per stratum respectively are n=176 and n=1341; 1.1 and 1.0 mg/24-hours of UAE, where the numbers of participants per stratum respectively are n=395 and n=2,712; or 127.0 and 93.4 ng/ml sICAM-1; 105.7 and 99.9 ng/ml of sVCAM-1; 66.4 ng/ml and 65.6 ng/ml of sE-selectin; or 48.7 and 48.3% vWF (per stratum summed in to the composite score for plasma biomarkers of MVD, where the numbers of participants per stratum respectively are n=391 and n=2,687).

Model 1: crude; Model 2: age, sex, glucose metabolism status (where applicable; entered as dummies of type 2 diabetes, prediabetes, or other types of diabetes versus normal glucose metabolism status), educational level [low, middle, high]; model 3A: model 2 + waist circumference, smoking status (where applicable; current, ever, never), diet score; model 3B: model 3A+ office systolic blood pressure, use of antihypertensive medication [yes/no] total cholesterol / HDL cholesterol ratio, lipid-modifying medication, prior cardiovascular disease.

Bold denotes P-value<0.05.

Abbreviations: CI: confidence interval; CSVD, cerebral small vessel disease; CRAE, central retina arteriolar equivalent; CRVE, central retinal venular equivalent; SD: standard deviation; MU, measurement units; PU, perfusion units; UAE, urinary albumin excretion; sICAM-1, soluble intercellular adhesion molecule-1; sVCAM-1, soluble vascular adhesion molecule-1; sE-selectin, soluble E-selectin; vWF, von Willebrand factor; MVD, microvascular dysfunction.

**Supplemental Table S7 Associations of total alcohol consumption with the total MVD composite score, stratified by number of cardiovascular risk factors**

|  | Alcohol consumption | | | | | |
| --- | --- | --- | --- | --- | --- | --- |
|  | Model | Continuous | None vs. light | Moderate vs. light | High vs. light | P for trend |
|  |  | β (95% CI) | β (95% CI) | β (95% CI) | β (95% CI) | P- value |
| **Total MVD composite score, per SD** |  |  |  |  |  |  |
| 0 risk factors, n= 450 | 3B | 0.00 (-0.09; 0.09) | 0.12 (-0.19; 0.43) | -0.18 (-0.43; 0.07) | **-**0.11 (-0.34; 0.13) | 0.12 |
| One risk factor  - Type 2 diabetes, n= 857  - Hypertension, n= 1,792  - Dyslipidemia, n=2,064  - Smoking, n= 396 | 3B  3B  3B  3B | **-0.07 (-0.11; 0.02)**  **-0.05 (-0.08; -0.02)**  **-0.06 (-0.09; -0.03)**  -0.01 (-0.07; 0.05) | 0.09 (-0.08; 0.26)  **0.15 (0.02; 0.28)**  0.12 (-0.00; 0.24)  0.11 (-0.14; 0.37) | -0.18 (-0.37; 0.01)  -0.06 (-0.18; 0.06)  -0.10 (-0.21; 0.01)  -0.14 (-0.40; 0.13) | **-0.20 (-0.37; -0.04)**  **-0.13 (-0.24; -0.03)**  **-0.13 (-0.23; -0.04)**  -0.12 (-0.35; 0.11) | **0.00**  **0.00**  **0.00**  0.06 |
| Two risk factors  - Type 2 diabetes and hypertension, n=720  - Type 2 diabetes and dyslipidemia, n=715  - Type 2 diabetes and smoking, n= 132  - Hypertension and dyslipidemia, n= 1,355  - Hypertension and smoking, n= 226  - Dyslipidemia and smoking, n=277 | 3B  3B  3B  3B  3B  3B | **-0.07 (-0.12; -0.03)**  **-0.07 (-0.12; -0.02)**  0.01 (-0.09; 0.12)  **-0.07 (-0.10; -0.03)**  -0.00 (-0.08; 0.08)  -0.03 (-0.10; 0.05) | 0.08 (-0.11; 0.27)  0.05 (-0.15; 0.24)  -0.07 (-0.53; 0.40)  0.13 (-0.02; 0.28)  0.05 (-0.30; 0.39)  0.09 (-0.20; 0.39) | -0.18 (-0.39; 0.03)  -0.16 (-0.37; 0.05)  -0.20 (-0.74; 0.35)  -0.09 (-0.22; 0.05)  -0.10 (-0.46; 0.25)  0.20 (-0.52; 0.11) | **-0.24 (-0.43; -0.06)**  **-0.22 (-0.40; -0.03)**  **-**0.21 (-0.69; 0.27)  **-0.17 (-0.30; -0.05)**  -0.23 (-0.54; 0.07)  -0.26 (-0.54; 0.02) | **0.00**  **0.00**  0.44  **0.00**  0.07  **0.01** |
| Three risk factors  - Type 2 diabetes, hypertension, and dyslipidemia, n=622  - Type 2 diabetes, hypertension, and smoking, n=108  -Hypertension, dyslipidemia, and smoking, n=176 | 3B  3B  3B | **-0.08 (-0.13; -0.03)**  0.00 (-0.12; 0.13)  0.00 (-0.09; 0.09) | 0.02 (-0.18; 0.23)  -0.15 (-0.67; 0.13)  -0.06 (-0.45; 0.34) | -0.19 (-0.41;0.04)  -0.16 (-0.75; 0.42)  -0.16 (-0.61; 0.28) | **-0.28 (-0.47; -0.08)**  -0.27 (-0.79; 0.24)  -0.27 (-0.63; 0.10) | **0.00**  0.51  0.19 |
| Four risk factors, n=97 | 3B | 0.01 (-0.13; 0.15) | -0.28 (0.84; 0.29) | -0.24 (-0.90; 0.42) | -0.34 (-0.90; 0.22) | 0.64 |

Betas and 95% confidence intervals indicate the strength of the association between total alcohol consumption and the total MVD composite score where a negative beta indicates less MVD. Total alcohol consumption was entered in the model as a continuous variable (per unit, i.e. 10 g/day), as dummies (none, moderate or high versus light alcohol consumption) or (for the P-for trend analyses) as a categorical variable (none, light, moderate, and high alcohol consumption). Numerical values per SD in individual with and without a risk factor are presented in the legend of Supplemental Table S6.

Variables in model 3B: age, sex, glucose metabolism status (entered as dummies of type 2 diabetes, prediabetes, or other types of diabetes versus normal glucose metabolism status), educational level [low, middle, high], waist circumference, smoking status (current, ever, never), diet score, office systolic blood pressure, use of antihypertensive medication [yes/no] total cholesterol / HDL cholesterol ratio, lipid-modifying medication, and prior cardiovascular disease.

Bold denotes P-value<0.05.

Abbreviations: CI: confidence interval; SD: standard deviation; MVD, microvascular dysfunction.

**Supplemental Table S8 Associations of total alcohol consumption with the total MVD composite score composed out of five instead of six measures of MVD**

|  | Alcohol consumption | | | | | | |
| --- | --- | --- | --- | --- | --- | --- | --- |
|  | Model | Continuous | None vs. light | Moderate vs. light | High vs. light | P for trend | |
|  |  | β (95% CI) | β (95% CI) | β (95% CI) | β (95% CI) | P- value | |
| **Total MVD composite score, per SD** |  |  |  |  |  |  | |
| - **Without CSVD features** |  |  |  |  |  |  | |
| Total alcohol consumption, n=3,114 | 3B | **-0.04 (-0.07; -0.02)** | **0.11 (0.01; 0.21)** | **-0.11 (-0.20; -0.01)** | **-0.11 (-0.19; -0.03)** | | **0.00** |
| - **Without retinal microvascular diameters** |  |  |  |  |  |  | |
| Total alcohol consumption, n=3,116 | 3B | **-0.04 (-0.07; -0.02)** | **0.12 (0.03; 0.21)** | -0.06 (-0.14; 0.03) | **-0.11 (-0.18; -0.03)** | **0.00** | |
| - **Without flicker light-induced retinal microvascular diameters** |  |  |  |  |  |  | |
| Total alcohol consumption, n=3,120 | 3B | **-0.04 (-0.07; -0.02)** | **0.14 (0.05; 0.24)** | -0.09 (-0.17; 0.00) | **-0.10 (-0.18; -0.02)** | **0.00** | |
| - **Without heat-induced skin hyperemia** |  |  |  |  |  |  | |
| Total alcohol consumption, n=3,120 | 3B | **-0.04 (-0.06; -0.02)** | **0.13 (0.03; 0.23)** | **-0.10 (-0.18; -0.01)** | **-0.11 (-0.19; -0.03)** | **0.00** | |
| - **Without UAE** |  |  |  |  |  |  | |
| Total alcohol consumption, n=3,009 | 3B | **-0.04 (-0.06; -0.02)** | **0.09 (-0.02; 0.19)** | -0.07 (-0.17; 0.02) | **-0.12 (-0.20; -0.03)** | **0.00** | |
| - **Without plasma biomarkers of MVD** |  |  |  |  |  |  | |
| Total alcohol consumption, n=3,017 | 3B | **-0.03 (-0.05; 0.00)** | 0.01 (-0.10; 0.12) | **-0.11 (-0.21; -0.02)** | **-0.11 (-0.19; -0.02)** | **0.01** | |

Betas and 95% confidence intervals indicate the strength of the association between total alcohol consumption and the total MVD composite score where a negative beta indicates less MVD. Total alcohol consumption was entered in the model as a continuous variable (per unit, i.e. 10 g/day), as dummies (none, moderate or high versus light alcohol consumption) or (for the P-for trend analyses) as a categorical variable (none, light, moderate, and high alcohol consumption). Numerical values per SD were similar to the values per SD for the total MVD composite score based on six measures of MVD in the general population, which are presented in the legend of Table 2.

Variables in model 3B: age, sex, glucose metabolism status (entered as dummies of type 2 diabetes, prediabetes, or other types of diabetes versus normal glucose metabolism status), educational level [low, middle, high], waist circumference, smoking status (current, ever, never), diet score, office systolic blood pressure, use of antihypertensive medication [yes/no] total cholesterol / HDL cholesterol ratio, lipid-modifying medication, and prior cardiovascular disease.

Bold denotes P-value<0.05.

Abbreviations: CI: confidence interval; SD: standard deviation; MVD, microvascular dysfunction.

| **Supplemental Table S9 Associations of total alcohol consumption with measures of MVD, in the general population** | | | | | | | |
| --- | --- | --- | --- | --- | --- | --- | --- |
|  |  | | Total alcohol consumption | | | | |
|  | Model | Continuous | | None vs. light | Moderate vs. light | High vs. light | P for trend |
|  |  | β (95% CI) | | β (95% CI) | β (95% CI) | β (95% CI) | P- value |
|  |  |  | |  |  |  |  |
| CSVD features composite score, per SD | 1  2  3A  3B | **0.04 (0.01; 0.07)**  -0.00 (-0.03; 0.03)  -0.01 (-0.04; 0.02)  -0.00 (-0.03; 0.03) | | -0.01 (-0.15; 0.12)  -0.01 (-0.13; 0.12)  -0.00 (-0.13; 0.12)  -0.02 (-0.15; 0.11) | 0.04 (-0.08; 0.17)  -0.00 (-0.12; 0.11)  -0.05 (-0.12; 0.11)  -0.00 (-0.12; 0.11) | 0.06 (-0.05; 0.17)  -0.02 (-0.12; 0.08)  -0.04 (-0.14; 0.06)  -0.02 (-0.13; 0.08) | 0.01  0.93  0.56  0.86 |
| Retinal microvascular diameters composite score, per SD | 1  2  3A  3B | **-0.04 (0.07; -0.02)**  -0.01 (-0.03; 0.02)  -0.01 (-0.04; -0.02)  -0.00 (-0.03; 0.03) | | 0.11 (-0.01; 0.22**)**  0.03 (-0.09; 0.14)  0.01 (-0.11; 0.13)  0.02 (-0.10; 0.13) | -0.14 (-0.24; 0.03)  -0.11 (-0.21; 0.00)  -0.10 (-0.21; 0.00)  -0.08 (-0.19; 0.02) | -0.06 (-0.15; 0.04)  -0.06 (-0.15; 0.04)  -0.06 (-0.16; 0.03)  -0.04 (-0.14; 0.06) | **0.00**  0.09  0.10  0.23 |
| Flicker light-induced increase in retinal diameter composite score, per SD | 1  2  3A  3B | 0.01 (-0.02; 0.04)  -0.01 (-0.04; 0.02)  -0.01 (-0.04; 0.02)  -0.01 (-0.04; 0.02) | | -0.00 (-0.14; 0.13)  -0.02 (-0.16; 0.11)  -0.02 (-0.16; 0.12)  -0.02 (-0.16; 0.11) | -0.09 (-0.21; 0.04)  -0.09 (-0.21; 0.04)  -0.08 (-0.20; 0.04)  -0.09 (-0.21; 0.04) | -0.04 (-0.14; 0.07)  -0.06 (-0.16; 0.05)  -0.06 (-0.17; 0.05)  -0.06 (-0.16; 0.06) | 0.40  0.37  0.33  0.38 |
| Heat-induced skin hyperemia, per SD | 1  2  3A  3B | 0.02 (-0.02; 0.05)  -0.02 (-0.06; 0.02)  -0.03 (-0.06; 0.01)  -0.02 (-0.06; 0.02) | | -0.09 (-0.24; 0.07)  -0.04 (-0.20; 0.11)  -0.06 (-0.22; 0.09)  -0.07 (-0.22; 0.09) | -0.04 (-0.18;0.10)  0.00 (-0.14; 0.14)  -0.01 (-0.15; 0.13)  -0.01 (-0.15; 0.13) | **-0.14 (-0.27; -0.02)**  -0.08 (-0.20;0.04)  -0.10 (-0.22;0.03)  -0.09 (-0.21; 0.04) | 0.15  0.39  0.37  0.48 |
| Logarithmically transformed UAE, per SD | 1  2  3A  3B | 0.01 (-0.02; 0.30)  -0.02 (-0.05; 0.01)  -0.02 (-0.05; 0.00)  -0.02 (-0.05; 0.01)* | | 0.09 (-0.02; 0.20)  0.07 (-0.04; 0.17)  0.06 (-0.05; 0.16)  0.04 (-0.07; 0.14) | **-0.10 (-0.21; -0.00)**  -0.09 (-0.18; 0.01)  -0.07 (-0.16; 0.03)  -0.08 (-0.18; 0.01) | -0.09 (-0.18; 0.00)  -0.06 (-0.14; 0.03)  -0.06 (-0.14; 0.03)  -0.06 (-0.15; 0.02) | **0.00**  **0.02**  **0.02**  **0.04** |
| Plasma biomarkers of MVD composite score, per SD | 1  2  3A  3B | **-0.05 (-0.07; -0.02)**  **-0.06 (-0.09; -0.04)**  **-0.06 (-0.09; -0.04)**  **-0.05 (-0.08; -0.03)** | | **0.34 (0.23; 0.45)**  **0.27 (0.17; 0.38)**  **0.26 (0.16; 0.36)**  **0.24 (0.14; 0.34)** | -0.08 (-0.18; 0.03)  -0.05 (-0.14; 0.05)  -0.00 (-0.10; 0.08)  0.00 (-0.09; 0.09) | **-0.15 (-0.24; -0.06)**  **-0.12 (-0.20; -0.03)**  **-0.10 (-0.18; -0.02)**  -0.08 (-0.16; 0.00) | **0.00**  **0.00**  **0.00**  **0.00** |

Betas and 95% confidence intervals indicate the strength of the association between total alcohol consumption and measures of MVD, where a negative beta indicates less MVD (i.e. less CSVD features, narrower diameters, higher flicker light-induced increase in retinal microvascular diameters, higher heat-induced skin hyperemia, lower UAE, or lower levels of plasma biomarkers of MVD). Total alcohol consumption was entered in the model as a continuous variable (per unit, i.e. 10 g/day), as dummies (none, moderate or high versus light alcohol consumption) or (for the P-for trend analyses) as a categorical variable (none, light, moderate, and high alcohol consumption). Numerical values per SD are presented in the legend of Table 2.

The numbers of participants with complete data on CSVD features, retinal microvascular diameters, flicker light-induced increase in retinal microvascular diameters, heat-induced skin hyperemia, UAE, and plasma biomarkers of MVD respectively are n=2,075, n=2,721, n=2,090, n=1,517, n=3,107, and n=3,078.

*For urinary albumin excretion, the beta in model 3B for total alcohol consumption, investigated as a continuous variable, corresponds with an odds ratio of 0.55 (95% CI, 0.26; 1.16) for 30mg/24-hour greater urinary albumin excretion.

Model 1: crude; Model 2: age, sex, glucose metabolism status (entered as dummies of type 2 diabetes, prediabetes, or other types of diabetes versus normal glucose metabolism status), educational level [low, middle, high]; model 3A: model 2 + waist circumference, smoking status [current, ever, never], diet score; model 3B: model 3A+ office systolic blood pressure, use of antihypertensive medication [yes/no] total cholesterol / HDL cholesterol ratio, lipid-modifying medication, prior cardiovascular disease. Additionally, and only for heat-induced skin hyperemia, baseline skin blood flow was entered in model 1.

Bold denotes P-value<0.05.

Abbreviations: CI: confidence interval; CSVD, cerebral small vessel disease; CRAE, central retina arteriolar equivalent; CRVE, central retinal venular equivalent; SD: standard deviation; PU, perfusion units; UAE, urinary albumin excretion; sICAM-1, soluble intercellular adhesion molecule-1; sVCAM-1, soluble vascular adhesion molecule-1; sE-selectin, soluble E-selectin; vWF, von Willebrand factor; MVD, microvascular dysfunction.

**Supplemental Table S10 Associations of total alcohol** consumption with individual measures used in composite scores, in the general population

|  |  | Total alcohol consumption | | | | |
| --- | --- | --- | --- | --- | --- | --- |
|  | Model | Continuous | None vs. light | Moderate vs. light | High vs. light | P for trend |
|  |  | β (95% CI) | β (95% CI) | β (95% CI) | β (95% CI) | P-value |
| CSVD features | | | | | | |
| Logarithmically transformed white matter hyperintensity volume, per SD | 1  2  3A  3B | **0.05 (0.02; 0.08)**  0.01 (-0.02; 0.04)  0.00 (-0.03; 0.03)  0.01 (-0.02; 0.04) | 0.05 (-0.08; 0.19)  0.01 (-0.11; 0.13)  0.01 (-0.11; 0.13)  -0.00 (-0.12; 0.12) | 0.06 (-0.07; 0.18)  0.00 (-0.11; 0.11)  0.00 (-0.11; 0.11)  0.01 (-0.11; 0.11) | 0.15 (-0.04; 0.26)  0.03 (-0.06; 0.13)  0.02 (-0.08; 0.11)  0.03 (-0.07; 0.12) | **0.02**  0.59  0.79  0.58 |
| Logarithmically transformed number of cerebral microbleeds, per SD | 1  2  3A  3B | **0.03 (0.00; 0.07)**  0.01 (-0.03; 0.04)  0.01 (-0.03; 0.04)  0.01 (-0.03; 0.04) | -0.10 (-0.24; 0.04)  -0.07 (-0.20; 0.07)  -0.07 (-0.21; 0.07)  -0.08 (-0.21; 0.06) | 0.07 (-0.05; 0.19)  0.05 (-0.07; 0.18)  0.06 (-0.07; 0.18)  0.06 (-0.07; 0.18) | 0.01 (-0.10; 0.11)  -0.01 (-0.12; 0.10)  -0.02 (-0.13; 0.09)  -0.01 (-0.12; 0.10) | 0.17  0.54  0.60  0.44 |
| Logarithmically transformed number of lacunar infarcts, per SD | 1  2  3A  3B | 0.00 (-0.03; 0.04)  -0.02 (-0.05; 0.01)  -0.03 (-0.06; 0.01)  -0.02 (-0.06; 0.01) | 0.02 (-0.11; 0.16)  0.05 (-0.09; 0.18)  0.05 (-0.09; 0.19)  0.04 (-0.10; 0.17) | -0.04 (-0.17; 0.08)  -0.06 (-0.18; 0.07)  -0.06 (-0.18; 0.06)  -0.06 (-0.18; 0.06) | -0.04 (-0.15; 0.07)  -0.06 (-0.17; 0.05)  -0.08 (-0.19; 0.03)  -0.07 (-0.18; 0.04) | 0.28  0.10  0.05  0.11 |
| Retinal microvascular diameters | | | | | | |
| CRAE, per SD | 1  2  3A  3B | **-0.06 (-0.09; -0.03)**  -0.02 (-0.04; 0.01)  -0.02 (-0.04; 0.01)  -0.01 (-0.04; 0.02) | **0.12 (0.00; 0.23)**  0.03 (-0.08; 0.15)  0.02 (-0.10; 0.14)  0.03 (-0.09; 0.14) | -0.10 (-0.21; 0.01)  -0.07 (-0.18; 0.04)  -0.07 (-0.18; 0.03)  -0.05 (-0.15; 0.06) | **-0.10 (-0.19; 0.00)**  -0.07 (-0.16; 0.03)  -0.07 (-0.16; 0.03)  -0.04 (-0.14; 0.05) | **0.00**  0.05  0.08  0.20 |
| CRVE, per SD | 1  2  3A  3B | -0.02 (-0.05; 0.01)  0.01 (-0.02; 0.04)  0.00 (-0.03; 0.03)  0.01 (-0.02; 0.04) | **0.08 (0.03; 0.20)**  0.02 (-0.10; 0.14)  0.00 (-0.12; 0.12)  0.01 (-0.11; 0.12) | **-0.15 (-0.26; -0.04)**  **-0.13 (-0.23; -0.02)**  **-0.12 (-0.23; -0.01)**  -0.11 (-0.21; 0.00) | -0.05 (-0.15; 0.04)  -0.03 (-0.13; 0.06)  -0.05 (-0.14; 0.05)  -0.03 (-0.13; 0.06) | **0.01**  0.24  0.22  0.34 |
| Flicker light-induced increase in retinal diameters | | | | | | |
| Arteriolar diameter, per SD | 1  2  3A  3B | 0.01 (-0.02; 0.04)  -0.00 (-0.04; 0.03)  -0.01 (-0.04; 0.03)  -0.00 (-0.04; 0.03) | 0.03 (-0.10; 0.17)  -0.01 (-0.14; 0.13)  -0.01 (-0.14;0.13)  -0.01 (-0.15; 0.12) | **-0.14 (-0.26;-0.02)**  **-0.14 (-0.26; -0.02)**  **-0.14 (-0.26; -0.01)**  **-0.14 (-0.26; -0.02)** | -0.02 (-0.13; 0.08)  -0.04 (-0.15; 0.07)  -0.05 (-0.15; 0.06)  -0.04 (-0.15; 0.07) | 0.26  0.32  0.33  0.38 |
| Venular diameter, per SD | 1  2  3A  3B | -0.00 (-0.03; 0.03)  -0.01 (-0.04; 0.02)  -0.01 (-0.05; 0.02)  -0.01 (-0.05; 0.02) | -0.04 (-0.17; 0.10)  -0.03 (-0.17; 0.10)  -0.02 (-0.16; 0.11)  -0.03 (-0.16; 0.11) | -0.01 (-0.12; 0.13)  -0.01 (-0.12; 0.12)  -0.00 (-0.12; 0.13)  0.00 (-0.12; 0.12) | -0.04 (-0.14; 0.07)  -0.05 (-0.15; 0.06)  -0.05 (-0.16; 0.06)  -0.05 (-0.16; 0.06) | 0.82  0.66  0.56  0.59 |
| Plasma biomarkers of MVD | | | | | | |
| sICAM-1, per SD | 1  2  3A  3B | **-0.06 (-0.08; -0.03)**  **-0.04 (-0.07; -0.02)**  **-0.05 (-0.07; -0.02)**  **-0.04 (-0.06; -0.01)** | **0.41 (0.30; 0.52)**  **0.30 (0.19; 0.41)**  **0.27 (0.16; 0.37)**  **0.25 (0.15; 0.36)** | -0.02 (-0.12; 0.08)  0.01 (-0.09; 0.11)  0.03 (-0.06; 0.13)  0.04 (-0.05; 0.14) | -0.05 (-0.13; 0.04)  -0.02 (-0.11; 0.06)  -0.05 (-0.11; 0.06)  -0.01 (-0.09; 0.08) | **0.00**  **0.00**  **0.00**  **0.00** |
| sVCAM-1, per SD | 1  2  3A  3B | **-0.06 (-0.09; -0.04)**  **-0.09 (-0.12; -0.07)**  **-0.09 (-0.12; -0.06)**  **-0.09 (-0.11; -0.06)** | **0.17 (0.06; 0.28)**  **0.17 (0.07; 0.28)**  **0.19 (0.08; 0.29)**  **0.18 (0.07; 0.28)** | -0.05 (-0.15; 0.05)  -0.05 (-0.14; 0.05)  -0.03 (-0.13; 0.07)  -0.03 (-0.13; 0.07) | -**0.20 (-0.28; -0.11)**  **-0.19 (-0.28; -0.11)**  **-0.18 (-0.27; -0.09)**  **-0.17 (-0.26; -0.09)** | **0.00**  **0.00**  **0.00**  **0.00** |
| sE-selectin, per SD | 1  2  3A  3B | 0.00 (-0.03; 0.03)  -0.00 (-0.03; 0.03)  0.00 (-0.02; 0.03)  0.02 (-0.01; 0.04) | **0.21 (0.10;0.32)**  **0.14 (0.04; 0.25)**  **0.13 (0.03; 0.23)**  **0.11 (0.01; 0.21)** | -0.09 (-0.19; 0.01)  -0.04 (-0.13;0.06)  0.01 (-0.09; 0.10)  0.01 (-0.08; 0.11) | -0.06 (-0.14; 0.03)  0.02 (-0.07; 0.11)  0.04 (- 0.04; 0.13)  0.07 (-0.01; 0.15) | **0.00**  0.12  0.42  0.88 |
| vWF, per SD | 1  2  3A  3B | -0.01 (-0.03; 0.02)  **-0.04 (-0.06; -0.01)**  **-0.04 (-0.06; -0.01)**  **-0.03 (-0.06; -0.01)** | **0.14 (0.03; 0.25)**  **0.13 (0.03; 0.24)**  **0.12 (0.02; 0.23)**  **0.12 (0.02; 0.23)** | -0.05 (- 0.15; 0.06)  -0.05 (-0.15; 0.05)  -0.03 (-0.13; 0.07)  -0.03 (-0.12; 0.07) | **-0.11 (-0.20; -0.02)**  **-0.13 (-0.21; -0.04)**  **-0.12 (-0.20; -0.03)**  **-0.11 (-0.19; -0.02)** | **0.00**  **0.00**  **0.00**  **0.00** |

Betas and 95% confidence intervals represent the difference in CSVD features, retinal microvascular diameters, or measures of MVD (all per SD) per unit of total alcohol consumption or for none, moderate, or high versus light total alcohol consumption where a negative beta indicates less CSVD features, narrower retinal microvascular diameter, or less MVD (i.e. for flicker light-induced increase in retinal arteriolar or venular diameter and individual plasma biomarkers of MVD). Total alcohol consumption was entered in the models as a continuous variable (per unit, i.e. 10 g/day), as dummies (none, moderate or high versus light alcohol consumption) or (for the P-for trend analyses) as a categorical variable (none, light, moderate, and high alcohol consumption). Numerical values per SD are presented in the legend of Table 2. The numbers of participants with complete data on CSVD features, retinal microvascular diameters and plasma biomarkers of MVD respectively are n=2,075; n=2,721; and n=3,078.

Variables per model: Model 1: crude; Model 2: model 1+ age, sex, glucose metabolism status, educational level; Model 3A: model 2 + waist circumference, smoking status, diet score; Model 3B: model 3A+ office systolic blood pressure, use of antihypertensive medication, total cholesterol / HDL cholesterol ratio, lipid-modifying medication, prior cardiovascular disease.

Bold denotes P-value<0.05.

Abbreviations: β, beta; CI: confidence interval; CSVD, cerebral small vessel disease; CRAE, central retinal arteriolar equivalent; CRVE, central retinal venular equivalent; SD: standard deviation; sICAM-1, soluble intercellular adhesion molecule-1; sVCAM-1, soluble vascular adhesion molecule-1; sE-selectin, soluble E-selectin; vWF, von Willebrand factor; MVD, microvascular dysfunction; HDL, high-density lipoprotein;

**Supplemental Table S11 Associations of wine, beer and spirits consumption with MVD measures in the general population and stratified by history of cardiovascular disease status**

|  |  | Wine consumption | | | | | Beer consumption | | | | | Spirits consumption | | | | |
| --- | --- | --- | --- | --- | --- | --- | --- | --- | --- | --- | --- | --- | --- | --- | --- | --- |
|  | Model | Continuous | None vs. light | Moderate vs. light | High vs. light | P for trend | Continuous | None vs. light | Moderate vs. light | High vs. light | P for trend | Continuous | None vs. light | Moderate vs. light | High vs. light | P for trend |
|  |  | β (95% CI) | β (95% CI) | β (95% CI) | β (95% CI) | P- value | β (95% CI) | β (95% CI) | β (95% CI) | β (95% CI) | P- value | β (95% CI) | β (95% CI) | β (95% CI) | β (95% CI) | P- value |
| CSVD features composite score, per SD | | | | | | | | | | | | | | | | |
| General population, n=2,075 | 3B | -0.01 (-0.06; 0.03) | 0.08 (-0.02; 0.19) | -0.02 (-0.15; 0.11) | 0.07 (-0.06; 0.19) | 0.49 | 0.02 (-0.03; 0.07) | -0.01 (-0.11; 0.10) | -0.06 (-0.20; 0.09) | 0.07 (-0.10; 0.24) | 0.76 | -0.08 (-0.24; 0.08) | 0.01 (-0.07; 0.15) | -0.08 (-0.25 0.08) | -0.02 (-0.32; 0.28) | 0.80 |
| With CVD, n=253 | 3B | -0.07 (-0.20; 0.07) | -0.07 (-0.38; 0.23) | -0.21 (-0.63; 0.20) | -0.15 (-0.55; 0.25) | 0.71 | -0.12 (-0.34; 0.10) | 0.18 (-0.14; 0.50) | -0.15 (-0.57; 0.27) | -0.03 (-0.72; 0.66) | 0.17 | -0.45 (-0.95; 0.06) | 0.20 (-0.18; 0.58) | -0.12 (-0.75; 0.50) | -0.06 (-1.45; 1.33) | **0.04** |
| Without CVD, n=1822 | 3B | -0.00 (-0.05; 0.04) | 0.10 (-0.02; 0.21) | 0.01 (-0.13; 0.14) | 0.11 (-0.03; 0.24) | 0.73 | 0.03 (-0.01; 0.08) | -0.04 (-0.15; 0.07) | -0.05 (-0.21; 0.11) | 0.08 (-0.10; 0.26) | 0.33 | 0.00 (-0.17; 0.18) | -0.03 (-0.14; 0.08) | -0.09 (-0.26; 0.09) | -0.03 (-0.34; 0.28) | 0.38 |
| Retinal microvascular diameters composite score, per SD | | | | | | | | | | | | | | | | |
| General population, n=2,721 | 3B | -0.03 (-0.07; 0.01) | 0.01 (-0.09; 0.11) | **-0.13 (-0.25; -0.01)** | -0.08 (-0.20; 0.04) | **0.03** | 0.02 (-0.02; 0.07) | 0.00 (-0.10; 0.10) | 0.03 (-0.11; 0.17) | 0.02 (-0.15; 0.18) | 0.61 | 0.07 (-0.08; 0.21) | 0.02 (-0.08; 0.12) | -0.02 (-0.18; 0.13) | -0.16 (-0.43; 0.11) | 0.78 |
| With CVD, n=459 | 3B | **-0.13 (-0.23; -0.02)** | 0.01 (-0.24; 0.25) | **-0.44 (-0.76; -0.12)** | -0.28 (-0.59; 0.03) | **0.01** | -0.05 (-0.15; 0.04) | -0.07 (-0.30; 0.16) | -0.20 (-0.54; 0.15) | -0.23 (-0.66; 0.20) | 0.70 | **-0.37 (-0.02; -0.73)** | -0.07 (-0.30; 0.16) | -0.20 (-0.54; 0.15) | -0.23 (-0.66; 0.20) | 0.15 |
| Without CVD, n= 2,262 | 3B | -0.02 (-0.06; 0.03) | 0.02 (-0.09; 0.12) | -0.08 (-0.20; 0.05) | -0.04 (-0.17; 0.09) | 0.23 | 0.04 (-0.01; 0.08) | 0.03 (-0.08; 0.13) | 0.07 (-0.08; 0.23) | 0.07 (-0.11; 0.24) | 0.48 | 0.01 (-0.15; 0.17) | 0.05 (-0.06; 0.16) | 0.00 (0.17; 0.17) | -0.17 (-0.47; 0.13) | 0.77 |
| Flicker light-induced increase in retinal diameters composite score, per SD | | | | | | | | | | | | | | | | |
| General population, n=2,090 | 3B | -0.02 (-0.06; 0.03) | 0.02 (-0.10; 0.13) | -0.05 (-0.19; 0.08) | 0.04 (-0.10; 0.17) | 0.99 | 0.00 (-0.05; 0.05) | -0.02 (-0.13; 0.10) | -0.11 (-0.28; 0.05) | 0.07 (-0.12; 0.27) | 0.88 | -0.02 (-0.19; 0.14) | -0.02 (-0.14; 0.09) | -0.09 (-0.27; 0.09) | 0.15 (-0.16; 0.46) | 0.29 |
| With CVD, n=330 | 3B | -0.12 (-0.24; 0.00) | 0.15 (-0.14; 0.45) | -0.21 (-0.59; 0.18) | -0.17 (-0.55; 0.20) | **0.04** | -0.13 (-0.25; -0.01) | 0.10 (-0.18; 0.38) | -0.13 (-0.56; 0.30) | -0.30 (-0.83; 0.23) | 0.21 | 0.19 (-0.23; 0.60) | 0.06 (-0.23; 0.34) | -0.04 (-0.50; 0.42) | 0.07 (-0.69; 0.82) | 0.48 |
| Without CVD, n=1,760 | 3B | 0.00 (-0.05; 0.05) | -0.02 (-0.14; 0.10) | -0.04 (-0.19; 0.10) | 0.05 (-0.09; 0.20) | 0.46 | 0.03 (-0.03; 0.08) | -0.04 (-0.16; 0.09) | -0.12 (-0.30; 0.06) | 0.14 (-0.07; 0.35) | 0.42 | -0.06 (-0.24; 0.12) | -0.04 (-0.16; 0.09) | -0.12 (-0.31; 0.08) | 0.15 (-0.19; 0.50) | 0.39 |
| Heat-induced skin hyperemia, per SD | | | | | | | | | | | | | | | | |
| General population, n=1,517 | 3B | -0.03 (-0.08; 0.03) | -0.10 (-0.24; 0.03) | -0.05 (-0.21; 0.11) | -0.12 (-0.28; 0.04) | 0.98 | -0.03 (-0.08; 0.03) | -0.04 (-0.16; 0.09) | -0.07 (-0.25; 0.11) | -0.17 (-0.38; 0.04) | 0.29 | 0.03 (-0.15; 0.20) | -0.03 (-0.16; 0.10) | -0.09 (-0.28; 0.11) | -0.21 (-0.56; 0.14) | 0.88 |
| With CVD, n=265 | 3B | 0.11 (-0.02; 0.24) | -0.18 (-0.51; 0.14) | 0.10 (-0.33;0.53) | -0.01 (-0.41; 0.40) | 0.20 | -0.04 (-0.15;0.07) | -0.10 (-0.40 ;0.20) | -0.06 (-0.48; 0.35) | -0.43 (-0.99; 0.13) | 0.49 | -0.03 (-0.45; 0.39) | -0.09 (-0.39; 0.21) | -0.17 (-0.61; 0.27) | -0.57 (-1.43; 0.28) | 0.66 |
| Without CVD, n=1252 | 3B | -0.05 (-0.10; 0.01) | -0.10 (-0.25; 0.04) | -0.08 (-0.26; 0.09) | -0.15 (-0.32; 0.03) | 0.64 | -0.02 (-0.08; 0.04) | -0.03 (-0.18; 0.11) | -0.07 (-0.27; 0.13) | -0.12 (-0.35; 0.11) | 0.48 | 0.04 (-0.16; 0.23) | -0.03 (-0.18; 0.12) | -0.08 (-0.31; 0.15) | -0.16 (-0.56; 0.25) | 0.71 |
| Logarithmically transformed UAE, per SD | | | | | | | | | | | | | | | | |
| General population, n=3,107 | 3B | -0.03 (-0.06; 0.01) | **0.11 (0.02; 0.20)** | -0.01 (-0.12; 0.10) | 0.04 (-0.07; 0.14) | 0.07 | -0.01 (-0.04; 0.03) | -0.01 (-0.10; 0.08) | -0.09 (-0.21; 0.04) | -0.04 (-0.18; 0.10) | 0.42 | -0.07 (-0.20; 0.06) | -0.00 (-0.09; 0.09) | -0.11 (-0.24; 0.03) | -0.10 (-0.34; 0.13) | 0.61 |
| With CVD, n=522 | 3B | -0.07 (-0.16; 0.03) | 0.16 (-0.05; 0.38) | 0.10 (-0.18; 0.37) | -0.05 (-0.33; 0.23) | 0.18 | **-0.11 (-0.19; -0.02)** | 0.02 (-0.18; 0.22) | -0.22 (-0.51; 0.07) | **0.40 (-0.78; -0.03)** | **0.03** | -0.16 (-0.45; 0.14) | 0.00 (-0.20; 0.21) | -0.17 (-0.49; 0.14) | -0.26 (-0.87; 0.34) | 0.30 |
| Without CVD, n=2,585 | 3B | -0.02 (-0.06; 0.02) | 0.07 (-0.03; 0.17) | -0.05 (-0.17; 0.07) | 0.03 (-0.09; 0.14) | 0.18 | -0.03 (-0.02; 0.07) | -0.02 (-0.12; 0.08) | -0.06 (-0.20; 0.07) | 0.05 (-0.11; 0.20) | 0.73 | -0.03 (-0.18; 0.12) | -0.00 (-0.10; 0.10) | -0.12 (-0.27; 0.04) | -0.12 (-0.38; 0.15) | 0.91 |
| Plasma biomarkers of MVD composite score, per SD | | | | | | | | | | | | | | | | |
| General population, n=3,078 | 3B | **-0.08 (-0.12; -0.05)** | 0.08 (0.00; 0.17) | **-0.14 (-0.24; -0.04)** | **-0.11 (-0.21; -0.01)** | **0.00** | -0.03 (-0.06; 0.01) | -0.01 (-0.09; 0.08) | -0.06 (-0.18; 0.06) | -0.11 (-0.25; 0.02) | 0.16 | 0.06 (-0.07; 0.18) | 0.01 (-0.09; 0.08) | -0.06 (-0.19; 0.07) | 0.11 (-0.34; 0.12) | 0.46 |
| With CVD, n=519 | 3B | **-0.11 (-0.20; -0.02)** | 0.12 (-0.09; 0.34) | -0.19 (-0.47; 0.09) | -0.09 (-0.37; 0.19) | **0.03** | -0.04 (-0.13; 0.05) | 0.02 (-0.18; 0.23) | -0.00 (-0.30; 0.29) | -0.14 (-0.52; 0.24) | 0.63 | -0.01 (-0.31; 0.28) | 0.01 (-0.20; 0.22) | 0.03 (-0.29; 0.35) | -0.03 (-0.64; 0.59) | 0.95 |
| Without CVD, n=2,559 | 3B | **-0.08 (-0.12; -0.04)** | 0.06 (-0.03; 0.15) | **-0.14 (-0.26; -0.03)** | **-0.12 (-0.23; -0.01)** | **0.00** | -0.02 (-0.06; 0.02) | -0.02 (-0.11; 0.07) | -0.07 (-0.20; 0.06) | -0.11 (-0.26; 0.04) | 0.26 | 0.09 (-0.06; -0.23) | -0.02 (-0.12; 0.08) | -0.08 (-0.23; 0.07) | -0.13 (-0.38; 0.13) | 0.30 |

Betas and 95% confidence intervals represent the difference in CSVD features composite score, retinal microvascular diameters composite score, or (composite score of) measure(s) of MVD (all per SD) per unit of wine, beer, or spirits consumption or for none, moderate, or high versus light total wine, total beer, or total spirits consumption where a negative beta indicates less CSVD features, narrower retinal microvascular diameters, or less MVD (i.e. for the flicker light-induced increase in retinal microvascular diameters composite score, heat-induced skin hyperemia, UAE, or the plasma biomarkers of MVD composite score). Wine, beer, and spirits consumption were entered in the models as a continuous variable (per unit, i.e. 10 g/day), as dummies (none, moderate or high versus light alcohol consumption) or (for the P-for trend analyses) as a categorical variable (none, light, moderate, and high alcohol consumption). For all analyses the value of one SD was numerically comparable to the value of one SD that was presented in the legend of Table 2 (for the general population) or Supplemental Table S4 (stratified by history of cardiovascular disease).

Variables in model 3B: age, sex, glucose metabolism status (where applicable), educational level, waist circumference, smoking status, diet score, office systolic blood pressure, use of antihypertensive medication, total cholesterol / HDL cholesterol ratio, lipid-modifying medication, prior cardiovascular disease (where applicable) and, only for heat-induced skin hyperemia, for baseline skin blood flow.

Bold denotes P-value<0.05.

Abbreviations: β, beta; CI: confidence interval; CSVD, cerebral small vessel disease; CVD, cardiovascular disease; SD: standard deviation; UAE, urinary albumin excretion; MVD, microvascular dysfunction; NGM, normal glucose metabolism; HDL, high-density lipoprotein.

**Supplemental Table S12 Associations of total alcohol consumption with MVD measures, stratified by history of cardiovascular disease status**

|  |  | |  |  |  |  |  |  |  |  |  |  |
| --- | --- | --- | --- | --- | --- | --- | --- | --- | --- | --- | --- | --- |
|  |  | | History of cardiovascular disease | | | | | No history of cardiovascular disease | | | | |
|  | Model | | Continuous | None vs. light | Moderate vs. light | High vs. light | P for trend | Continuous | None vs. light | Moderate vs. light | High vs. light | P for trend |
|  |  | | β (95% CI) | β (95% CI) | β (95% CI) | β (95% CI) | P-value | β (95% CI) | β (95% CI) | β (95% CI) | β (95% CI) | P-value |
| CSVD features composite score,  per SD | 1  2  3A  3B | -0.05 (-0.15; 0.06)  -0.10 (-0.21; 0.01)  **-0.11 (-0.22; 0.00)**  **-0.12 (-0.23; -0.01)** | | 0.22 (-0.13; 0.56)  0.13 (-0.22; 0.48)  0.10 (-0.25; 0.45)  0.02 (-0.34; 0.37) | 0.08 (-0.27; 0.43)  -0.04 (-0.38; 0.31)  -0.06 (-0.40; 0.28)  -0.12 (-0.46; 0.22) | -0.09 (-0.42; 0.24)  -0.19 (-0.52; 0.14)  -0.22 (-0.54; 0.11)  -0.27 (-0.60; 0.06) | 0.17  0.08  0.08  0.08 | **0.07 (0.03; 0.10)**  0.02 (-0.02; 0.05)  0.01 (-0.02; 0.05)  0.01 (-0.02; 0.05) | -0.12 (-0.27; 0.03)  -0.08 (-0.22; 0.06)  -0.08 (-0.21; 0.07)  -0.07 (-0.21; 0.07) | 0.05 (-0.09; 0.18)  0.00 (-0.12; 0.13)  0.01 (-0.12; 0.13)  0.01 (-0.12; 0.13) | 0.11 (-0.00; 0.22)  0.02 (-0.09; 0.12)  0.00 (-0.11; 0.11)  0.01 (-0.10; 0.11) | **0.00**  0.27  0.41  0.40 |
| Retinal microvascular diameters composite score, per SD | 1  2  3A  3B | **-0.09 (0.16; 0.03)**  -0.06 (-0.13; 0.01)  -0.05 (-0.12; 0.02)  -0.05 (-0.12; 0.02) | | -0.13 (-0.38; 0.13)  -0.17 (-0.44; 0.09)  -0.19 (-0.46; 0.07)  -0.17 (-0.44; 0.09) | **-0.38 (-0.64; -0.13)**  **-0.29 (-0.55; -0.04)**  **-0.29 (-0.55; -0.04)**  **-0.27 (-0.53; 0.01)** | **-0.38 (-0.61; -0.14)**  **-0.35 (-0.59; -0.11)**  **-0.34 (-0.58; -0.10)**  **-0.33 (-0.57; 0.08)** | **0.01**  **0.03**  0.06  0.06 | **-0.03 (-0.06; -0.00)**  0.01 (-0.02; 0.04)  0.01 (-0.03; 0.04)  0.01 (-0.02; 0.04) | **0.16 (0.03; 0.29)**  0.08 (-0.05; 0.21)  0.06 (-0.07; 0.20)  0.06 (-0.07; 0.19) | -0.08 (-0.20; 0.04)  -0.06 (-0.18; 0.06)  -0.06 (-0.18; 0.06)  -0.04 (-0.16; 0.08) | -0.02 (-0.12; 0.08)  0.01 (-0.10; 0.11)  0.00 (-0.10; 0.10)  0.01 (-0.09; 0.12) | **0.02**  0.41  0.41  0.62 |
| Flicker light-induced increase in retinal diameters composite score, per SD | 1  2  3A  3B | -0.06 (-0.14; 0.02)  **-0.09 (-0.17; -0.01)**  **-0.09 (-0.18; -0.01)**  **-0.10 (-0.19; -0.02)** | | 0.15 (-0.15; 0.46)  0.10 (-0.22; 0.42)  0.10 (-0.22; 0.41)  0.09 (-0.23; 0.41) | -0.09 (-0.40; 0.22)  -0.18 (-0.49; 0.14)  -0.17 (-0.49; 0.15)  -0.18 (-0.50; 0.14) | -0.20 (0.48; 0.08)  -0.24 (0.52; 0.04)  -0.26 (-0.54; 0.03)  -0.29 (0.58; 0.00) | **0.02**  **0.02**  **0.02**  **0.01** | 0.03 (-0.01; 0.06)  0.01 (-0.03; 0.05)  0.01 (-0.03; 0.04)  0.01 (-0.03; 0.05) | -0.05 (-0.20; 0.10)  -0.06 (-0.21; 0.09)  -0.05 (-0.21; 0.10)  -0.06 (-0.21; 0.10) | -0.08 (-0.21; 0.06)  -0.07 (-0.20; 0.06)  -0.06 (-0.20; 0.07)  -0.07 (-0.20; 0.07) | 0.00 (-0.12; 0.12)  -0.02 (-0.14; 0.10)  -0.02 (-0.14; 0.10)  -0.02 (-0.14; 0.10) | 0.73  0.95  0.99  0.93 |
| Heat-induced skin hyperemia | 1  2  3A  3B | 0.02 (-0.06; 0.09)  0.02 (-0.06; 0.10)  0.02 (-0.06; 0.10)  0.02 (-0.06; 0.10) | | -0.25 (-0.59; 0.09)  **-0.37 (0.70; -0.04)**  **-0.36 (-0.67; -0.03)**  **-0.36 (-0.69; -0.03)** | -0.03 (-0.38; 0.32)  **-0.07 (-0.40; -0.27)**  -0.06 (-0.40; 0.28)  -0.03 (-0.37; 0.32**)** | -0.14 (-0.46; -0.18)  0.01 (-0.30; 0.31)  -0.02 (-0.33; 0.29)  -0.00 (-0.32; 0.31) | 0.71  0.10  0.15  0.12 | 0.02 (-0.02; 0.06)  -0.03 (-0.07; 0.02)  -0.03 (-0.07; 0.01)  -0.03 (-0.07; 0.01) | -0.06 (-0.24; 0.12)  0.02 (-0.15; 0.20)  -0.01 (-0.19; 0.17)  -0.01 (-0.19; 0.17) | -0.04 (-0.20; 0.12)  0.01 (-0.15; 0.16)  -0.01 (-0.17; 0.14)  -0.01 (-0.16; 0.15) | -0.14 (-0.27; 0.00)  -0.09 (-0.22; 0.05)  -0.10 (-0.24; 0.04)  -0.10 (-0.24; 0.04) | 0.13  0.16  0.17  0.19 |
| Logarithmically transformed UAE, per SD | 1  2  3A  3B* | **-0.08 (-0.14; -0.02)**  **-0.11 (-0.17; -0.05)**  **-0.10 (-0.16; -0.04)**  **-0.10 (-0.16; -0.04)** | | **0.37 (0.14; 0.60)**  **0.25 (0.03; 0.48)**  **0.23 (0.01; 0.45)**  0.18 (-0.05; 0.40) | -0.11 (-0.35; 0.13)  **-0.23 (-0.46; -0.01)**  -0.21 (-0.44; 0.02)  **-0.24 (-0.46; -0.01)** | **-0.23 (-0.45; -0.01)**  -0.21 (-0.41; 0.01)  -0.19 (-0.40; 0.02)  -0.20 (-0.41; 0.01) | **0.00**  **0.00**  **0.00**  **0.00** | **0.03 (0.00; 0.06)**  0.01 (-0.02; 0.03)  0.00 (-0.03; 0.03)  0.00 (-0.03; 0.03) | -0.03 (-0.15; 0.10)  -0.03 (-0.15; 0.10)  -0.03 (-0.15; 0.09)  -0.04 (-0.15; 0.09) | -0.10 (-0.21; 0.01)  -0.07 (-0.17; 0.04)  -0.05 (-0.16; 0.05)  -0.06 (-0.17; 0.04) | -0.05 (-0.14; 0.05)  -0.03 (-0.13; 0.06)  -0.04 (-0.13; 0.06)  -0.04 (-0.13; 0.06) | 0.39  0.58  0.61  0.62 |
| Plasma biomarkers of MVD composite score, per SD | 1  2  3A  3B | **-0.06 (-0.12; 0.00)**  **-0.08 (-0.14; -0.02)**  **-0.08 (-0.14; -0.02)**  **-0.07 (-0.13; -0.01)** | | **0.54 (0.31; 0.77)**  **0.38 (0.16; 0.60)**  **0.36 (0.14; 0.59)**  **0.38 (0.15; 0.60)** | 0.16 (-0.08; 0.40)  0.06 (-0.17; 0.29)  0.08 (-0.14; 0.31)  0.12 (-0.11; 0.35) | -0.09 (-0.31; 0.13)  -0.09 (-0.30; 0.12)  -0.10 (-0.31; 0.11)  -0.06 (-0.27; 0.16) | **0.00**  **0.00**  **0.00**  **0.01** | **-0.04 (-0.06; -0.01)**  **-0.05 (-0.08; -0.03)**  **-0.05 (-0.08; -0.02)**  **-0.05 (-0.07; -0.02)** | **0.24 (0.12; 0.36)**  **0.20 (0.09; 0.32)**  **0.19 (0.08; 0.30)**  **0.18 (0.07; 0.30)** | **-0.13 (-0.24; -0.02)**  -0.08 (-0.19; 0.02)  -0.04 (-0.15; 0.06)  -0.04 (-0.14; 0.07) | **-0.15 (-0.24; -0.05)**  **-0.12 (-0.21; -0.03)**  **-0.10 (-0.19; -0.01)**  -0.09 (-0.18; 0.00) | **0.00**  **0.00**  **0.00**  **0.00** |

Betas and 95% confidence intervals represent the difference in CSVD features composite score, retinal microvascular diameters composite score, or (composite score of) measure(s) of MVD (all per SD) per unit of total alcohol consumption or for none, moderate, or high versus light total alcohol consumption stratified by history of cardiovascular disease status where a negative beta indicates less CSVD features, narrower retinal microvascular diameters, or less MVD (i.e. for the flicker light-induced increase in retinal microvascular diameters composite score, heat-induced skin hyperemia, UAE, or the plasma biomarkers of MVD composite score). Total alcohol consumption was entered in the models as a continuous variable (per unit, i.e. 10 g/day), as dummies (none, moderate or high versus light alcohol consumption) or (for the P-for trend analyses) as a categorical variable (none, light, moderate, and high alcohol consumption). Numerical values per SD are presented in the legend of Supplemental Table S6.

The number of participants with and without a history of cardiovascular disease respectively are n=253 and n=1,822 for CSVD features, n=459 and n=2,262 for retinal microvascular diameters, n=330 and n=1,760 for flicker light-induced increase in retinal microvascular diameters, n=265 and n=1,252 for heat-induced skin hyperemia, n=522 and n=2,585 for UAE, and n=519 and n=2,559 for plasma biomarkers of MVD.

Variables per model: Model 1: crude; Model 2: model 1+ age, sex, glucose metabolism status, educational level; Model 3A: model 2 + waist circumference, smoking status, diet score; Model 3B: model 3A + office systolic blood pressure, use of antihypertensive medication, total cholesterol / HDL cholesterol ratio, lipid-modifying medication. Additionally, and only for heat-induced skin hyperemia, baseline skin blood flow was entered in model 1.

Bold denotes P-value<0.05.

* For respectively individuals with and without a history of cardiovascular disease the betas of total alcohol consumption (per unit) in model 3B correspond with the following odds ratios (95% CI) for 30mg/24-hour greater UAE: 0.89 (0.83; 0.96) and 1.00 (0.98; 1.03).

Abbreviations: β, beta; CI, confidence interval; SD: standard deviation; CSVD, cerebral small vessel disease; UAE, urinary albumin excretion; MVD, microvascular dysfunction. HDL, high-density lipoprotein.

**Supplemental Table S13 Associations of total alcohol consumption with MVD measures**, stratified by glucose metabolism status

|  |  |  |  |  |  |  |
| --- | --- | --- | --- | --- | --- | --- |
|  |  | Total alcohol consumption | | | | |
|  | Model | Continuous | None vs. light | Moderate vs. light | High vs. light | P for trend |
|  |  | β (95% CI) | β (95% CI) | β (95% CI) | β (95% CI) | P-value |
| Type 2 diabetes |  |  |  |  |  |  |
| CSVD features composite score,  per SD | 1  2  3A  3B | 0.04 (-0.03; 0.11)  -0.00 (-0.07; 0.06)  -0.01 (-0.07; 0.06)  0.00 (-0.06; 0.07) | **-0.03 (-0.27; -0.22)**  0.06 (-0.19; 0.30)  0.04 (-0.20; 0.29)  -0.01 (-0.26; 0.23) | -0.04 (-0.31; 0.23**)**  -0.07 (-0.33; 0.19)  -0.08 (-0.34; 0.18)  -0.10 (-0.35; 0.16) | 0.12 (-0.12; 0.36)  0.04 (-0.19; 0.27)  0.02 (-0.21; 0.25)  0.02 (-0.21; 0.26) | 0.31  0.84  0.79  0.93 |
| Retinal microvascular diameters composite score, per SD | 1  2  3A  3B | **-0.05 (0.10; -0.01)**  -0.03 (-0.08; 0.02)  -0.03 (-0.08; 0.02)  -0.01 (-0.06; 0.04) | 0.10 (-0.09; 0.28)  0.01 (-0.19; 0.20)  0.00 (-0.19; 0.20)  -0.02 (-0.21; 0.18) | -0.06 (-0.28; 0.16)  -0.07 (-0.28; 0.15)  -0.05 (-0.27; 0.16)  -0.04 (-0.26; 0.18) | -0.13 (-0.32; 0.06)  -0.10 (-0.29; 0.09)  -0.10 (-0.29; 0.09)  -0.06 (-0.25; 0.13) | **0.02**  0.23  0.28  0.63 |
| Flicker light-induced increase in retinal diameters composite score, per SD | 1  2  3A  3B | -0.01 (-0.06; 0.03)  -0.02 (-0.07; 0.02)  -0.02 (-0.07; 0.02)  -0.03 (-0.07; 0.02) | -0.05 (-0.22; 0.12)  -0.01 (-0.19; 0.16)  -0.01 (-0.18; 0.17)  0.02 (-0.16; 0.19) | -0.05 (-0.25; 0.15)  -0.04 (-0.24; 0.16)  -0.04 (-0.24; 0.17)  -0.04 (-0.25; 0.16) | -0.17 (-0.35; 0.01)  -0.18 (-0.35; 0.00)  -0.17 (-0.35; 0.01)  -0.17 (-0.35; 0.01) | 0.15  0.07  0.07  0.05 |
| Heat-induced skin hyperemia,  per SD | 1  2  3A  3B | -0.01 (-0.07; 0.05)  -0.06 (-0.11; 0.00)  **-0.06 (-0.12; -0.00)**  -0.05 (-0.11; 0.01) | **-0.29 (-0.53; -0.05)**  -0.19 (-0.34; 0.15)  -0.16 (-0.40; 0.08)  -0.19 (-0.44; 0.05) | **-0.38 (-0.67; -0.09)**  **-0.34 (-0.63; -0.05)**  **-0.33 (-0.62; -0.05)**  **-0.33 (-0.62; -0.04)** | **-0.36 (-0.60; -0.11)**  **-0.33 (-0.57; -0.10)**  **-0.35 (-0.59; -0.11)**  **-0.32 (-0.56; -0.08)** | 0.18  **0.01**  **0.03**  0.08 |
| Logarithmically transformed UAE,  per SD | 1  2  3A  3B* | -0.03 (-0.08; 0.01)  **-0.07 (-0.12; -0.03)**  **-0.07 (-0.12; -0.03)**  **-0.07 (-0.12; -0.03)** | -0.11 (-0.28; 0.07)  0.03 (-0.15; 0.21)  0.00 (-0.17; 0.18)  -0.02 (-0.20; 0.15) | **-0.24 (-0.44; -0.04)**  **-0.24 (-0.44; -0.04)**  **-0.22 (-0.42; -0.02**)  **-0.25 (-0.45; -0.06)** | **-0.21 (-0.39; -0.03)**  **-0.20 (0.37; -0.02)**  **-0.21 (0.38; -0.03)**  **-0.21 (-0.38; -0.04)** | 0.08  **0.00**  **0.01**  **0.01** |
| Plasma biomarkers of MVD composite score, per SD | 1  2  3A  3B | -0.04 (-0.08; 0.01)  -0.04 (-0.09; 0.01)  -0.03 (-0.08; 0.01)  -0.02 (-0.06; 0.03) | **0.32 (0.15; 0.49)**  **0.37 (0.19; 0.55)**  **0.37 (0.20; 0.54)**  **0.31 (0.14; 0.49)** | 0.08 (-0.13; 0.28)  0.08 (-0.12; 0.29)  0.14 (-0.06; 0.33)  0.14 (-0.05; 0.34) | -0.03 (-0.20; 0.15)  -0.01 (-0.19; 0.16)  0.02 (-0.15; 0.20)  0.06 (-0.12; 0.23) | **0.00**  **0.00**  **0.01**  0.05 |
| Prediabetes |  |  |  |  |  |  |
| CSVD features composite score,  per SD | 1  2  3A  3B | -0.00 (-0.07; 0.06)  -0.02 (-0.09; 0.04)  -0.02 (-0.09; 0.04)  -0.02 (-0.09; 0.04) | -0.06 (-0.42; 0.29)  0.03 (-0.31; 0.37)  0.03 (-0.32; 0.37)  0.00 (-0.34; 0.34) | 0.08 (-0.25; 0.40)  0.01 (-0.30; 0.31)  -0.06 (-0.37; 0.25)  -0.11 (-0.41; 0.20) | -0.02 (-0.29; 0.25)  -0.10 (-0.35; 0.15)  -0.14 (-0.39; 0.11)  -0.16 (-0.41; 0.09) | 0.89  0.34  0.22  0.20 |
| Retinal microvascular diameters composite score, per SD | 1  2  3A  3B | -0.00 (-0.06; 0.06)  0.04 (-0.03; 0.10)  0.02 (-0.04; 0.08)  0.03 (-0.03; 0.09) | 0.17 (-0.16; 0.50)  0.12 (-0.22; 0.45)  0.17 (-0.16; 0.49)  0.17 (-0.15; 0.50) | -0.20 (-0.48; 0.09)  -0.15 (-0.44; 0.14)  -0.05 (-0.34; 0.23)  -0.04 (-0.32; 0.25) | -0.04 (-0.28; 0.21)  0.00 (-0.24; 0.24)  0.01 (-0.23; 0.24)  0.02 (-0.22; 0.26) | 0.26  0.61  0.51  0.59 |
| Flicker light-induced increase in retinal diameters composite score, per SD | 1  2  3A  3B | -0.02 (-0.07; 0.03)  -0.03 (-0.08; 0.02)  -0.04 (-0.09; 0.02)  -0.05 (-0.10; 0.01) | -0.02 (-0.33; 0.28)  0.03 (-0.28; 0.34)  -0.00 (-0.32; 0.31)  0.02 (-0.30; 0.34) | -0.00 (-0.27; 0.27)  -0.06 (-0.33; 0.22)  -0.03 (-0.31; 0.24)  -0.04 (-0.32; 0.24) | -0.07 (-0.29; 0.15)  -0.14 (-0.36; 0.08)  -0.13 (-0.36; 0.09)  -0.16 (-0.39; 0.07) | 0.61  0.17  0.24  0.14 |
| Heat-induced skin hyperemia,  per SD | 1  2  3A  3B | -0.07 (-0.15; 0.02)  **-0.13 (-0.22; -0.04)**  **-0.13 (-0.23; -0.04)**  **-0.13 (-0.23; -0.03)** | 0.06 (-0.41; 0.53)  0.13 (-0.34; 0.60)  0.13 (-0.33; 0.60)  0.13 (-0.35; 0.60) | 0.00 (-0.36; 0.36)  -0.10 (-0.47; 0.26)  -0.07 (-0.43; 0.30)  -0.05 (-0.42; 0.32) | -0.26 (-0.59; 0.06)  -0.33 (-0.66; 0.00)  -0.31 (-0.64; 0.03)  -0.29 (-0.63; 0.06) | 0.07  **0.02**  **0.03**  0.05 |
| Logarithmically transformed UAE,  per SD | 1  2  3A  3B* | 0.05 (-0.01; 0.11)  0.01 (-0.04; 0.07)  0.02 (-0.04; 0.08)  0.01 (-0.04; 0.07) | -0.09 (-0.40; 0.21)  0.03 (-0.28; 0.34)  0.01 (-0.30; 0.32)  -0.04 (-0.35; 0.26) | 0.01 (-0.26; 0.28)  -0.06 (-0.33; 0.20)  -0.07 (-0.34; 0.19)  -0.14 (-0.41; 0.12) | 0.05 (-0.18; 0.27)  -0.02 (-0.24; 0.20)  -0.01 (-0.23; 0.22)  -0.04 (-0.27; 0.18) | 0.40  0.73  0.89  0.80 |
| Plasma biomarkers of MVD composite score, per SD | 1  2  3A  3B | -0.03 (-0.08; 0.03)  -0.05 (-0.11; 0.01)  -0.04 (-0.10; 0.01)  -0.04 (-0.10; 0.02) | **0.41 (0.10; 0.71)**  **0.48 (0.18; 0.79)**  **0.44 (0.14; 0.73)**  **0.42 (0.12; 0.72)** | 0.12 (-0.15; 0.39)  0.07 (-0.19; 0.33)  0.05 (-0.20; 0.31)  0.05 (-0.21; 0.31) | -0.04 (-0.26; 0.18)  -0.07 (-0.29; 0.15)  -0.05 (-0.27; 0.16)  -0.04 (-0.67; 0.18) | **0.03**  **0.01**  **0.02**  **0.03** |
| Normal glucose metabolism | | | | | | |
| CSVD features composite score,  per SD | 1  2  3A  3B | **0.08 (0.04; 0.12)**  0.02 (-0.03; 0.06)  0.00 (-0.04; 0.05)  0.01 (-0.04; 0.05) | -0.06 (-0.25; 0.12)  -0.03 (-0.21; 0.15)  -0.03 (-0.21; 0.15)  -0.04 (-0.22; 0.14) | 0.11 (-0.04; 0.27)  0.02 (-0.13; 0.17)  0.02 (-0.13; 0.17)  0.03 (-0.12; 0.17) | **0.14 (0.00; 0.28)**  0.00 (-0.13; 0.13)  -0.03 (-0.16; 0.11)  -0.01 (-0.14; 0.12) | **0.01**  0.77  0.92  0.84 |
| Retinal microvascular diameters composite score, per SD | 1  2  3A  3B | **-0.07 (-0.11; -0.03)**  -0.02 (-0.06; 0.03)  -0.02 (-0.06; 0.03)  -0.01 (-0.05; 0.03) | 0.11 (-0.06; 0.28)  0.03 (-0.14; 0.20)  0.02 (-0.15; 0.19)  0.05 (-0.12; 0.22) | -0.14 (-0.28; -0.00)  -0.11 (-0.25; 0.03)  -0.11 (-0.25; 0.03)  -0.09 (-0.23; 0.05) | -0.09 (-0.21; 0.04)  -0.07 (-0.19; 0.06)  -0.07 (-0.19; 0.06)  -0.05 (-0.17; 0.08) | **0.02**  0.14  0.19  0.22 |
| Flicker light-induced increase in retinal diameters composite score, per SD | 1  2  3A  3B | **0.05 (0.01; 0.09)**  0.02 (-0.02; 0.07)  0.02 (-0.02; 0.07)  0.03 (-0.02; 0.07) | -0.08 (-0.24; 0.09)  -0.05 (-0.21; 0.11)  -0.05 (-0.21; 0.11)  -0.06 (-0.22; 0.11) | -0.06 (-0.18; 0.07)  -0.07 (-0.20; 0.06)  -0.08 (-0.20; 0.05)  **-**0.07 (-0.20; 0.06) | 0.06 (-0.05; 0.17)  0.03 (-0.08; 0.15)  0.03 (-0.09; 0.14)  0.04 (-0.08; 0.16) | 0.11  0.40  0.46  0.31 |
| Heat-induced skin hyperemia,  per SD | 1  2  3A  3B | **0.08 (0.02; 0.14)**  0.04 (-0.02; 0.10)  0.03 (-0.03; 0.10)  0.04 (-0.02; 0.10) | -0.14(-0.38; 0.10)  -0.04 (-0.27; 0.20)  -0.05 (-0.29; 0.19)  -0.06 (-0.30; 0.18) | 0.15 (-0.04; 0.38)  0.15 (-0.04; 0.34)  0.13 (-0.06; 0.32)  0.13 (-0.06; 0.32) | 0.05 (-0.12; -0.22)  0.09 (-0.08; 0.26)  0.07 (-0.10; 0.25)  0.08 (-0.10; 0.25) | 0.12  0.17  0.22  0.19 |
| Logarithmically transformed UAE,  per SD | 1  2  3A  3B* | **0.05 (0.01; 0.09)**  0.02 (-0.02; 0.06)  0.02 (-0.02; 0.06)  0.02 (-0.02; 0.06) | 0.06 (-0.10; 0.22)  0.10 (-0.07; 0.26)  0.10 (-0.06; 0.26)  0.07 (-0.09; 0.23) | 0.02 (-0.11; 0.15)  -0.01 (-0.14; 0.12)  0.01 (-0.13; 0.14)  -0.00 (-0.13; 0.13) | 0.05 (-0.06; 0.17)  0.02 (-0.09; 0.14)  0.02 (-0.10; 0.14)  0.02 (-0.10; 0.13) | 0.67  0.65  0.62  0.75 |
| Plasma biomarkers of MVD composite score, per SD | 1  2  3A  3B | -0.04 (-0.08; 0.00)  **-0.09 (-0.13; -0.05)**  **-0.10 (-0.14; -0.06)**  **-0.09 (-0.13; -0.05)** | 0.02 (-0.14; 0.18)  0.07 (-0.09; 0.22)  0.06 (-0.10; 0.21)  0.05 (-0.11; 0.20) | **-0.17 (-0.30; -0.04)**  **-0.20 (-0.33; -0.07)**  **-0.17 (-0.30; -0.04)**  **-0.15 (-0.28; -0.03)** | **-0.18 (-0.30; -0.06)**  **-0.22 (-0.34; -0.11)**  **-0.22 (-0.33; -0.10)**  **-0.20 (-0.31; -0.08)** | **0.00**  **0.00**  **0.00**  **0.00** |

Betas and 95% confidence intervals represent the difference in CSVD features composite score, retinal microvascular diameters composite score, or (composite score of) measure(s) of MVD (all per SD) per unit of total alcohol consumption or for none, moderate, or high versus light total alcohol consumption stratified by glucose metabolism status where a negative beta indicates less CSVD features, narrower retinal microvascular diameters, or less MVD (i.e. for the flicker light-induced increase in retinal microvascular diameters composite score, heat-induced skin hyperemia, UAE, or the plasma biomarkers of MVD composite score). Total alcohol consumption was entered in the models as a continuous variable (per unit, i.e. 10 g/day), as dummies (none, moderate or high versus light alcohol consumption) or (for the P-for trend analyses) as a categorical variable (none, light, moderate, and high alcohol consumption). The numerical values per SD are presented in the legend of Supplemental Table S6.

The number of participants with type 2 diabetes, prediabetes, and normal glucose metabolism respectively are n=459, n=321, and n=1,274 for CSVD features, n=758, n=403, and n=1,533 for retinal microvascular diameters, n=563, n=307, and n=1,197 for flicker light-induced increase in retinal microvascular diameters, n= 439, n=230, n=825 for heat-induced skin hyperemia, n= 854, n=460, n=1,760 for UAE, and n= 849, n=455, n=1,742 for plasma biomarkers of MVD.

Variables per model: Model 1: crude; Model 2: model 1+ age, sex, educational level; Model 3A: model 2 + waist circumference, smoking status, diet score; Model 3B: model 3A+ office systolic blood pressure, use of antihypertensive medication, total cholesterol / HDL cholesterol ratio, lipid-modifying medication, prior cardiovascular disease. Additionally, and only for heat-induced skin hyperemia, baseline skin blood flow was entered in model 1.

Bold denotes P-value<0.05.

* For individuals with respectively type 2 diabetes, prediabetes, or normal glucose metabolism the betas of total alcohol consumption (per unit) in model 3B correspond with the following odds ratios (95% CI) for 30mg/24-hour greater UAE: 0.92 (0.88; 0.97), 1.01 (0.96; 1.07), and 1.02 (0.98; 1.05).

Abbreviations: β, beta; CI, confidence interval; SD: standard deviation; CSVD, cerebral small vessel disease; UAE, urinary albumin excretion; MVD, microvascular dysfunction. HDL, high-density lipoprotein.

**Supplemental Table S14 Associations of total alcohol consumption with MVD measures, stratified hypertension status**

|  |  | |  |  |  |  |  |  |  |  |  |  |
| --- | --- | --- | --- | --- | --- | --- | --- | --- | --- | --- | --- | --- |
|  |  | | With hypertension | | | | | Without hypertension | | | | |
|  | Model | | Continuous | None vs. light | Moderate vs. light | High vs. light | P for trend | Continuous | None vs. light | Moderate vs. light | High vs. light | P for trend |
|  |  | | β (95% CI) | β (95% CI) | β (95% CI) | β (95% CI) | P-value | β (95% CI) | β (95% CI) | β (95% CI) | β (95% CI) | P-value |
| CSVD features composite score,  per SD | 1  2  3A  3B | -0.02 (-0.06; 0.03)  -0.03 (-0.07; 0.01)  -0.04 (-0.08; 0.00)  -0.04 (-0.08; 0.01) | | -0.06 (-0.24; 0.12)  -0.04 (-0.22; 0.14)  -0.05 (-0.22; 0.13)  -0.05 (-0.23; 0.13) | -0.04 (-0.21; 0.13)  -0.08 (-0.24; 0.09)  -0.07 (-0.23; 0.09)  -0.08 (-0.24; 0.08) | -0.09 (-0.24; 0.06)  -0.13 (-0.27; 0.02)  **-0.15 (-0.29; -0.00)**  **-0.15 (-0.29; -0.00)** | 0.45  0.13  0.09  0.17 | **0.15 (0.10; 0.20)**  **0.08 (0.03; 0.12)**  **0.06 (0.01; 0.12)**  **0.06 (0.01; 0.12)** | 0.03 (-0.17; 0.23)  0.04 (-0.16; 0.23)  0.05 (-0.14; 0.24)  0.04 (-0.15; 0.24) | **0.21 (0.03; 0.39)**  0.12 (-0.05; 0.28)  0.11 (-0.06; 0.28)  0.11 (-0.06; 0.28) | **0.32 (0.17; 0.48)**  **0.15 (0.00; 0.30)**  0.12 (-0.03; 0.27)  0.12 (-0.03; 0.27) | **0.00**  0.06  0.19  0.19 |
| Retinal microvascular diameters composite score, per SD | 1  2  3A  3B | **-0.04 (-0.00; -0.07)**  -0.01 (-0.04; 0.03)  -0.01 (-0.04; 0.03)  0.00 (-0.03; 0.04) | | **0.24 (0.10; 0.39)**  **0.16 (0.01; 0.31)**  0.14 (0.01; 0.30)  0.14 (0.01; 0.29) | **-0.15 (-0.29; -0.01)**  -0.13 (-0.27; 0.02)  -0.12 (-0.26; 0.04)  -0.10 (-0.24; 0.04) | -0.05 (-0.17; 0.08)  -0.03 (-0.15; 0.10)  -0.03 (-0.16; 0.10)  -0.00 (-0.13; 0.12) | **0.00**  **0.03**  **0.04**  0.11 | **-0.05 (-0.10; -0.01)**  -0.00 (-0.05; 0.05)  -0.01 (-0.06; 0.04)  -0.00 (-0.05; 0.05) | -0.08 (-0.27; 0.10)  -0.15 (-0.34; 0.03)  -0.18 (-0.36; 0.01)  -0.16 (-0.35; 0.03) | -0.11 (-0.27; 0.06)  -0.07 (-0.23; 0.09)  -0.07 (-0.24; 0.09)  -0.05 (-0.22; 0.11) | -0.12 (-0.27; 0.02)  -0.08 (-0.23; 0.06)  -0.09 (-0.24; 0.05)  -0.08 (-0.23; 0.07) | 0.22  0.93  0.96  0.96 |
| Flicker light-induced increase in retinal diameters composite score, per SD | 1  2  3A  3B | -0.02 (-0.06; 0.02)  -0.03 (-0.07; 0.01)  -0.03 (-0.07; 0.01)  -0.03 (-0.07; 0.01) | | -0.01 (-0.19; 0.16)  -0.03 (-0.21; 0.15)  -0.04 (-0.22; 0.14)  -0.05 (-0.23; 0.13) | 0.00 (-0.17; 0.17)  0.03 (-0.14; 0.19)  0.04 (-0.13; 0.20)  0.02 (-0.14; 0.19) | -0.13 (-0.28; 0.01)  -0.12 (-0.27; 0.03)  -0.11 (-0.26; 0.03)  -0.12 (-0.27; 0.03) | 0.09  0.18  0.27  0.25 | 0.05 (-0.00; 0.11)  0.03 (-0.03; 0.09)  0.02 (-0.04; 0.07)  0.02 (-0.04; 0.08) | -0.02 (-0.22; 0.19)  -0.02 (-0.23; 0.19)  -0.02 (-0.23; 0.20)  -0.01 (-0.22; 0.21) | **-0.19 (-0.38; -0.01)**  **-0.22 (-0.41; -0.04)**  **-0.24 (-0.42; -0.05)**  **-0.22 (-0.41; -0.03)** | 0.07 (-0.09; 0.23)  0.01 (-0.16; 0.17)  -0.02 (-0.19; 0.14)  -0.00 (-0.17; 0.17) | 0.48  0.94  0.64  0.78 |
| Heat-induced skin hyperemia, per SD | 1  2  3A  3B | -0.01 (-0.05; 0.04)  **-0.05 (-0.09; -0.00)**  **-0.05 (-0.10; -0.01)**  **-0.05 (-0.10; -0.01)** | | **-0.25 (-0.44; -0.05**)  -0.10 (-0.29; 0.10)  -0.10 (-0.30; 0.09)  -0.13 (-0.32; 0.07) | -0.08 (-0.27; 0.11)  -0.02 (-0.20; 0.17)  -0.02 (-0.20; 0.16)  -0.02 (-0.20; 0.16) | **-0.26 (0.43; -0.09)**  **-0.17 (-0.33; -0.01)**  **-0.18 (-0.34; -0.02)**  **-0.17 (-0.34; -0.01)** | 0.24  0.20  0.18  0.29 | 0.05 (-0.01; 0.11)  0.04 (-0.03; 0.10)  0.03 (-0.04; 0.10)  0.03 (-0.04; 0.10) | 0.09 (-0.17; 0.35)  0.06 (-0.20; 0.32)  0.03 (-0.24; 0.29)  0.02 (-0.25; 0.29) | -0.01 (-0.23; 0.21)  0.02 (-0.20; 0.25)  0.00 (-0.22; 0.22)  -0.01 (-0.23; 0.22) | -0.01 (-0.20; 0.19)  0.03 (-0.16; 0.23)  0.02 (-0.18; 0.22)  0.02 (-0.19; 0.22) | 0.59  0.97  0.97  0.96 |
| Logarithmically transformed UAE, per SD | 1  2  3A  3B* | -0.01 (-0.04; 0.02)  -0.02 (-0.06; 0.01)  -0.02 (-0.06; 0.01)  -0.03 (-0.06; 0.01) | | 0.05 (-0.09; 0.19)  0.08 (0.06; 0.22)  0.06 (-0.07; 0.20)  0.05 (-0.09; 0.19) | -0.11 (-0.24; 0.02)  -0.08 (-0.20; 0.05)  -0.05 (-0.18; 0.08)  -0.07 (-0.19; 0.06) | **-0.14 (-0.26; -0.03)**  -0.08 (-0.19; 0.04)  -0.07 (-0.19; 0.04)  -0.08 (-0.19; 0.04) | **0.00**  **0.02**  0.05  0.05 | 0.02 (-0.03; 0.06)  0.00 (-0.05; 0.05)  -0.01 (-0.06; 0.04)  -0.01 (-0.06; 0.04) | 0.08 (-0.09; 0.26)  0.05 (-0.13; 0.22)  0.06 (-0.12; 0.24)  0.04 (-0.14; 0.21) | -0.11 (-0.27; 0.04)  -0.12 (-0.28; 0.03)  -0.12 (-027; 0.03)  -0.13 (-0.28; 0.03) | 0.03 (-0.11; 0.16)  -0.01 (-0.15; 0.13)  -0.04 (-0.17; 0.10)  -0.04 (-0.18; 0.10) | 0.63  0.47  0.24  0.30 |
| Plasma biomarkers of MVD composite score, per SD | 1  2  3A  3B | **-0.05 (-0.08; -0.02)**  **-0.04 (-0.01; -0.01)**  **-0.04 (-0.01; -0.01)**  **-0.03 (-0.06; -0.00)** | | **0.34 (0.21; 0.48**)  **0.31 (0.17; 0.44)**  **0.29 (0.16; 0.42)**  **0.26 (0.13; 0.39)** | -0.06 (-0.19; 0.08)  -0.01 (-0.13; 0.12)  0.04 (-0.08; 0.16)  0.05 (-0.07; 0.18) | **-0.13 (0.25; -0.02)**  -0.06 (-0.17; 0.05)  -0.04 (-0.15; 0.07)  -0.01 (-0.12; 0.10) | **0.00**  **0.00**  **0.00**  **0.00** | **-0.07 (-0.12; -0.03)**  **-0.12 (-0.16; -0.06)**  **-0.12 (-0.16; -0.07)**  **-0.11 (-0.16; -0.07)** | **0.27 (0.10; 0.44)**  **0.23 (0.06; 0.40)**  **0.22 (0.05; 0.38)**  **0.21 (0.04; 0.37)** | -0.13 (-0.28; 0.03)  -0.13 (-0.28; 0.02)  -0.11 (-0.26; 0.04)  -0.11 (-0.26; 0.04) | **-0.18 (-0.31; -0.05)**  **-0.22 (-0.36; -0.09)**  **-0.21 (-0.34; -0.08)**  **-0.20 (-0.33; -0.07)** | **0.00**  **0.00**  **0.00**  **0.00** |

Betas and 95% confidence intervals represent the difference in CSVD features composite score, retinal microvascular diameters composite score, or (composite score of) measure(s) of MVD (all per SD) per unit of total alcohol consumption or for none, moderate, or high versus light total alcohol consumption stratified by glucose metabolism status where a negative beta indicates less CSVD features, narrower retinal microvascular diameters, or less MVD (i.e. for the flicker light-induced increase in retinal microvascular diameters composite score, heat-induced skin hyperemia, UAE, or the plasma biomarkers of MVD composite score). Total alcohol consumption was entered in the models as a continuous variable (per unit, i.e. 10 g/day), as dummies (none, moderate or high versus light alcohol consumption) or (for the P-for trend analyses) as a categorical variable (none, light, moderate, and high alcohol consumption). The numerical values per SD are presented in the legend of Supplemental Table S6.

The number of participants with and without hypertension respectively are n=1,101 and n=974 for CSVD features, n=1,559 and n=1,162 for retinal microvascular diameters, n=1,173 and n=917 for flicker light-induced increase in retinal microvascular diameters, n=898 and n=619 for heat-induced skin hyperemia, n=1,788 and n=1,319 for UAE, and n=1,772 and n=1,306 for plasma biomarkers of MVD.

Variables per model: Model 1: crude; Model 2: model 1+ age, sex, glucose metabolism status, educational level; Model 3A: model 2 + waist circumference, smoking status, diet score; Model 3B: model 3A + office systolic blood pressure, total cholesterol / HDL cholesterol ratio, lipid-modifying medication, history of cardiovascular disease. Additionally, and only for heat-induced skin hyperemia, baseline skin blood was entered in model 1.

Bold denotes P-value<0.05.

* For respectively individuals with and without hypertension the betas of total alcohol consumption (per unit) in model 3B correspond with the following odds ratios (95% CI) for 30mg/24-hour greater UAE: 0.97 (0.94; 1.01) and 0.99 (0.94; 1.04).

Abbreviations: β, beta; CI, confidence interval; SD: standard deviation; CSVD, cerebral small vessel disease; UAE, urinary albumin excretion; MVD, microvascular dysfunction. HDL, high-density lipoprotein..

**Supplemental Table S15 Associations of total alcohol consumption with MVD measures, stratified by dyslipidemia status**

|  |  | |  |  |  |  |  |  |  |  |  |  |
| --- | --- | --- | --- | --- | --- | --- | --- | --- | --- | --- | --- | --- |
|  |  | | With hypercholesterolemia | | | | | Without hypercholesterolemia | | | | |
|  | Model | | Continuous | None vs. light | Moderate vs. light | High vs. light | P for trend | Continuous | None vs. light | Moderate vs. light | High vs. light | P for trend |
|  |  | | β (95% CI) | β (95% CI) | β (95% CI) | β (95% CI) | P-value | β (95% CI) | β (95% CI) | β (95% CI) | β (95% CI) | P-value |
| CSVD features composite score,  per SD | 1  2  3A  3B | 0.02 (-0.02; 0.06)  -0.01 (-0.05; 0.03)  -0.02 (-0.06; 0.02)  -0.01 (-0.06; 0.03) | | -0.02 (-0.19; 0.15)  0.02 (-0.15; 0.18)  0.02 (-0.14; 0.18)  -0.02 (-0.18; 0.15) | -0.03 (-0.19; 0.13)  -0.04 (-0.19; 0.10)  -0.05 (-0.20; 0.10)  -0.06 (-0.20; 0.09) | -0.05 (-0.18; 0.09)  -0.07 (-0.19; 0.06)  -0.09 (-0.21; 0.04)  -0.07 (-0.20; 0.05) | 0.62  0.23  0.13  0.29 | **0.09 (0.04; 0.14)**  0.02 (-0.03; 0.07)  0.02 (-0.04; 0.07)  0.02 (-0.03; 0.07) | 0.01 (-0.21; 0.24)  -0.02 (-0.23; 0.19)  -0.02 (-0.23; 0.19)  -0.04 (-0.26; 0.17) | **0.22 (0.02; 0.42)**  0.10 (-0.09; 0.28)  0.12 (-0.07; 0.30)  0.12 (-0.07; 0.31) | **0.29 (0.12; 0.47)**  0.10 (-0.07; 0.26)  0.08 (-0.09; 0.25)  0.10 (-0.08; 0.27) | **0.00**  0.16  0.23  0.13 |
| Retinal microvascular diameters composite score, per SD | 1  2  3A  3B | **-0.05 (0.09; -0.02)**  -0.02 (-0.06; 0.02)  -0.02 (-0.06; 0.02)  -0.01 (-0.05; 0.02) | | **0.15 (0.01; 0.29)**  0.06 (-0.09; 0.20)  0.04 (-0.11; 0.18)  0.05 (-0.10; 0.19) | **-0.17 (-0.30; -0.03)**  **-0.14 (-0.27; -0.01)**  **-0.13 (-0.27; -0.00)**  -0.11 (-0.24; 0.03) | -0.07 (-0.19; 0.05)  -0.06 (-0.18; 0.05)  -0.06 (-0.18; 0.06)  -0.04 (-0.15; 0.08) | **0.00**  0.06  0.10  0.19 | -0.03 (-0.07; 0.02)  0.02 (-0.02; 0.07)  0.02 (-0.03; 0.06)  0.02 (-0.03; 0.07) | 0.03 (-0.17; 0.24)  -0.02 (-0.23; 0.18)  -0.03 (-0.24; 0.18)  -0.02 (-0.23; 0.19) | -0.08 (-0.26; 0.10)  -0.05 (-0.23; 0.13)  -0.06 (-0.24; 0.13)  -0.05 (-0.23; 0.14) | -0.10 (-0.25; 0.06)  -0.03 (-0.19; 0.14)  -0.06 (-0.22; 0.11)  -0.04 (-0.20; 0.13) | 0.13  0.82  0.15  0.72 |
| Flicker light-induced increase in retinal diameters composite score, per SD | 1  2  3A  3B | -0.02 (-0.06; 0.02)  -0.03 (-0.07; 0.01)  -0.04 (-0.08; 0.01)  -0.03 (-0.08; 0.01) | | 0.01 (-0.16; 0.17)  0.01 (-0.16; 0.17)  0.01 (-0.15; 0.18)  -0.00 (-0.17; 0.16) | -0.06 (-0.22; 0.09)  -0.05 (-0.21; 0.10)  -0.05 (-0.20; 0.10)  -0.06 (-0.22; 0.09) | -0.13 (-0.26; 0.01)  -0.12 (-0.25; 0.02)  -0.13 (-0.26; 0.01)  -0.13 (-0.27; 0.01) | **0.04**  0.06  0.05  0.06 | **0.05 (0.00; 0.11)**  0.03 (-0.02; 0.09)  0.03 (-0.02; 0.09)  0.03 (-0.03; 0.09) | -0.07 (-0.31; 0.16)  -0.09 (-0.33; 0.15)  -0.09 (-0.33; 0.16)  -0.07 (-0.31; 0.17) | -0.14 (-0.34; 0.07)  -0.15 (-0.35; 0.06)  -0.14 (-0.34; 0.07)  -0.13 (-0.34; 0.08) | 0.11 (-0.07; 0.29)  0.07 (-0.12; 0.25)  0.08 (-0.11; 0.26)  0.07 (-0.12; 0.26) | 0.14  0.31  0.29  0.34 |
| Heat-induced skin hyperemia, per SD | 1  2  3A  3B | 0.02 (-0.02; 0.06)  -0.02 (-0.06; 0.02)  -0.03 (-0.07; 0.02)  -0.03 (-0.07; 0.02) | | -0.16 (-0.35; 0.02)  -0.06 (-0.24; 0.12)  -0.08 (-0.26; 0.10)  -0.09 (-0.27; 0.09) | -0.05 (-0.22; 0.12)  -0.00 (-0.17; 0.16)  -0.02 (-0.19; 0.14)  -0.02 (-0.18; 0.15) | -0.14 (-0.30; 0.01)  -0.07 (-0.22; 0.08)  -0.10 (-0.24; 0.05)  -0.09 (-0.24; 0.06) | 0.57  0.65  0.53  0.61 | -0.01 (-0.08; 0.06)  -0.03 (-0.10; 0.05)  -0.02 (-0.09; 0.06)  -0.01 (-0.09; 0.07) | 0.10 (-0.20; 0.39)  0.06 (-0.24; 0.35)  0.02 (-0.28; 0.32)  0.01 (-0.29; 0.31) | -0.04 (-0.30; 022)  -0.01 (-0.28; 0.25)  -0.06 (-0.32; 0.21)  -0.05 (-0.32; 0.22) | -0.15 (-0.38; 0.07)  -0.11 (-0.34; 0.12)  -0.10 (-0.33; 0.14)  -0.07 (-0.31; 0.17) | 0.07  0.23  0.34  0.52 |
| Logarithmically transformed UAE, per SD | 1  2  3A  3B | -0.01 (-0.04; 0.02)  -0.03 (-0.06; 0.00)  -0.03 (-0.06; 0.00)  -0.03 (-0.06; 0.00) | | 0.04 (-0.09; 0.17)  0.03 (-0.09; 0.16)  0.02 (-0.11; 0.15)  -0.01 (-0.14; 0.12) | -0.09 (-0.21; 0.04)  -0.05 (-0.17; 0.07)  -0.04 (-0.16; 0.08)  -0.06 (-0.18; 0.06) | **-0.13 (-0.24; -0.02)**  -0.07 (-0.17; 0.04)  -0.07 (-0.17; 0.04)  -0.07 (-0.18; 0.03) | **0.00**  0.08  0.12  0.17 | 0.03 (-0.01; 0.08)  0.00 (-0.04; 0.05)  0.00 (-0.05; 0.05)  0.00 (-0.05; 0.05) | **0.20 (0.00; 0.39)**  0.16 (-0.04; 0.35)  0.16 (-0.03; 0.35)  0.14 (-0.05; 0.33) | -0.15 (-0.32; 0.02)  -0.16 (-0.32; 0.01)  -0.13 (-0.30; 0.04)  -0.13 (-0.29; 0.04) | 0.00 (-0.15; 0.15)  -0.03 (-0.18; 0.12)  -0.03 (-0.18; 0.12)  -0.04 (-0.19; 0.12) | 0.11  0.07  0.08  0.10 |
| Plasma biomarkers of MVD composite score, per SD | 1  2  3A  3B | **-0.06 (-0.09; -0.03)**  **-0.06 (-0.09; -0.03)**  **-0.06 (-0.09; -0.03)**  **-0.05 (-0.08; -0.02)** | | **0.35 (0.23; 0.48)**  **0.31 (0.18; 0.44)**  **0.30 (0.18; 0.42)**  **0.27 (0.15; 0.39)** | -0.06 (-0.18; 0.06)  -0.01 (-0.13; 0.10)  0.01 (-0.10; 0.13)  0.02 (-0.09; 0.14) | **-0.14 (-0.25; -0.03)**  -0.08 (-0.18; 0.03)  -0.07 (-0.17; 0.03)  -0.04 (-0.14; 0.06) | **0.00**  **0.00**  **0.00**  **0.00** | -0.03 (-0.07; 0.02)  **-0.07 (-0.11; -0.03)**  **-0.07 (-0.11; -0.03)**  **-0.05 (-0.09; -0.01)** | **0.27 (0.08; 0.47)**  0.17 (-0.02; 0.35)  0.15 (-0.03; 0.33)  0.12 (-0.06; 0.30) | -0.11 (-0.28; 0.06)  -0.11 (-0.27; 0.05)  -0.06 (-0.22; 0.10)  -0.04 (-0.20; 0.11) | -0.16 (-0.31; 0.02)  **-0.22 (-0.37; -0.08)**  **-0.19 (-0.33; -0.05)**  **-0.16 (-0.30; 0.02)** | **0.00**  **0.00**  **0.00**  **0.00** |

Betas and 95% confidence intervals represent the difference in CSVD features composite score, retinal microvascular diameters composite score, or (composite score of) measure(s) of MVD (all per SD) per unit of total alcohol consumption or for none, moderate, or high versus light total alcohol consumption stratified by dyslipidemia status where a negative beta indicates less CSVD features, narrower retinal microvascular diameters, or less MVD (i.e. for the flicker light-induced increase in retinal microvascular diameters composite score, heat-induced skin hyperemia, UAE, or the plasma biomarkers of MVD composite score). Total alcohol consumption was entered in the models as a continuous variable (per unit, i.e. 10 g/day), as dummies (none, moderate or high versus light alcohol consumption) or (for the P-for trend analyses) as a categorical variable (none, light, moderate, and high alcohol consumption). The numerical values per SD are presented in the legend of Supplemental Table S6.

The number of participants with and without hypertension respectively are n=1,301 and n=774 for CSVD features, n=1,780 and n=941 for retinal microvascular diameters, n=1,350 and n=740 for flicker light-induced increase in retinal microvascular diameters, n=1,055 and n=462 for heat-induced skin hyperemia, n=2,060 and n=1,047 for UAE, and n=2,038 and n=1,040 for plasma biomarkers of MVD.

Variables per model: Model 1: crude; Model 2: model 1+ age, sex, glucose metabolism status, educational level; Model 3A: model 2 + waist circumference, smoking status, diet score; Model 3B: model 3A + office systolic blood pressure, use of antihypertensive medication, total cholesterol / HDL cholesterol ratio, history of cardiovascular disease. Additionally, and only for heat-induced skin hyperemia, baseline skin blood was entered in model 1.

Bold denotes P-value<0.05.

* For respectively individuals with and without hypercholesterolemia the betas of total alcohol consumption (per unit) in model 3B correspond with the following odds ratios (95% CI) for 30mg/24-hour greater UAE: 0.97 (0.94; 1.00) and 1.00 (0.96; 1.04).

Abbreviations: β, beta; CI, confidence interval; SD: standard deviation; CSVD, cerebral small vessel disease; UAE, urinary albumin excretion; MVD, microvascular dysfunction. HDL, high-density lipoprotein.

**Supplemental Table S16 Associations of total alcohol consumption with MVD measures, stratified by current** smoking status

|  |  | |  |  |  |  |  |  |  |  |  |  |
| --- | --- | --- | --- | --- | --- | --- | --- | --- | --- | --- | --- | --- |
|  |  | | Current smoker | | | | | Former or never smoker | | | | |
|  | Model | | Continuous | None vs. light | Moderate vs. light | High vs. light | P for trend | Continuous | None vs. light | Moderate vs. light | High vs. light | P for trend |
|  |  | | β (95% CI) | β (95% CI) | β (95% CI) | β (95% CI) | P-value | β (95% CI) | β (95% CI) | β (95% CI) | β (95% CI) | P-value |
| CSVD features composite score,  per SD | 1  2  3A  3B | 0.05 (-0.03; 0.13)  0.03 (-0.05; 0.11)  0.03 (-0.05; 0.11)  0.04 (-0.04; 0.12) | | 0.12 (-0.25; 0.50)  -0.02 (-0.37; 0.34)  -0.00 (-0.35; 0.34)  -0.10 (-0.44; 0.25) | **0.49 (0.09; 0.90)**  **0.44 (0.07; 0.81)**  **0.44 (0.07; 0.81)**  **0.41 (0.05; 0.77)** | 0.09 (-0.25; 0.42)  0.02 (-0.29; 0.33)  0.02 (-0.29; 0.32)  0.02 (-0.28; 0.33) | 0.76  0.62  0.67  0.37 | **0.04 (0.01; 0.08)**  -0.01 (-0.04; 0.03)  -0.02 (-0.05; 0.02)  -0.01 (-0.04; 0.03) | -0.03 (-0.18; 0.12)  -0.01 (-0.14; 0.13)  0.00 (-0.13; 0.14)  -0.01 (-0.15; 0.13) | -0.01 (-0.14; 0.12)  -0.06 (-0.18; 0.06)  -0.06 (-0.18; 0.06)  -0.06 (-0.18; 0.06) | 0.06 (-0.05; 0.17)  -0.03 (-0.13; 0.08)  -0.05 (-0.15; 0.06)  -0.03 (-0.14; 0.08) | 0.20  0.61  0.33  0.58 |
| Retinal microvascular diameters composite score, per SD | 1  2  3A  3B | -0.00 (-0.07; 0.07)  0.02 (-0.05; 0.08)  0.02 (-0.05; 0.08)  0.03 (-0.04; 0.10**)** | | 0.22 (-0.09; 0.52)  0.25 (-0.06; 0.55)  0.25 (-0.06; 0.55)  0.23 (-0.08; 0.53) | -0.14 (-0.47; 0.19)  -0.05 (-0.36; 0.27)  -0.03 (-0.35; 0.29)  -0.00 (-0.32; 0.32**)** | 0.15 (-0.13; 0.43)  0.21 (-0.06; 0.48)  0.21 (-0.06; 0.48)  0.25 (-0.03; 0.52) | 0.76  0.92  0.89  0.58 | **-0.05 (-0.08; -0.02)**  -0.01 (-0.05; 0.02)  -0.01 (-0.05; 0.02)  -0.01 (-0.04; 0.02) | 0.07 (-0.06; 0.20)  -0.02 (-0.14; 0.11)  -0.01 (-0.14; 0.11)  -0.00 (-0.13; 0.13) | **-0.14 (-0.25; -0.02)**  -0.11 (-0.22; 0.01)  -0.11 (-0.22; 0.01)  -0.08 (-0.20; 0.03) | **-0.12 (-0.21; -0.02)**  -0.09 (-0.19; 0.01)  -0.09 (-0.19; 0.01)  -0.07 (-0.17; 0.03) | **0.00**  0.07  0.07  0.14 |
| Flicker light-induced increase in retinal diameters composite score, per SD | 1  2  3A  3B | -0.01 (-0.08; 0.07)  -0.02 (-0.10; 0.06)  -0.02 (-0.10; 0.06)  -0.02 (-0.11; 0.06**)** | | -0.15 (-0.51; 0.21)  -0.18 (0.55; 0.20)  -0.17 (-0.55; 0.21)  -0.17 (-0.55; 0.21) | -0.15 (-0.53; 0.23)  -0.12 (-0.51; 0.27**)**  -0.11 (-0.50; 0.28)  -0.13 (-0.52; 0.27**)** | **-**0.32 (-0.64; 0.00**)**  -0.32 (-0.65; 0.00)  -0.32 (-0.64; 0.01)  -0.36 (-0.69; 0.02) | 0.16  0.19  0.20  0.15 | 0.01 (-0.02; 0.05)  -0.01 (-0.04; 0.03)  -0.01 (-0.05; 0.03)  -0.01 (-0.05; 0.03) | 0.02 (-0.12; 0.16)  -0.00 (-0.15; 0.15)  0.00 (-0.15; 0.15)  -0.01 (-0.15; 0.14) | -0.08 (-0.21; 0.06)  -0.08 (-0.21; 0.05)  -0.08 (-0.20; 0.06)  -0.08 (-0.21; 0.05) | 0.00 (-0.10; 0.12)  -0.01 (-0.13; 0.10)  -0.02 (-0.14; 0.10)  -0.02 (-0.13; 0.10) | 0.75  0.70  0.64  0.72 |
| Heat-induced skin hyperemia, per SD | 1  2  3A  3B | 0.01 (-0.07; 0.10)  -0.02 (-0.10; 0.07)  -0.02 (-0.10; 0.06)  -0.03 (-0.12; 0.06**)** | | 0.10 (-0.32; 0.53)  0.08 (0.35; 0.50)  0.07 (-0.35; 0.50)  -0.12 (-0.31; 0.55) | -0.29 (-0.74; 0.17)  -0.14 (-0.58; 0.30)  -0.14 (-0.58; 0.30)  -0.17 (-0.61; 0.28**)** | -0.09 (-0.50; 0.31)  -0.05 (-0.43; 0.33)  -0.06 (-0.44; 0.33)  -0.09 (-0.49; 0.31) | 0.25  0.51  0.50  0.30 | 0.01 (-0.03; 0.05)  -0.03 (-0.07; 0.01)  -0.03 (-0.07; 0.01)  -0.03 (-0.07; 0.02) | -0.15 (-0.32; 0.02)  -0.10 (-0.26; 0.07)  -0.09 (-0.26; 0.07)  -0.10 (-0.27; 0.07) | -0.02 (-0.17; 0.13)  0.00 (-0.15; 0.15)  -0.00 (-0.15; 0.15)  0.01 (-0.14; 0.15) | -0.16 (-0.29; 0.03)  -0.10 (-0.24; 0.03)  -0.11 (-0.24; 0.03)  -0.10 (-0.23; 0.04) | 0.31  0.48  0.46  0.57 |
| Logarithmically transformed UAE, per SD | 1  2  3A  3B* | -0.03 (-0.09; 0.03)  -0.05 (-0.11; 0.01)  -0.04 (-0.10; 0.02)  -0.04 (-0.10; 0.02**)** | | 0.07 (-0.21; 0.35)  -0.08 (0.34; 0.20)  -0.07 (-0.33; 0.20)  -0.06 (-0.32; 0.21) | **-0.31 (-0.61; -0.01)**  **-0.29 (-0.56; -0.01)**  -0.25 (-0.52; 0.02)  -0.27 (-0.54; 0.01**)** | **-0.24 (-0.49; -0.02)**  **-0.24 (-0.48; -0.01)**  -0.22 (-0.45; 0.01)  -0.23 (-0.47; 0.01) | **0.01**  0.06  0.08  0.06 | 0.01 (-0.02; 0.04)  -0.02 (-0.04; 0.01)  -0.02 (-0.05; 0.01)  -0.02 (-0.04; 0.01) | 0.07 (-0.04; 0.19)  0.07 (-0.05; 0.18)  0.07 (-0.04; 0.19)  0.04 (-0.07; 0.15) | -0.08 (-0.18; 0.03)  -0.06 (-0.16; 0.05)  -0.04 (-0.14; 0.06)  -0.05 (-0.16; 0.05) | -0.07 (-0.16; 0.03)  -0.04 (-0.13; 0.05)  -0.04 (-0.13; 0.06)  -0.04 (-0.13; 0.05) | **0.01**  0.08  0.08  0.16 |
| Plasma biomarkers of MVD composite score, per SD | 1  2  3A  3B | -0.04 (-0.19; 0.02)  -0.04 (-0.11; 0.02)  -0.03 (-0.09; 0.03)  -0.02 (-0.08; 0.04**)** | | **0.35 (0.07; 0.64)**  0.22 (-0.06; 0.51)  0.23 (-0.04; 0.50)  0.21 (-0.06; 0.49) | -0.02 (-0.32; 0.28)  0.01 (-0.28; 0.30)  0.06 (-0.22; 0.36)  0.08 (-0.20; 0.36**)** | -0.10 (-0.36; 0.16)  -0.10 (-0.35; 0.15)  -0.08 (-0.31; 0.17)  -0.04 (-0.28; 0.21) | **0.00**  **0.03**  0.05  0.13 | **-0.05 (-0.08; -0.02)**  **-0.07 (-0.10; -0.04)**  **-0.07 (-0.10; -0.04)**  **-0.06 (-0.09; -0.03)** | **0.33 (0.21; 0.44)**  **0.27 (0.16; 0.39)**  **0.27 (0.16; 0.38)**  **0.24 (0.14; 0.35)** | -0.09 (-0.19; 0.02)  -0.06 (-0.16; 0.04)  -0.02 (-0.12; 0.07)  -0.01 (-0.11; 0.08) | **-0.16 (-0.25; -0.07)**  **-0.13 (-0.22; -0.04)**  -**0.11 (-0.20; -0.02)**  -0.09 (-0.17; 0.00) | **0.00**  **0.00**  **0.00**  **0.00** |

Betas and 95% confidence intervals represent the difference in CSVD features composite score, retinal microvascular diameters composite score, or (composite score of) measure(s) of MVD (all per SD) per unit of total alcohol consumption or for none, moderate, or high versus light total alcohol consumption stratified by smoking status where a negative beta indicates less CSVD features, narrower retinal microvascular diameters, or less MVD (i.e. for the flicker light-induced increase in retinal microvascular diameters composite score, heat-induced skin hyperemia, UAE, or the plasma biomarkers of MVD composite score). Total alcohol consumption was entered in the models as a continuous variable (per unit, i.e. 10 g/day), as dummies (none, moderate or high versus light alcohol consumption) or (for the P-for trend analyses) as a categorical variable (none, light, moderate, and high alcohol consumption). The numerical values per SD are presented in the legend of Supplemental Table S6.

The number of participants with and without current smoking respectively are 236 and n=1,839 for CSVD features, n=333 and n=2,388 for retinal microvascular diameters, n=249 and n=1,841 for flicker light-induced increase in retinal microvascular diameters, n=176 and n=1,341 for heat-induced skin hyperemia, n=395 and n=2,712 for UAE, and n=391 and n=2,687 for plasma biomarkers of MVD.

Variables per model: Model 1: crude; Model 2: model 1+ age, sex, glucose metabolism status, educational level; Model 3A: model 2 + waist circumference, smoking status (former/never; only applicable for former or never smoker stratum), diet score; Model 3B: model 3A + office systolic blood pressure, use of antihypertensive medication, total cholesterol / HDL cholesterol ratio, lipid-modifying medication, history of cardiovascular disease. Additionally, and only for heat-induced skin hyperemia, baseline skin blood was entered in model 1.

* For respectively current and non-current smokers the betas of total alcohol consumption (per unit) in model 3B correspond with the following odds ratios (95% CI) for 30mg/24-hour greater UAE: 0.96 (0.90; 1.02) and 0.98 (0.96; 1.01).

Abbreviations: β, beta; CI, confidence interval; SD: standard deviation; CSVD, cerebral small vessel disease; UAE, urinary albumin excretion; MVD, microvascular dysfunction. HDL, high-density lipoprotein.

**Supplemental Table S17 Associations of total alcohol consumption with the total MVD composite score and MVD measures, additionally adjusted for physical activity, diabetic retinopathy, eGFR, and plasma biomarkers of low-grade inflammation**

|  |  | Total alcohol consumption | | | | |
| --- | --- | --- | --- | --- | --- | --- |
|  | Model | Continuous | None vs. light | Moderate vs. light | High vs. light | P for trend |
|  |  | β (95% CI) | β (95% CI) | β (95% CI) | β (95% CI) | P-value |
| Total MVD composite score, per SD | 3B+ physical activity, n=2,422  3B+ diabetic retinopathy, n=2,903  3B + eGFR, n=3,096  3B + biomarkers of LGI, n=3,092 | **-0.04 (-0.07; -0.02)**  **-0.04 (-0.07; -0.02)**  **-0.04 (-0.07; -0.02)**  **-0.04 (-0.07; -0.02)** | **0.12 (0.01; 0.23)**  0.08 (-0.02; 0.18)  **0.13 (0.03; 0.22)**  **0.11 (0.02; 0.21)** | **-0.10 (-0.20;- 0.00)**  **-0.10 (-0.19; -0.01)**  -0.08 (-0.17; -0.00)  **-0.10 (-0.18; -0.01)** | **-0.11 (-0.20; -0.02)**  **-0.13 (-0.21; -0.05)**  **-0.08 (-0.16; -0.00)**  **-0.11 (-0.19; -0.03)** | **0.00**  **0.00**  **0.00**  **0.00** |
| CSVD features composite score, per SD | 3B+ physical activity, n=1,614  3B+ diabetic retinopathy, n=1,743  3B + eGFR, n=2,061  3B + biomarkers of LGI, n=2,061 | -0.01 (-0.05; 0.03)  -0.00 (-0.04; 0.03)  0.00 (-0.03; 0.03)  -0.00 (-0.03; 0.03) | -0.01 (-0.16; 0.13)  -0.01 (-0.14; 0.12)  -0.02 (-0.14; 0.11)  -0.03 (-0.16; 0.10) | -0.03 (-0.16; 0.10)  -0.01 (-0.13; 0.10)  -0.00 (-0.12; 0.11)  0.00 (-0.11; 0.11) | -0.04 (-0.13; 0.10)  -0.03 (-0.13; 0.08)  -0.02 (-0.12; 0.09)  -0.02 (-0.12; 0.08) | 0.80  0.73  0.92  0.96 |
| Retinal microvascular diameters composite score, per SD | 3B+ physical activity, n=2,213  3B+ diabetic retinopathy, n=2,552  3B + eGFR, n=2,701  3B + biomarkers of LGI, n=2,696 | -0.00 (-0.04; 0.03)  0.00 (-0.03; 0.03)  0.00 (-0.03; 0.03)  -0.00 (-0.03; 0.03) | 0.03 (-0.10; 0.16**)**  0.02 (-0.10; 0.14)  0.02 (-0.10; 0.14)  0.02 (-0.10; 0.14) | -0.05 (-0.17; 0.07)  -0.08 (-0.19; 0.02)  -0.09 (-0.19; 0.02)  -0.08 (-0.19; 0.03) | -0.03 (-0.14; 0.07)  -0.03 (-0.13; 0.06)  -0.04 (-0.13; 0.06)  -0.04 (-0.13; 0.06) | 0.31  0.28  0.24  0.24 |
| Flicker light-induced increase in retinal diameters composite score, per SD | 3B+ physical activity, n=1,790  3B+ diabetic retinopathy, n=2,030  3B + eGFR, n=2,078  3B + biomarkers of LGI, n=2,074 | -0.02 (-0.05; 0.02)  -0.01 (-0.04; 0.03)  -0.01 (-0.05; 0.02)  -0.01 (-0.04; 0.03) | -0.03 (-0.18; 0.12)  -0.02 (-0.16; 0.11)  -0.03 (-0.16; 0.11)  -0.02 (-0.16; 0.11) | -0.06 (-0.20; 0.07)  -0.08 (-0.20; 0.05)  -0.09 (-0.22; 0.03)  -0.09 (-0.21; 0.04) | -0.05 (-0.17; 0.06)  -0.04 (-0.15; 0.07)  -0.06 (-0.17; 0.05)  -0.05 (-0.16; 0.06) | 0.46  0.52  0.33  0.44 |
| Heat-induced skin hyperemia, per SD | 3B+ physical activity, n=1,267  3B+ diabetic retinopathy, n=1,373  3B + eGFR, n=1,507  3B + biomarkers of LGI, n=1,506 | -0.02 (-0.06; 0.02)  -0.03 (-0.07; 0.01)  -0.02 (-0.06; 0.02)-0.02 (-0.06; 0.02) | -0.13 (-0.30; 0.04)  -0.07 (-0.23; 0.09)  -0.07 (-0.22; 0.09)  -0.08 (-0.23; 0.08) | -0.07 (-0.22; 0.09)  -0.02 (-0.16; 0.13)  0.00 (-0.14; 0.14)  -0.01 (-0.15; 0.13) | -0.12 (-0.26; 0.02)  -0.12 (-0.25; 0.01)  -0.08 (-0.21; 0.05)  -0.08 (-0.21; 0.04) | 0.46  0.25  0.53  0.55 |
| Logarithmically transformed UAE, per SD | 3B+ physical activity, n=2,408  3B+ diabetic retinopathy, n=2,610  3B + eGFR, n=3,082  3B + biomarkers of LGI, n=3,079 | -0.02 (-0.04; 0.01)  -0.02 (-0.04; 0.01)  -0.01 (-0.04; 0.01) -0.02 (-0.05; 0.01) | 0.08 (-0.04; 0.19)  0.03 (-0.08; 0.14)  0.04 (-0.07; 0.14)  0.03 (-0.07; 0.14) | **-0.11 (-0.22; -0.01)**  -0.06 (-0.16; 0.04)  -0.08 (-0.17; 0.02)  -0.08 (-0.18; 0.01) | -0.03 (-0.13; 0.07)  -0.05 (-0.14; 0.04)  -0.05 (-0.13; 0.04)  -0.06 (-0.15; 0.02) | 0.08  0.10  0.09  **0.04** |
| Plasma biomarkers of MVD composite score, per SD | 3B+ physical activity, n=2,392  3B+ diabetic retinopathy, n=2,590  3B + eGFR, n=3,071  3B + biomarkers of LGI, n=3,077 | **-0.05 (-0.08; -0.02)**  **-0.05 (-0.08; -0.03)**  **-0.04 (-0.06; -0.01) -0.05 (-0.08; -0.03)** | **0.25 (0.14; 0.37)**  **0.21 (0.11; 0.32)**  **0.25 (0.15; 0.35)**  **0.23 (0.13; 0.32)** | 0.02 (-0.08; 0.13)  -0.00 (-0.10; 0.09)  0.02 (-0.07; 0.11)  -0.00 (-0.09; 0.09) | -0.08 (-0.17; 0.02)  **-0.09 (-0.18; -0.01)**  -0.03 (-0.11; 0.05)  -0.07 (-0.15; 0.00) | **0.00**  **0.00**  **0.00**  **0.00** |

Betas and 95% confidence intervals represent the difference in the total MVD composite score or per measure of MVD (all per SD) per unit of total alcohol consumption or for none, moderate, or high versus light total alcohol consumption where a negative beta indicates less total MVD, less CSVD features, narrower retinal microvascular diameters, or less MVD estimated from the flicker light-induced increase in retinal microvascular diameters composite score, heat-induced skin hyperemia, UAE, or the plasma biomarkers of MVD composite score. Total alcohol consumption was entered in the models as a continuous variable (per unit, i.e. 10 g/day), as dummies (none, moderate or high versus light alcohol consumption) or (for the P-for trend analyses) as a categorical variable (none, light, moderate, and high alcohol consumption). For all analyses the value of one SD was numerically comparable to the value of one SD that was presented in the legend of Table 2.

Variables in model 3B: age, sex, glucose metabolism status, educational level, waist circumference, smoking status, diet score; office systolic blood pressure, use of antihypertensive medication, total cholesterol / HDL cholesterol ratio, lipid-modifying medication, prior cardiovascular disease and, only for heat-induced skin hyperemia, baseline skin blood flow.

Bold denotes P-value<0.05.

Abbreviations: β, beta; CI: confidence interval; CSVD, cerebral small vessel disease; SD: standard deviation; eGFR, estimated glomerular filtration rate; MVD, microvascular dysfunction; HDL, high-density lipoprotein.

**Supplemental Table S18 Associations of total alcohol consumption with CSVD features, retinal microvascular diameters, and measures of MVD, where educational level was replaced with income level (model 3C) or occupational status (model 3D); glucose metabolism status was replaced with fasting plasma glucose (model 3E), or 2-hour post load (model 3F) or HbA1c (model 3G); or office systolic blood pressure was replaced with office diastolic blood pressure (model 3H), 24-hour ambulatory systolic blood pressure (model 3I) or 24-hour ambulatory diastolic blood pressure (model 3J)**

|  |  | Total alcohol consumption | | | | |
| --- | --- | --- | --- | --- | --- | --- |
|  | Model | Continuous | None vs. light | Moderate vs. light | High vs. light | P for trend |
|  |  | β (95% CI) | β (95% CI) | β (95% CI) | β (95% CI) | P-value |
| Total MVD composite score, per SD | 3C, n= 2,382  3D, n= 2,594  3E, n= 3,119  3F, n= 2,880  3G, n= 3,112  3H, n= 3,120  3I, n= 2,770  3J, n= 2,770 | -**0.05 (-0.07; -0.02)**  -**0.05 (-0.07; -0.02)**  -**0.05 (-0.07; -0.02)**  **-0.03 (-0.06; -0.00)**  **-0.04 (-0.06; -0.01)**  **-0.04 (-0.07; -0.02)**  **-0.05 (-0.08; -0.02)**  **-0.05 (-0.08; -0.02)** | **0.14 (0.02; 0.25)**  **0.12 (0.01; 0.22)**  **0.13 (0.04; 0.23)**  0.09 (-0.02; 0.20)  **0.12 (0.02; 0.21)**  **0.11 (0.00; 0.22)**  **0.12 (0.01; 0.22)**  **0.12 (0.01; 0.22)** | **-0.14 (-0.25; -0.04)**  -0.08 (-0.18; 0.01)  **-0.10 (-0.19; -0.01)**  -0.09 (-0.18; 0.01)  **-0.09 (-0.18; -0.00)**  -0.10 (-0.20; 0.00)  **-0.13 (-0.22; -0.04)**  **-0.13 (-0.22; -0.04)** | **-0.12 (-0.21; -0.03)**  **-0.12 (-0.21; -0.04)**  **-0.12 (-0.20; -0.04)**  -0.09 (-0.18; 0.01)  **-0.11 (-0.19; -0.03)**  **-0.11 (-0.20; 0.02)**  **-0.14 (-0.22; -0.05)**  **-0.14 (-0.22; -0.05)** | **0.00**  **0.00**  **0.00**  **0.00**  **0.00**  **0.00**  **0.00**  **0.00** |
| CSVD features composite score, per SD | 3C, n= 1,628  3D, n= 1,770  3E, n= 2,075  3F, n= 1,961  3G, n= 2,071  3H, n= 2,075  3I, n= 1,852  3J, n= 1,852 | -0.00 (-0.04; 0.03)  0.01 (-0.03; 0.04)  -0.00 (-0.04; 0.03)  0.01 (-0.03; 0.04)  -0.00 (-0.03; 0.03)  -0.00 (-0.03; 0.03)  -0.02 (-0.05; 0.02)  -0.02 (-0.05; 0.02) | 0.00 (-0.15; 0.15)  -0.03 (-0.17; 0.11)  -0.02 (-0.15; 0.11)  -0.02 (-0.15; 0.12)  -0.02 (-0.14; 0.11)  -0.02 (-0.15; 0.11)  -0.02 (-0.16; 0.12)  -0.02 (-0.15; 0.12) | -0.04 (-0.17; 0.09)  -0.04 (-0.16; 0.08)  0.00 (-0.12; 0.11)  -0.00 (-0.12; 0.11)  0.00 (-0.11; 0.12)  -0.00 (-0.11; 0.11)  -0.05 (-0.17; 0.07)  -0.04 (-0.16; 0.08) | -0.02 (-0.13; 0.10)  -0.02 (-0.13; 0.08)  -0.03 (-0.13; 0.08)  -0.00 (-0.11; 0.10)  -0.02 (-0.12; 0.08)  -0.02 (-0.12; 0.08)  -0.06 (-0.17; 0.05)  -0.06 (-0.17; 0.05) | 0.72  0.86  0.78  0.87  0.86  0.83  0.35  0.33 |
| Retinal microvascular diameters composite score, per SD | 3C, n= 2,092  3D, n= 2,280  3E, n= 2,720  3F, n= 2,515  3G, n= 2,715  3H, n= 2,721  3I, n= 2,424  3J, n= 2,424 | -0.01 (-0.05; 0.02)  -0.00 (-0.04; 0.03)  -0.01 (-0.03; 0.02)  -0.00 (-0.03; 0.03)  -0.00 (-0.03; 0.03)  0.00 (-0.03; 0.03)  -0.01 (-0.04; 0.02)  -0.01 (-0.04; 0.03) | 0.01 (-0.13; 0.15)  0.00 (-0.13; 0.13)  0.03 (-0.08; 0.15)  0.03 (-0.10; 0.15)  0.03 (-0.09; 0.15)  0.01 (-0.11; 0.12)  0.01 (-0.11; 0.13)  0.00 (-0.12; 0.13) | -0.11 (-0.23; 0.01)  -0.11 (-0.22; 0.01)  -0.09 (-0.20; 0.02)  -0.07 (-0.18; 0.04)  -0.09 (-0.20; 0.02)  -0.09 (-0.19; 0.02)  -0.10 (-0.21; 0.02)  -0.11 (-0.21; 0.01) | -0.07 (-0.18; 0.04)  -0.06 (-0.16; 0.04)  -0.05 (-0.14; 0.05)  -0.03 (-0.13; 0.07)  -0.04 (-0.14; 0.05)  -0.04 (-0.13; 0.06)  -0.04 (-0.14; 0.06)  -0.04 (-0.14; 0.06) | 0.12  0.18  0.11  0.27  0.14  0.30  0.30  0.33 |
| Flicker light-induced increase in retinal microvascular diameters composite score, per SD | 3C, n= 1,629  3D, n= 1,757  3E, n= 2,090  3F, n= 1,942  3G, n= 2,087  3H, n= 2,090  3I, n= 1,862  3J, n= 1,862 | -0.01 (-0.05; 0.03)  -0.00 (-0.04; 0.03)  -0.01 (-0.04; 0.03)  -0.01 (-0.05; 0.02)  0.01 (-0.04; 0.03)  -0.01 (-0.04; 0.03)  -0.02 (-0.06; 0.02)  -0.02 (-0.06; 0.02) | -0.03 (-0.19; 0.12)  -0.08 (-0.23; 0.08)  -0.03 (-0.17; 0.10)  -0.03 (-0.17; 0.12)  -0.04 (-0.17; 0.10)  -0.03 (-0.16; 0.11)  -0.05 (-0.19; 0.10)  -0.05 (-0.19; 0.09) | -0.10 (-0.24; 0.04)  -0.05 (-0.19; 0.08)  -0.09 (-0.21; 0.04)  -0.08 (-0.20; 0.05)  -0.08 (-0.21; 0.04)  -0.08 (-0.20; 0.04)  -0.15 (-0.28; -0.02)  **-0.15 (-0.28; -0.02)** | -0.06 (-0.18; 0.07)  -0.04 (-0.16; 0.08)  -0.06 (-0.17; 0.05)  -0.05 (-0.17; 0.06)  -0.05 (-0.16; 0.06)  -0.05 (-0.16; 0.06)  -0.11 (-0.23; 0.00)  -0.11 (-0.22; 0.01) | 0.47  0.97  0.42  0.48  0.53  0.45  0.10  0.13 |
| Heat-induced skin hyperemia, per SD | 3C, n= 1,168  3D, n= 1,271  3E, n= 1,517  3F, n= 1,395  3G, n= 1,516  3H, n= 1,517  3I, n= 1,335  3J, n= 1,335 | -0.03 (-0.07; 0.01)  -0.04 (-0.08; 0.01)  -0.03 (-0.06; 0.01)  -0.01 (-0.05; 0.03)  -0.02 (-0.06; 0.02)  -0.02 (-0.06; 0.02)  -0.03 (-0.07; 0.01)  -0.03 (-0.07; 0.01) | -0.02 (-0.20; 0.16)  -0.08 (-0.25; 0.10)  -0.06 (-0.22; 0.09)  -0.04 (-0.20; 0.13)  -0.07 (-0.22; 0.09)  -0.07 (-0.22; 0.09)  -0.08 (-0.24; 0.09)  -0.08 (-0.24; 0.09) | -0.07 (-0.23; 0.09)  -0.00 (-0.15; 0.15)  -0.03 (-0.17; 0.11)  0.01 (-0.14; 0.15)  -0.01 (-0.15; 0.13)  -0.01 (-0.15; 0.13)  -0.02 (-0.17; 0.13)  -0.01 (-0.16; 0.14) | -0.11 (-0.25; 0.04)  -0.11 (-0.25; 0.03)  -0.11 (-0.23; 0.02)  -0.05 (-0.19; 0.08)  -0.09 (-0.22; 0.03)  -0.08 (-0.21; 0.04)  **-0.15 (-0.29; -0.02)**  **-0.14 (-0.28; -0.01)** | 0.17  0.34  0.28  0.63  0.43  0.52  0.13  0.18 |
| Logarithmically transformed UAE, per SD | 3C, n= 2,381  3D, n= 2,598  3E, n= 3,106  3F, n= 2,868  3G, n= 3,100  3H, n= 3,107  3I, n= 2,760  3J, n= 2,760 | -0.02 (-0.05; 0.01)  -0.03 (-0.05; 0.00)  -0.02 (-0.05; 0.00)  -0.00 (-0.03; 0.03)  -0.02 (-0.04; 0.01)  -0.02 (-0.04; 0.01)  **-0.03 (-0.06; -0.00)**  -0.03 (-0.06; 0.00) | 0.03 (-0.09; 0.16)  0.04 (-0.08; 0.15)  0.04 (-0.06; 0.14)  0.01 (-0.10; 0.12)  0.03 (-0.07; 0.13)  0.04 (-0.06; 0.15)  0.05 (-0.06; 0.16)  0.06 (-0.05; 0.17) | -0.07 (-0.18; 0.04)  -0.08 (-0.18; 0.02)  -0.09 (-0.18; 0.01)  -0.04 (-0.15; 0.06)  -0.07 (-0.17; 0.02)  -0.07 (-0.17; 0.03)  -0.08 (-0.18; 0.02)  -0.06 (-0.16; 0.04) | -0.05 (-0.16; 0.04)  -0.07 (-0.16; 0.02)  -0.07 (-0.15; 0.02)  -0.02 (-0.11; 0.07)  -0.05 (-0.13; 0.03)  -0.05 (-0.14; 0.03)  -0.07 (-0.16; 0.01)  -0.06 (-0.15; 0.03) | 0.11  **0.04**  **0.02**  0.48  0.08  0.05  **0.01**  **0.03** |
| Plasma biomarkers of MVD composite score, per SD | 3C, n= 2,357  3D, n= 2,575  3E, n= 3,077  3F, n= 2,838  3G, n= 3,070  3H, n= 3,078  3I, n= 2,732  3J, n= 2,732 | **-0.05 (-0.08; -0.02)**  **-0.06 (-0.09; -0.03)**  **-0.05 (-0.07; -0.03)**  **-0.05 (-0.08; -0.02)**  **-0.05 (-0.07; -0.02)**  **-0.05 (-0.08; -0.03)**  **-0.05 (-0.08; -0.03)**  **-0.05 (-0.08; -0.02)** | **0.28 (0.16; 0.40)**  **0.27 (0.16 0.38)**  **0.25 (0.15; 0.35)**  **0.18 (0.07; 0.29)**  **0.24 (0.14; 0.33**)  **0.24 (0.14; 0.34)**  **0.24 (0.13; 0.34)**  **0.24 (0.13; 0.34)** | -0.03 (-0.14; 0.07)  0.02 (-0.08; 0.12)  -0.00 (-0.09; 0.09)  -0.03 (-0.12; 0.07)  0.01 (-0.08; 0.10)  0.00 (-0.09; 0.09)  -0.03 (-0.13; 0.06)  -0.03 (-0.13; 0.07) | -0.07 (-0.16; 0.02)  -0.08 (-0.17; 0.01)  **-0.08 (-0.16; -0.00)**  **-0.10 (-0.18; -0.01)**  -0.07 (-0.15; 0.02)  -0.08 (-0.16; 0.00)  -0.09 (-0.17; 0.00)  -0.09 (-0.18; 0.00) | **0.00**  **0.00**  **0.00**  **0.00**  **0.00**  **0.00**  **0.00**  **0.00** |

Betas and 95% confidence intervals represent the difference in the total MVD composite score or per measure of MVD (all per SD) per unit of total alcohol consumption or for none, moderate, or high versus light total alcohol consumption where a negative beta indicates less total MVD, less CSVD features, narrower retinal microvascular diameters, or less MVD estimated from the flicker light-induced increase in retinal microvascular diameters composite score, heat-induced skin hyperemia, UAE, or the plasma biomarkers of MVD composite score. Total alcohol consumption was entered in the models as a continuous variable (per unit, i.e. 10 g/day), as dummies (none, moderate or high versus light alcohol consumption) or (for the P-for trend analyses) as a categorical variable (none, light, moderate, and high alcohol consumption). For all analyses the value of one SD was numerically comparable to the value of one SD that was presented in the legend of Table 2.

Variables in model 3B: age, sex, glucose metabolism status (where applicable), educational level (where applicable), waist circumference, smoking status, diet score, office systolic blood pressure (where applicable), use of antihypertensive medication, total cholesterol / HDL cholesterol ratio, lipid-modifying medication, prior cardiovascular disease and, only for heat-induced skin hyperemia, for baseline skin blood flow.

Bold denotes P-value<0.05.

Abbreviations: β, beta; CI: confidence interval; CSVD, cerebral small vessel disease; CRAE, central retinal arteriolar equivalent; CRVE, central retinal venular equivalent; SD: standard deviation; sICAM-1, soluble intercellular adhesion molecule-1; sVCAM-1, soluble vascular adhesion molecule-1; sE-selectin, soluble E-selectin; vWF, von Willebrand factor; MVD, microvascular dysfunction; HDL, high-density lipoprotein.
